# Trends in Cardiac Biomarker Testing in China for Patients with Acute Myocardial Infarction, 2001 to 2011: China PEACE-Retrospective AMI Study

**DOI:** 10.1371/journal.pone.0122237

**Published:** 2015-04-20

**Authors:** Lijuan Zhan, Frederick A. Masoudi, Xi Li, Shuang Hu, Arjun K. Venkatesh, John A. Spertus, Zhenqiu Lin, Nihar R. Desai, Jing Li, Harlan M. Krumholz, Lixin Jiang, China PEACE Collaborative Group

**Affiliations:** 1 National Clinical Research Center of Cardiovascular Diseases, State Key Laboratory of Cardiovascular Disease, Fuwai Hospital, National Center for Cardiovascular Diseases, Chinese Academy of Medical Sciences and Peking Union Medical College, Beijing, People's Republic of China; 2 Division of Cardiology, University of Colorado Anschutz Medical Campus, Aurora, Colorado, United States of America; 3 Robert Wood Johnson Clinical Scholars Program, Yale School of Medicine, New Haven, Connecticut, United States of America; 4 Department of Internal Medicine, Yale School of Medicine, New Haven, Connecticut, United States of America; 5 Department of Emergency medicine, Yale School of Medicine, New Haven, Connecticut, United States of America; 6 Saint Luke’s Mid America Heart Institute/University of Missouri-Kansas City, Kansas City, Missouri, United States of America; 7 Center for Outcomes Research and Evaluation, Yale-New Haven Hospital, New Haven, Connecticut, United States of America; 8 Section of Cardiovascular Medicine, Yale School of Medicine, New Haven, Connecticut, United States of America; 9 Department of Health Policy and Management, Yale School of Public Health, New Haven, Connecticut, United States of America; Virginia Commonwealth University, UNITED STATES

## Abstract

**Objectives:**

To describe trends in the availability of biomarker testing in Chinese hospitals and how practice complies with established standards for the diagnosis of acute myocardial infarction (AMI).

**Background:**

Cardiac biomarker testing is standard in high-income countries, but little is known about the availability and use of cardiac biomarker testing in low- and middle-income countries (LMICs) such as China.

**Methods:**

Based on a nationally representative sample of Chinese hospitals in 2001, 2006 and 2011, we describe the temporal trends and regional differences in the hospital capability and rates of use of cardiac biomarker testing, as well as the variation in use across hospitals with testing capability, for patients labeled with the diagnosis of AMI.

**Results:**

We sampled 175 hospitals (162 participated in the study) and 18,631 AMI admissions. 14,370 patients were included in analysis of biomarker use. The proportion of hospitals with biomarker testing capability was 57.4% in 2001 (25.0% troponin and 32.4% creatine kinase MB fraction (CK-MB) only) and 96.3% (81.4% troponin and 14.9% CK-MB only) in 2011. The proportion of hospitals with troponin testing capability in 2011 was significantly higher in urban compared with rural hospitals (96.8% vs. 71.4%, p< 0.001). In 2011, only 55.9% of hospitals with troponin testing capability (71 out of 127 hospitals) used the assay for more than 80% of their patients with AMI. Among hospitals with either biomarker testing capability, there was marked variation in use in both rural (from 7.1% to 100.0% of patients) and urban hospitals (from 57.9% to 100.0% of patients). In 2011, 36.1% of the patients with AMI did not have troponin tested and 4.9% did not have either biomarker measured.

**Conclusions:**

The recommended biomarker tests for AMI diagnosis are not universally available and the testing is not consistently applied when it is available in China.

**Trial Registration:**

ClinicalTrials.gov NCT01624883

## Introduction

Acute myocardial infarction (AMI) is one of the leading causes of death worldwide [1]. Cardiac biomarkers have been emphasized as central to the diagnosis and risk stratification strategy for AMI by numerous clinical practice guidelines [2–3]. Additionally, the Universal Definitions of AMI, which articulate a preference for cardiac troponin [4–12] due to its higher sensitivity, specificity, and diagnostic and prognostic value compared with creatine kinase MB fraction (CK-MB) [5,13–15]. Troponin testing in the setting of a suspected AMI is a Class I recommendation in contemporary guidelines [2–3,10].

The implementation of these recommendations may present challenges to many low- and middle-income countries (LMICs), where resources are constrained and the majority of the population receives care at hospitals without advanced cardiovascular services [16]. The capacity to test biomarkers requires equipment, reagents and trained personnel; hence these tests might be economically prohibitive in resource-poor settings. Hospital variation in the capability to provide biomarker testing and underuse of available testing could result in missed diagnoses of AMI (particularly non-ST-segment elevation myocardial infarction, NSTEMI), inequities in the delivery of evidence-based treatments, inefficiency in health care delivery, and potential harm to patients. China is a LMIC that faces resource constraints in delivering health care, particularly in rural areas. Also like many LMICs, China is in the midst of substantial transitions in both the epidemiology of non-communicable diseases and the structure of its health care system [16–19]; the burden of AMI is expected to increase exponentially in the next decade [19]. Accordingly, there is a pressing need to understand how the current recommendations align with the availability of biomarker testing services in Chinese hospitals and how actual practice complies with these standards for the diagnosis of AMI.

The China Patient-centered Evaluative Assessment of Cardiac Events Retrospective Study of Acute Myocardial Infarction (China PEACE-Retrospective AMI Study) has shown that only 50.1% (6140 of 12,264) patients (21.4% in 2001, 45.5% in 2006, and 66.4% in 2011) had their troponin level tested among patients with ST-segment elevation myocardial infarction (STEMI) [20]. It highlights the underutilization of biomarker testing in China during the past decade. However, there is currently a paucity of information about the availability of biomarker testing in China and particularly about how practice may diverge from recommendations. To address this issue, we evaluated changes in hospital capability to perform biomarker testing and the extent to which available biomarker testing was used for patients with AMI in a nationally representative cohort of Chinese hospitals between 2001 and 2011 using the China PEACE-Retrospective AMI Study. We also examined regional differences in hospital capability and hospital adherence to clinical guidelines for the use of cardiac biomarkers. The insights garnered from these investigations could form the foundation of strategies to improve the diagnosis, care and outcomes of patients with AMI in China and provide insights for other LMICs.

## Methods

### Study Design

**Fig 1:**
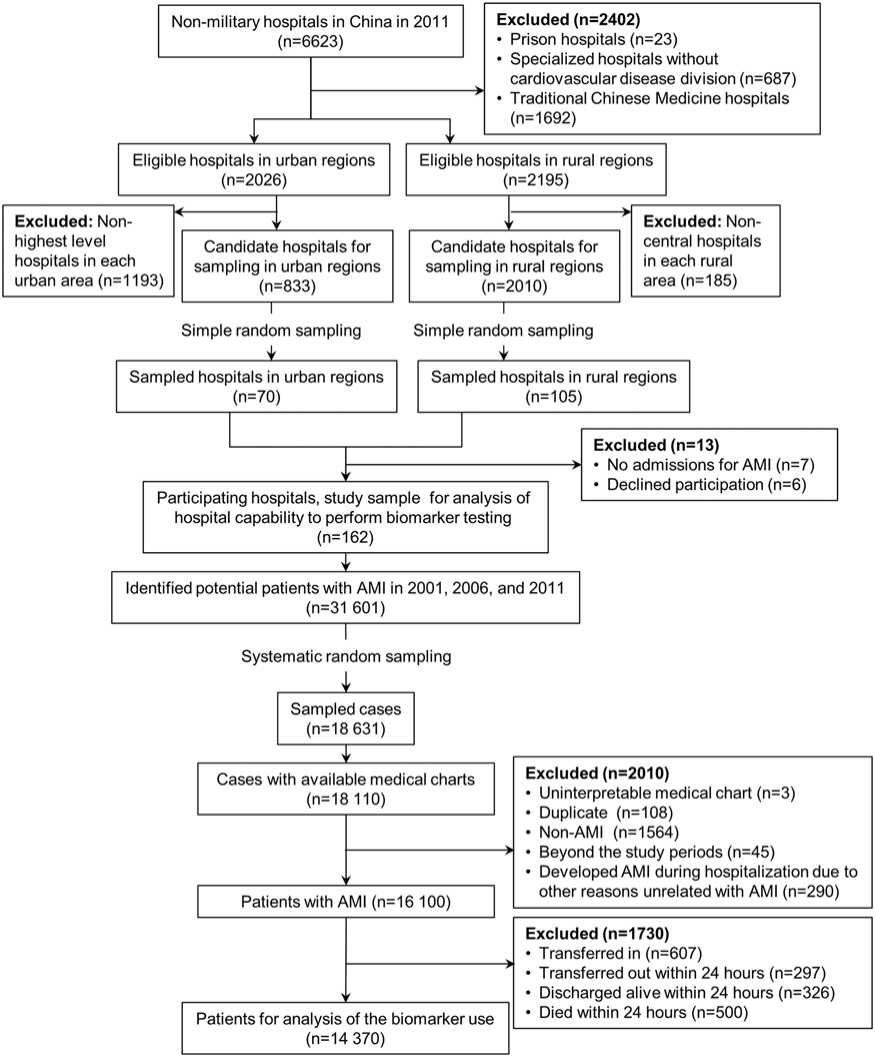
Hospital and patient sample. AMI = acute myocardial infarction.

The design of the China PEACE-Retrospective AMI Study has been published previously [20–21]. In brief, we selected a nationally representative sample of hospitals to reflect diverse sites of care in China and identified the hospitalization for AMI using two-stage random sampling (Fig 1). In the first stage, we identified hospitals using a simple random sampling procedure within each of the five study strata: Eastern-rural, Central-rural, Western-rural, Eastern-urban, and Central/Western-urban regions, since hospital volumes and clinical capacities differ between urban and rural areas as well among the three official economic-geographic regions (Eastern, Central, and Western) of Mainland China. Since the majority of hospitals in China are publicly owned and administered, hospital closure is rare. We selected representative hospitals from 2011 to reflect current practices and trace this cohort backward to 2006 and 2001 to describe temporal trends. A total of 175 hospitals (105 rural and 70 urban) were sampled, 7 did not have admissions for AMI and 6 declined to participate. We then used systematic random sampling to identify hospitalizations from the selected hospitals with a principal discharge diagnosis of AMI during 2001, 2006, and 2011. In the second stage, we drew cases based on the local hospital database for patients with AMI in each year at each sampled hospital using systematic random sampling procedures. We included patients who were diagnosed with AMI throughout China. Patients with confirmed diagnosis of AMI made by local hospitals were identified using International Classification of Diseases—Clinical Modification codes, including versions 9 (410.xx) and 10 (I21.xx), when available or through principal discharge diagnosis terms. The definition does not require biomarkers. Data were collected via standardized central medical chart abstraction using standardized data definitions. We employed rigorous monitoring at each stage to ensure data fidelity [21]. Data abstraction quality was monitored by randomly auditing 5% of the medical records, with overall variable accuracy exceeding 98%.

The institutional characteristics were collected via an electronic questionnaire completed by investigators at each participating hospital to identify the organizational factors that might influence patient management.

The central ethics committee at the China National Center for Cardiovascular Diseases (NCCD) approved this study. All collaborating hospitals accepted the central ethics approval except for five hospitals, which obtained local approval by internal ethics committees. The study is registered at www.clinicaltrials.gov (NCT01624883).

The Chinese government, who provided financial support for the study, had no role in the design or conduct of the study; in the collection, management, analysis, and interpretation of the data; or in the preparation or approval of the manuscript.

### Study Sample

Fig 1 shows the hospital and patient sample. To determine the capability of hospitals to perform biomarker testing, we analyzed all participating hospitals. To examine the use of biomarker testing, we excluded patients with AMI who were transferred in, or transferred out within 24 hours of hospitalization because we lack the information about their entire hospitalization for AMI. In addition, we excluded patients who died or discharged within 24 hours after admission, as their hospitalizations might be too short to permit biomarker testing. To evaluate the proportions of patients receiving biomarker testing at the hospital-level, we analyzed hospitals with at least 5 cases during a study year.

### Variables and Definition

#### Capability of hospitals to perform biomarker testing

We assessed the capability of hospitals to perform testing of either biomarker (troponin or CK-MB) and in strata according to whether the hospital had the capability to measure neither biomarker; CK-MB only; or troponin. A hospital was considered to have the capability of testing for a biomarker if, in that study year, at least one patient had a biomarker level recorded during the sampling period.

#### Rate of use of cardiac biomarker testing among capable hospitals

We assessed the rate of biomarker testing during the time that the hospitals had the capability to perform biomarker testing, which was defined as a period that started when the first test was performed in the respective hospital. The annual testing rate for a biomarker was calculated as the number of hospitalizations with lab test results of each biomarker testing divided by the total number of sampled AMI hospitalizations after the first test was performed in that year. This strategy enabled accurate assessments of the frequency of biomarker testing once it was confirmed that such testing was available and allowed an accurate assessment of hospitals’ testing rates if the capacity had been newly acquired.

### Statistical Analysis

Continuous variables were described as median values with interquartile ranges (IQR), and categorical variables were described as frequencies with percentages. We employed Wilcoxon rank sum tests for continuous variables, and the chi-square tests for categorical variables. We described the distribution of the biomarker use rate among hospitals with testing capability. We used box plots to display variation in the biomarker use rate across capable hospitals. All tests of statistical significance were two-sided, with a *p*< 0.05 considered statistically significant. Statistical analysis was performed using SAS software (version 9.2, SAS Institute, Cary, NC) and R software (version 3.0.1).

## Results

### Hospital Characteristics

Table 1 presents the characteristics of the 162 participating hospitals in 2011. The hospitals were distributed across Mainland China, included all 22 provinces, 5 autonomous regions and 3 of the 4 municipalities. The majority (92.9%) of rural hospitals were secondary while the majorities (92.1%) of urban hospitals were tertiary. Furthermore, 24.2% of rural hospitals and 87.3% of urban hospitals were equipped with cardiac catheterization laboratories. Of the urban hospitals, 50.8% also had the capability to perform on-site coronary artery bypass grafting (CABG).

**Table 1:** Hospitals characteristics (2011).

	Overall	Rural hospitals	Urban hospitals	P value
Total No. 162	n (%) / median (IQR)	n (%) / median (IQR)	n (%) / median (IQR)	
**Level of hospital**				< 0.001
Secondary	97 (59.9%)	92 (92.9%)	5 (7.9%)	
Tertiary	65 (40.1%)	7 (7.1%)	58 (92.1%)	
**Type of hospital**				< 0.001
Teaching	93 (57.4%)	39 (39.4%)	54 (85.7%)	
**CCU in hospital**	94 (58.0%)	38 (38.4%)	56 (88.9%)	< 0.001
**No. of beds in CCU, median (IQR)**	7 (5,10)	4 (4,5)	7 (6,12)	0.001
**No. of beds in CCU**				
≤ 5	28 (17.3%)	20 (20.2%)	8 (12.7%)	
6–10	43 (26.5%)	15 (15.2%)	28 (44.4%)	
>10	23 (14.2%)	3 (3.0%)	20 (31.7%)	
No CCU	68 (42.0%)	61 (6.6%)	7 (11.1%)	
**Cath lab in hospital**	79 (48.8%)	24 (24.2%)	55 (87.3%)	< 0.001
**No. of qualifiedcardiac interventionists**	3 (2,5)	1 (0,3)	4 (3,6)	0.030
**CABG be performed in hospital**	33 (20.4%)	1 (.0%)	32 (50.8%)	< 0.001
**Independent emergency department**	151 (93.2%)	91(91.9%)	60 (95.2%)	0.413
**Economic-geographic region**				< 0.001
Central	48 (29.6%)	35 (35.4%)	13 (20.6%)	
Eastern	64 (39.5%)	32(32.3%)	32 (50.8%)	
Western	50 (30.9%)	32 (32.3%)	18 (28.6%)	
**Routine diagnostic test of CK-MB for suspected ACS**				0.211
No	10 (6.2%)	8 (8.1%)	2 (3.2%)	
Yes	148 (91.4%)	88 (88.9%)	60 (95.2%)	
Unknown	4 (2.5%)	3 (3.0%)	1 (1.6%)	
**Average time delay in reporting results on CK-MB, hour**	1 (1,2)	1 (1,2)	1 (1,2)	0.064
**Routine diagnostic test of troponin for suspected ACS**				0.009
No	31 (19.1%)	27 (27.3%)	4 (6.3%)	
Yes	128 (79.0%)	69 (69.7%)	59 (93.7%)	
Unknown	3 (1.9%)	3 (3.0%)	0	
**Average time delay in reporting results on troponin, hour**	1 (1,2)	1 (1,2)	1 (1,2)	

ACS = acute coronary syndrome; CABG = coronary artery bypass grafting; Cath lab = catheterization lab; CCU = Coronary Care Unit; CK-MB = creatine kinase MB fraction; IQR = interquartile range

### Biomarker Testing Capability 2001–2011

**Fig 2:**
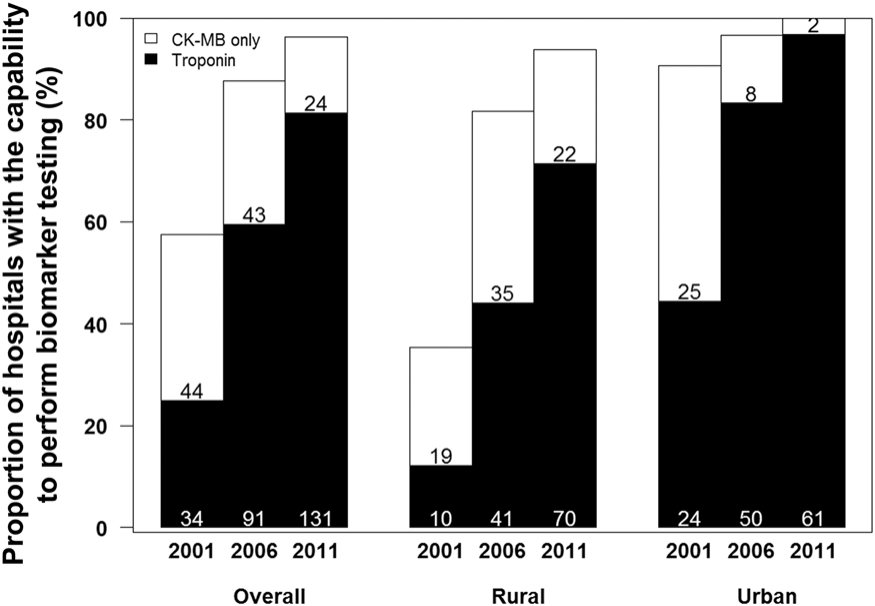
Temporal trends in proportion of hospitals with the capability to perform biomarker testing (n = 162). CK-MB = creatine kinase MB fraction.

The proportion of hospitals with the capability to perform either biomarker test for AMI increased significantly over time (Fig 2), from 57.4% in 2001 (25.0% using troponin and 32.4% using CK-MB only) to 96.3% (81.4% troponin, 14.9% CK-MB only) in 2011. By 2011, either biomarker test was available in all urban (100.0%) and almost all rural hospitals (93.9%). However, troponin testing was consistently less available in rural compared with urban hospitals in 2001(12.2% vs. 44.4%, *p*< 0.001), 2006 (44.1% vs. 83.3%, *p*< 0.001) and 2011 (71.4% vs. 96.8%, *p*< 0.001).

### Biomarker Use in Patients

**Fig 3:**
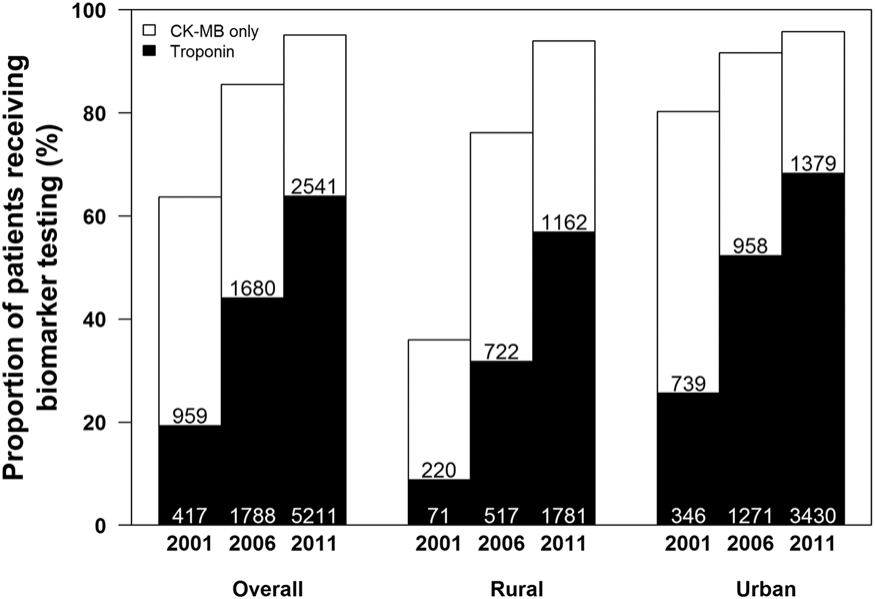
Temporal trends in proportion of patients receiving cardiac biomarker testing.

Among patients with AMI, the proportion receiving any biomarker test (either CK-MB or troponin) increased significantly during the study period from 63.7% in 2001 to 95.1% in 2011 (Fig 3). There was a rapid growth in the proportions of patients receiving either biomarker test in rural hospitals (35.9% in 2001 to 94.0% in 2011, *p*< 0.001). The use of troponin increased from 19.3% of patients in 2001 to 63.9% of patients in 2011 with a significant difference between rural and urban regions in 2011 (56.9% vs. 68.3%, *p*< 0.001). In 2011, 36.1% of patients with AMI in our study sample did not have troponin tested and 4.9% were not tested with either biomarker.

We assessed the proportion of patients with different AMI type (STEMI and NSTEMI). The proportions of patients receiving either biomarker test were not significantly lower for STEMI than for NSTEMI in 2001 (63.4% vs. 67.3%, *p* = 0.321) and in 2006 (85.1% vs. 88.7%, *p* = 0.046), same in 2011 (95.1% vs. 94.9, *p* = 0.696). The proportions of patients receiving troponin test were same for STEMI and NSTEMI in 2001 (19.3 vs. 19.4%, *p* = 0.976), lower for STEMI than for NSTEMI (43.0% vs. 52.8%, *p*< 0.001) in 2006 and in 2011 (63.2% vs. 67.3%, *p* = 0.002).

### Variability in Biomarker Use in Hospitals with Measurement Capability

**Fig 4:**
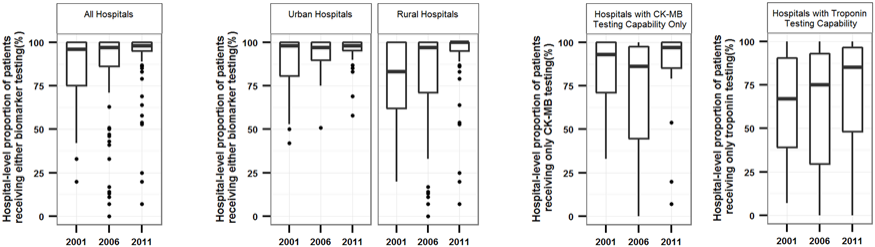
Hospital-level proportions of patients receiving biomarker testing among hospitals with testing capability. In all hospitals (A), by rural and urban location (B) and by the biomarker tests available (C).

Among 162 hospitals (99 rural and 63 urban), the number of hospitals with 5 or more cases and the capability to perform either biomarker test was 67 (23 rural and 44 urban) in 2001, 121 (65 rural and 56 urban) in 2006 and 146 (84 rural and 62 urban) in 2011. In hospitals with the capability to perform either biomarker test, the proportion of patients that received biomarker testing varied, although the average proportion increased over time (Fig 4A). In 2011, the hospital-level biomarker use rate for rural hospitals ranged from 7.1% to 100.0% (median 100.0%) while use in urban hospitals ranged from 57.9% to 100.0% (median 98.1%) (Fig 4B). In 2011, only 54.3% of troponin-capable hospitals (69 out of 127 hospitals) used the assay for more than 80% of their patients with AMI (Fig 4C).

Box and whisker plots showing the distribution of hospital-level proportions of patients receiving biomarker testing among capable hospitals in each study year. Central bar indicates median; top and bottom of box, 75th and 25th percentiles, respectively; and whiskers with minimum 1.5×IQR. The demarcation line for outlier was defined as 1.5×IQR

## Discussion

Our study shows that biomarker testing for the diagnosis of AMI, a routine consideration in higher-income countries and key component of the Universal Definition of AMI [4,9], is neither universally available nor consistently applied when it is available in China. Our study, conducted in the first large cohort of Chinese hospitals to examine national trends, reveals that despite a significant increase in the proportion of hospitals with biomarker testing capacity over the last decade, almost one third of patients with AMI did not undergo troponin testing in 2011. Furthermore, we identified substantial regional differences in hospital capability to perform troponin testing, an association that may be explained by the comparative lack of biomarker testing capacity in rural hospitals that persists in 2011. In addition, among the hospitals with biomarker testing capacity, there was substantial variability in the use of these important diagnostic tools. Specifically, in 2011, only half of troponin-capable hospitals used the assay for more than 80% of their patients with AMI.

Our study identifies important opportunities to expand biomarker use and improve current definitions used for the diagnosis of AMI. The Third Universal Definition of AMI, which is produced by European Society of Cardiology/ American College of Cardiology/ American Heart Association/ World Heart Federation [12], asserts that biomarkers are an essential element for the diagnosis of AMI. While the publication of this new definition in 2012 may have led to changes in higher-income countries, its application in resource-poor environments could be challenging because biomarker testing capacity is sometimes limited, and the tests are comparatively costly. Additionally, troponin testing is likely to be of particular importance in the identification of NSTEMI, a diagnosis that was observed in fewer than 20% of patients with AMI in our study. To bridge this “diagnosis gap” caused by a troponin-based case definition [22], the WHO definition of MI attempted to adapt the Universal Definition to lower resource settings where the biomarker assays may not always be available. The experience in China, with a significantly growing population with AMI, persistent gaps in diagnosis and treatment, and substantial resource constraints is similar to other LMICs. The insights from this study are therefore invested the potential implication of extrapolation into other LMICs as well.

A worsening risk profile for coronary heart diseases (CHD) and rising AMI mortality in the past decade in China, particularly in the rural population, heralds a need for increasing capacity to identify AMI promptly and accurately [23–24]. Standardized definitions accompanied by a strong emphasis on troponin testing are essential for the proper recognition and risk stratification of patients with AMI. Laboratories should move as rapidly as feasible to follow the standards of troponin testing and implement protocols to insure appropriate testing, early in the course of hospitalization of suspected AMI cases. Although one major barrier to the general availability of troponin assays among rural hospitals has traditionally been the lack of resources, China’s recent health care reforms have made substantial investments in local health institutions and health-worker training [25–26]. Allocation of resources to rural hospitals for standardization of assays, education of trained personnel, quality control of laboratory systems, and confirmation of diagnostic thresholds should all be the focus of future initiatives to ensure rapid diffusion of troponin testing in China. In parallel, efforts to ensure that clinicians are able to understand and integrate the results of these tests will be important to ensure the optimal use, as indiscriminate use could lead to unintended consequences that undermine the value of greater test availability. Ultimately, the balance of costs and potential consequences of excessive troponin testing with the real-world challenges of resource-poor environments requires further study.

Even among Chinese hospitals with the capability to perform biomarker testing, use varied markedly, limiting the early identification and management of some patients with AMI. Chinese Society of Cardiology of Chinese Medical Association and Editorial Board of Chinese Journal of Cardiology convened a consensus conference in 2008 and published 2010 China National Guideline for STEMI guideline to endorse the 2007 Universal Definition of AMI [27–28], including the use of troponin testing as essential to AMI diagnosis. Despite these recommendations, our study shows that the biomarker, particularly troponin level was not routinely measured for patients with AMI in many hospitals with testing capability in 2011. This may hinder diagnosis and risk stratification of patients with NSTEMI, for example, troponin-positive and CK-MB-negative patients have a higher risk profile but are less likely to receive guideline-recommended acute pharmacologic treatment because of the oversight of clinically meaningful early biomarker abnormalities [29]. Our study suggests that doctors were not selectively avoiding use of biomarker testing according to whether patients presented as STEMI or NSTEMI. The difference was significant but modest in size. Furthermore, a greater variation in biomarker use was observed in rural hospitals, where clinicians might simply be unaware of the Universal Definition of AMI and prior work also suggests that insufficient awareness of current guidelines or limited acceptance of troponin testing as the preferred marker of choice may be impeding the adoption of the Universal Definition of AMI in other LMICs [30–33]. Finally, as an older biomarker, CK-MB had consistently higher use rate than troponin, which suggested some physicians have not yet transitioned to troponin as the preferred biomarker. The variation in the availability and use of troponin testing observed in our study limits the applicability of the Third Universal Definition of AMI in China [10], and suggests a role for a guideline that is sensitive to the real-world challenges in resource-poor environments and, in parallel, efforts to make biomarker measurements more widely available are needed. As written, the WHO definition includes a category of MI (category B), which is to be applied whenever there is incomplete information on cardiac biomarkers together with symptoms of ischemia and the development of unequivocal pathological Q waves. In addition, another category (category C) corresponds to those with probable MI and can be applied when individuals with MI may get delayed access to medical services and/or unavailability of electrocardiography and/or laboratory assay of cardiac biomarkers [22]. Thus, the WHO definition does not necessitate the presence of positive biomarkers to establish a diagnosis of MI, unlike the Third Universal Definition of AMI. Chinese guidelines for the diagnosis of AMI, which have historically leaned heavily on those adopted in Europe and the United States, should reflect that biomarker testing is not always available. Consequently, the WHO definition can be applied across all regions of China regardless of the availability of biomarker testing capacity. Given the nuanced nature of such guidelines that allow for the diagnosis of AMI in circumstances when biomarker testing is unavailable, quality improvement efforts, such as consensus process for diagnosis, continuous monitoring and improvement of “doctoring” skills (e.g., electrocardiogram reading, history taking, physical examination) are essential to ensure that accurate diagnosis are made. There may be harm accruing to undiagnosed patients, but this analysis can only point to the importance of assessing that possibility in future work. For now, it may be best to initiate quality improvement efforts particularly targeted at patients with suspected AMI, but without ST-segment elevation. Besides, in hospitals without CK-MB capacity, the efforts should be made to establish troponin assay and standardization, while in hospitals with CK-MB capacity, it is reasonable to replace CK-MB with troponin testing given favorable testing characteristics and performance. Moreover, cost-effective point-of-care troponin assays were reported to be timesaving, simple to manipulate and comparable to quantitative troponin in predictive value [34–36]. These assays may be potential alternatives to laboratory troponin testing to enable early diagnosis and triage for patients with acute chest pain.

Since troponin findings have been established as a standard worldwide because of the sensitivity and specificity of these tests, health resources could be better directed as consequence of more accurate diagnosis and decreased mortality rates for China, which has one fifth of the world population with increasing morbidity of MI. In the long run, under the circumstances of the coexistence of opportunity and pressing needs, China should focus the efforts on homogenous availability and sufficient use of troponin testing. China has launched huge health-care reforms and recently doubled annual expenditures for health care trying to transform money and insurance coverage into cost-effective and high-quality services. China-PEACE platform will provide new opportunities to consistently evaluate how these efforts transform improvements in quality of care in terms of use of biomarkers testing in patients with AMI [21].

### Limitations

Our study should be interpreted in the context of certain limitations. First, we determined whether hospitals had biomarker testing capabilities according to availability of biomarker data in their medical charts from each hospital in each study year. We did not survey hospitals about their biomarker testing capabilities or the out-of-pocket costs associated with these tests. Second, we were unable to assess the quality of laboratories conducting biomarker assays, or their ability to expeditiously return results to ordering clinicians, both of which may impact clinicians’ adoption of biomarker testing. Third, patients in this study were all diagnosed with AMI instead of suspected acute coronary syndrome (ACS) and the relevance to patients suspected of ischemia is not known. Moreover, the patients in this study without biomarker testing were diagnosed based on clinical criteria. Therefore, we are assessing biomarker use in patients that doctors labeled as having an AMI. Fourth, we cannot determine why some patients were not tested, and given that most patients were diagnosed with AMI as STEMI, it is likely that some NSTEMI may not have been identified and measured in this dataset. Future studies are needed to investigate the use of troponin testing among patients with suspected of ACS, the timeliness of troponin testing, and the efficiency and quality with which troponin tests are used and reported.

## Conclusions

This nationally representative observational study demonstrated that the hospital capability to perform biomarker testing in China increased significantly over the last decade. However, many rural hospitals still do not have the capability to measure biomarkers, especially troponin, which is considered the international standard for AMI diagnosis. These findings signal a need for work to determine the best approaches to diagnosis in resource-poor settings, with full consideration of the costs and benefits of testing.

## Supporting Information

S1 Appendix Membership of the China PEACE Collaborative Group. (DOCX)

S2 Appendix China PEACE-Retrospective AMI Study consultants. (DOCX)

S3 Appendix Determination and validation of AMI type. (DOCX)

S1 Protocol China PEACE-Retrospective AMI Study design. (PDF)

S1 Protocol Suppl China PEACE-Retrospective AMI Study design supplemental materials. (DOCX)
