# Supplementary material for: Trends in Cardiac Biomarker Testing in China for Patients with Acute Myocardial Infarction, 2001 to 2011: China PEACE-Retrospective AMI Study
Source: PLoS One. 2015 Apr 20;10(4):e0122237. doi: 10.1371/journal.pone.0122237 (PMC4404305; doi:10.1371/journal.pone.0122237)
Supplement: S1 Appendix — (DOCX) [file pone.0122237.s001.docx]

# China PEACE-Retrospective AMI Study site investigators by hospital

Aba Tibetan and Qiang Autonomous Prefecture People's Hospital, Shiping Weng, Shuying Xie; Affiliated Hospital of Guiyang Medical College, Lirong Wu, Jiulin Chen; Affiliated Hospital of Hainan Medical College, Tianfa Li, Jun Wang; Affiliated Zhongshan Hospital of Dalian University, Qin Yu, Xiaofei Li; Alxa League Central Hospital, Zhong Li, Shiguo Hao, Yuzhen Zhang, Xuemei Wu; Baiquan County People's Hospital, Yachen Zhang, Zhifeng Liu; Biyang People's Hospital, Zhongxin Wang, Hao Jia; Bortala Mongol Autonomous Prefecture People's Hospital, Bayin Bate, Badeng Qiqige; Changda Hospital Of Anshan, Xiang Jin, Ting Cai; Chengwu County People's Hospital, Fengqin Liu, Dayong Xu; Chenxi County People's Hospital, Xuejin He, Shui Yang; Chongren County People's Hospital, Chun Yuan, Jiping Wang; County People's Hospital of Jinning, Lihua Gu, Lin Li, Shijiao Chen; Dalian Municipal Central Hospital, Yongchao Zhi, Lili Sun; Dao County People's Hospital, Shengcheng Zhou, Lingjiao Jin; Daofu County People's Hospital, Yong Leng, Liangchuan Zhang, Tianyun Deng; Dingyuan County People's Hospital of Anhui Province, Yuanjin Wang, Wenhua Zhang, Xinmin Ma; Dongyang People's Hospital, Weimin Li, Liang Lu, Xuan Ge; Dulong and Nu Autonomous County People's Hospital of Gongshan, Xiaoping Wu, Yanming He; Dunhua City Hospital of Jilin Province, Fanju Meng, Jia Li; Fenghuang County People's Hospital, Dexi Liao, Guangyong Liu; Fengshan County People’s Hospital, Wen Long, Xiangwen Chen; Fourth Hospital of Baotou City, Baohong Zhang, Yonghou Yin, Bin Tian; Fourth People's Hospital of Zigong City, Yong Yi, Chaoyong Wu; Fugu County People's Hospital of Shaanxi Province, Baoqi Liu, Zhihui Zhao, Haiming Li; Fujian Provincial Hospital, Yansong Guo, Xinjing Chen; Fuling Center Hospital of Chongqing City, Liquan Xiang, Lin Ning; Gannan County People's Hospital, Mei Chen, Xin Jin, Guiling Li; General Hospital of the Yangtze River Shipping, Xiuqi Li, Xing’an Wu; Gongcheng Yao Autonomous County People's Hospital, Congjun Tan, Mingfang Feng, Meili Wang; Guangchang County People's Hospital, Liangfa Wen, Xiang Fu, Qunxing Xie; Guilin People's Hospital, Wei Zhang, Yanni Zhuang, Hua Lu;Guiping People's Hospital, Jiaqian Lu, Yu Huang; Haerbin 242 Hospital, Yin Zhou, Qiuling Hu; Haiyan People's Hospital, Chunhui Xiao, Xiaoli Hu; Heling Ge Er County People's Hospital, Yongshuan Wu, Qiuli Wang; Helong Municipal People's Hospital, Youlin Xu, Xuefei Yu; Henan Provincial People's Hospital, Chuanyu Gao, Jianhong Zhang, You Zhang; Heze Municipal Hospital, Wentang Niu, Xiaolei Ma, Yong Wang; HGKY Group Company General Hospital, Xiaowen Pan, Yanlong Liu; Hua Xin Hospital First Hospital of Tsinghua University, Lifu Miao, Yanping Yin, Zhiying Zhang; Huairen People's Hospital, Shutang Feng; Huayin People's Hospital, Aiping Wang, Jiangli Zhang, Feipeng Li; Huaying People's Hospital, Hong Wang; Hunchun Hospital, Lijun Yu, Xinxin Zhao; Huizhou Municipal Central Hospital, Yuansheng Shen, Zhiming Li, Lizhen He; Hunan Province Mawangdui Hospital, Zhiyi Rong, Wei Luo; Ji'an Municipal Central People's hospital, Xueqiao Wang; Jianghua Yao Autonomous County People's Hospital, Rongjun Wan, Jianglin Tang, Guanghan Wu; Jiangsu Haimen People's Hospital, Jie Wu, Bin Xu; Jiangxi Provincial People's Hospital, Qing Huang, Xiaohe Wu; Jiangzi County People's Hospital, Sang Ge, Pian Pu, Pingcuo Duoji; Jilin Province People's Hospital, Hui Dai, Yuming Du, Wei Guo; Jilin Integrated Traditional Chinese & Western Medicine Hospital, Jilin Province, Jianping Shi; Jinghai County Hospital, Peihua Zhao, Jingsheng Sun; Jingxi County People's Hospital, Hongxiang Li, Wen Liang, Wen Qin; Jingxing County Hospital, Zhiwen Dong, Zhenhai Zhao; Jingzhou Central Hospital, Xin Li, Qin Xu; Jiuquan City People’s Hospital, Yaofeng Yuan, Zhirong Li; Jixi People's Hospital of The Jixi Municipal People's Hospital Medical Group, Jinbo Gao; Jize County Hospital, Qiu’e Guo; Kangbao County People's Hospital, Ruiqing Zhao, Guangjun Song; Keshiketengqi Hospital of Chifeng City, Lize Wang, Haiyun Song; Lanping Bai and Pumi Autonomous County People's Hospital, Jinwen He, Jinming He; Laoting County Hospital, Keyong Shang, Chang jiang Liu, Kuituan Xi; Liaoyang Central Hospital, Rihui Liu, Peng Guo; Liaoyuan Central Hospital, Chaoyang Guo, Xiangjun Liu, Rujun Zhao, Zeyong Yu; Lindian County Hospital, Wenzhou Li, Xudong Jing, Huanling Wang; Linxiang People's Hospital, Xiyuan Zhao, Chao Zhang, Long Chen; Liujiang County People's Hospital, Meifa Wei, Yan Liu, Shengde Chen; Longyan First Hospital, Kaihong Chen, Yong Fang, Ying Liao; Luancheng County Hospital, Junli Wang, Tianyu Liu, Suzhe Cheng; Lucheng People's Hospital, Yunke Zhou, Xiaoxia Niu, Huifang Cao; Luchuan County People's Hospital, Zebin Feng, Min Feng; Luxi County People's Hospital, Feilong Duan, Haiming Yi; Luyi County People's Hospital, Yuanxun Xu, Anran Guo; Macheng People's Hospital, Xianshun Zhou, Hongzhuan Cai, Peng Zheng; Mengcheng First People's Hospital, Gaofeng Guo; Menglian Lahu dai wa autonomous counties People's Hospital, Xiang Li; Min County People's Hospital, Minwu Bao, Yuhong Liu; Nanjing First Hospital, Shaoliang Chen, Haibo Jia, Hongjuan Peng; Nan’an Hospital, Duanping Dai, Shaoxiong Hong; Nantong Third People's Hospital, Song Chen, Dongya Zhang, Ying Wang; Nanyang Central Hospital, Yudong Li, Jianbu Gao, Shouzhong Yang; Ningwu County People's Hospital, Junhu An; Peking University People's Hospital, Chenyang Shen, Yunfeng Liu; Peking University Shenzhen Hospital, Chun Wu, Huan Qu, Saiyong Chen; People's Hospital of Jingyu, Yuhui Lin, Dehai Jiao; People's Hospital of Yueqing City, Manhong Wang, Qiu Wang; Pianguan County People's Hospital, Yingliang Xue, Ruijun Zhang; Puding County People's Hospital, Cheng Yuan, Lei Wu; Qinghai Red Cross Hospital, Jianqing Zhang, Chunmei Wei, Yanmei Shen; Qinshui County People's Hospital, Hehua Zhang, Hongmei Pan, Yong Gao; Qinyang People's Hospital, Xiaowen Ma, Yanli Liang, Tianbiao Wang; Queshan County People's Hospital, Daguo Zhao; Quzhou People's Hospital, Xiaoming Tu, Zhenyan Gao; Rongjiang County People's Hospital, Fangning Wang, Qiang Yang; Rudong County People's Hospital, Xiaoping Kang, Jianbin Fang, Dongmei Liu; Ruyang County People's Hospital, Chengning Shen, Mengfei Li; Shangluo Central Hospital, Yingmin Guan, Wenfeng Wang, Ting Xiao; Shangqiu Changzheng People's Hospital, Qian Wang; Shaoyang County People's Hospital, Fengyun Jiang, Kaiyou Wu; Shengsi People's Hospital, Songguo Wang; Shenyang Weikang Hospital, Xujie Fu, Shu Zhang,Lifang Gao; Shougang Shuicheng Iron & Steel (Group) Co., Ltd. General Hospital, Min Zhang, Kai Fu, Xiaojing Duan; Shuangshan Hospital Of Anshan, Rui Xiao, Ruixia Wu, Bin Li; Siziwang County People's Hospital, Hongtu Zhang, Yuerong Ma, Zhonghui Cao; Sunan Yugur Autonomous County People's Hospital, Zhansheng Ba, Wanhai Fu; Taizhou Hospital of Zhejiang Province, Jianjun Jiang, Yafei Mi, Weiwei Zhou; The Affiliated Hospital of Beihua University, Feng Sun, Qi Zhang, Shiyu Zheng; The Fifth People's Hospital of Dalian, Jing Zhang, Yang Zhong; The First Affiliated Hospital of Hebei North University, Fangjiang Li, Xiaoyuan Wang; The First Affiliated Hospital of Henan University of Science & Technology, Pingshuan Dong, Laijing Du, Wei Liu; The First Affiliated Hospital Of Jia Mu Si University, Zhaofa He, Meihua Jin; The First Hospital of Fuzhou City, Ting Jiang, Zhuoyan Chen; The First Hospital of Xi’an, Manli Cheng, Yuqiang Ji; The First People's Hospital of Danzhou, Youhua Zhou, Jvyuan Li; The First People's Hospital of Guangzhou, Yizhi Pan, Jian Liu; The First People's Hospital of Guangyuan, Tianxun Wang, Ping Yang; The Fourth People's Hospital of Shangqiu Shi, Guiyu Huang, Jianjun Pan,Qingliang Cai,Qianying Wang; The General Hospital of Yongzhou, Hunan Province, Mingli Lv; The people's hospital of Wuchuan, Yuanming Yi, Xuelian Deng; The People's Hospital of Yuanling, Wenhua Chen, Rong Cai; The People's Hospital of Zhijiang City, Bing Zhang; The Second Affiliated Hospital of Harbin Medical University, Bo Yu, Yousheng Xu, Zhengqiu Wang; The Second Affiliated Hospital of Kunming Medical University, Jun Shu, Ge Zhang, Kai Li; The Second Central Hospital of Baoding City, Guang Ma, Puxia Suo; The Second People's Hospital of Liaoyuan City, Aimin Zhang, Yongfen Kang; Tianjin Medical University General Hospital, Zheng Wan,Yuemin Sun, Bo Bian; Tibet Autonomous Region People's Hospital, Xuejun Hu, Dawa Ciren; Tongchuan Mining Bureau Central Hospital, Guojiong Jia, Jieli Pan; Tongliang County People's Hospital, Guofu Li, Hongliang Zhang, Longliang Zhan; Tongliao City Horqin District First People's Hospital, Junping Fang, Xinli Yu; Ulanqab Central Hospital, Dacheng Wang, Dajun Liu, Xinhong Cao; Wencheng County People's Hospital, Yi Tian, Haisheng Zhu,Wanchuan Liu; Wuhai People's Hospital, Zhaohai Zhou, Lei Shi; Wuhu Second People's Hospital, Wuwang Fang, Manxin Chen; Wulate County People's Hospital, Fuqin Han,Jianye Fu,Yunmei Wang; Wuqiang County People's Hospital, Binglu Liu, Yanliang Zhang,Xiupin Yuan; Wuyishan Municipal Hospital, Qingfei Lin, Yun Chen; Xiangtan County People's Hospital, Yuliang Zhu, Zhiqiang Cai; Xing County People's Hospital, Xingping Li, Lirong Ao; Xingshan County People's Hospital, Shubing Wu, Hui Zhang; Xinmi First People's Hospital, Fusheng Zhao, Guangming Yang; Xinshao County People's Hospital, Renfei Liu, Wenwei Ai; Xiuwu County People's Hospital, Jianbao Chang,Haijie Zhao; Xuanhan County People's Hospital, Qijun Ran, Xuan Ma; Xupu County People's Hospital, Shijun Jiang, Xiaochun Shu; Yanggao County People's Hospital, Zhiru Peng, Yan Han; Yanqing County Hospital, Jianbin Wang, Li Yang; Ying County People's Hospital, Yu Shen, Xingcun Shang; Yitong Manchu Autonomous County First People's Hospital, Haifeng Wang; Yongxing County People's Hospital, Hongyan Li, Zhisong Liao, Yang Cao; Yuanzhou District People's Hospital of Guyuan City, Xiaoping Gao, Meiying Cai, Lining You; Yuncheng Central Hospital, Xuexin Li, Shuqin Li, Yingjia Li; Yunlong County People's Hospital, Jianxun Yang, Song Ai, Jianfei Ma; Yuyao People's Hospital, Lailin Deng; Zhangjiachuan Hui Autonomous County First People's Hospital, Keyu Wang, Shitang Gao, Jian Guan; Zhouning County Hospital, Banghua He, Youyi Lu; Zhuoni County People's Hospital, Weirong Yang, Hong Li; Zhuozi County People's Hospital, Zhizhong Zhang, Xiaohong Chi; Zuoyun County People's Hospital, Ru Duan, Guangli Wang.
