# Supplementary material for: Trends in Cardiac Biomarker Testing in China for Patients with Acute Myocardial Infarction, 2001 to 2011: China PEACE-Retrospective AMI Study
Source: PLoS One. 2015 Apr 20;10(4):e0122237. doi: 10.1371/journal.pone.0122237 (PMC4404305; doi:10.1371/journal.pone.0122237)
Supplement: S3 Appendix — (DOCX) [file pone.0122237.s003.docx]

# Determination and validation of acute myocardial infarction (AMI) type (ST-segment elevation myocardial infarction or non ST-segment elevation myocardial infarction)

AMI type (ST-segment elevation myocardial infarction or non ST-segment elevation myocardial infarction, STEMI and NSTEMI) was determined by the combination of clinical discharge diagnosis terms and electrocardiogram results. If the local diagnosis was not definitive, cardiologists at the coordinating center reviewed the medical record and electrocardiogram to determine. We considered left bundle branch block (LBBB) as a STEMI equivalent.

A total of 300 medical records were randomly selected and examined by a senior cardiologist from the Yale Center for Outcomes Research and Evaluation. The review aimed at determining the concordance between the abstracted results of AMI types and the first available electrocardiogram (ECG) or ECG description in medical records. For the ECG review, we considered left bundle branch block (LBBB) as a STEMI equivalent. Our review showed that there was a 94.7% concordance in the selected cases. Details of the results are shown in the following table.

| **Review Results of AMI Types** | **N (%)** |
| --- | --- |
| Consistent | 284 (94.7%) |
| Consistent with ECG graph | 246 (82.0%) |
| Consistent with ECG description in records | 38 (12.7%) |
| Inconsistent | 12 (4.0%) |
| Unavailable (either ECG graph or ECG description in medical records) | 4 (1.3 %) |
