# Supplementary material for: Trends in Cardiac Biomarker Testing in China for Patients with Acute Myocardial Infarction, 2001 to 2011: China PEACE-Retrospective AMI Study
Source: PLoS One. 2015 Apr 20;10(4):e0122237. doi: 10.1371/journal.pone.0122237 (PMC4404305; doi:10.1371/journal.pone.0122237)
Supplement: S1 Protocol — (PDF) [file pone.0122237.s004.pdf]

Circulation
Cardiovascular Quality and Outcomes

American Heart Association®

The China Patient-Centered Evaluative Assessment of Cardiac Events (China PEACE) Retrospective Study of Acute Myocardial Infarction: Study Design

Kumar Dharmarajan, Jing Li, Xi Li, Zhenqiu Lin, Harlan M. Krumholz and Lixin Jiang
for the China PEACE Collaborative Group

Circ Cardiovasc Qual Outcomes. 2013;6:732-740; originally published online November 12, 2013;
doi: 10.1161/CIRCOUTCOMES.113.000441
Circulation: Cardiovascular Quality and Outcomes is published by the American Heart Association, 7272 Greenville Avenue, Dallas, TX 75231
Copyright © 2013 American Heart Association, Inc. All rights reserved.
Print ISSN: 1941-7705. Online ISSN: 1941-7713

The online version of this article, along with updated information and services, is located on the World Wide Web at:
http://circoutcomes.ahajournals.org/content/6/6/732

Permissions: Requests for permissions to reproduce figures, tables, or portions of articles originally published in *Circulation: Cardiovascular Quality and Outcomes* can be obtained via RightsLink, a service of the Copyright Clearance Center, not the Editorial Office. Once the online version of the published article for which permission is being requested is located, click Request Permissions in the middle column of the Web page under Services. Further information about this process is available in the Permissions and Rights Question and Answer document.

Reprints: Information about reprints can be found online at:
http://www.lww.com/reprints

Subscriptions: Information about subscribing to *Circulation: Cardiovascular Quality and Outcomes* is online at:
http://circoutcomes.ahajournals.org//subscriptions/

Downloaded from http://circoutcomes.ahajournals.org/ at Yale University on November 25, 2013

The China Patient-Centered Evaluative Assessment of Cardiac Events (China PEACE) Retrospective Study of Acute Myocardial Infarction: Study Design

Kumar Dharmarajan, MD, MBA*; Jing Li, MD, PhD*; Xi Li, MD, PhD; Zhenqiu Lin, PhD; Harlan M. Krumholz, MD, SM†; Lixin Jiang, MD, PhD†; for the China PEACE Collaborative Group

Background**—**Cardiovascular diseases are rising as a cause of death and disability in China. To improve outcomes for patients with these conditions, the Chinese government, academic researchers, clinicians, and >200 hospitals have created China Patient-Centered Evaluative Assessment of Cardiac Events (China PEACE), a national network for research and performance improvement. The first study from China PEACE, the Retrospective Study of Acute Myocardial Infarction (China PEACE-Retrospective AMI Study), is designed to promote improvements in acute myocardial infarction (AMI) quality of care by generating knowledge about the characteristics, treatments, and outcomes of patients hospitalized with AMI across a representative sample of Chinese hospitals during the past decade.

Methods and Results**—**The China PEACE-Retrospective AMI Study will examine >18 000 patient records from 162 hospitals identified using a 2-stage cluster sampling design within economic–geographic regions. Records were chosen from 2001, 2006, and 2011 to identify temporal trends. Data quality will be monitored by a central coordinating center and will, in particular, address case ascertainment, data abstraction, and data management. Analyses will examine patient characteristics, diagnostic testing patterns, in-hospital treatments, in-hospital outcomes, and variation in results by time and site of care. In addition to publications, data will be shared with participating hospitals and the Chinese government to develop strategies to promote quality improvement.

Conclusions**—**The China PEACE-Retrospective AMI Study is the first to leverage the China PEACE platform to better understand AMI across representative sites of care and during the past decade in China. The China PEACE collaboration among government, academicians, clinicians, and hospitals is poised to translate research about trends and patterns of AMI practices and outcomes into improved care for patients.

Clinical Trial Registration**—**URL: http://www.clinicaltrials.gov. Unique identifier: NCT01624883.

(*Circ Cardiovasc Qual Outcomes*. 2013;6:732-740.)

Key Words: epidemiology ■ morbidity ■ mortality ■ myocardial infarction

Ischemic heart disease has significant public health importance in China.[1,2] Unlike in the United States,[3,4] age-standardized ischemic heart disease incidence is rising in China.[5–10] Reasons for this trend include the increasing prevalence of traditional risk factors for atherosclerosis,[6,7,10–12] including hypertension,[6,7] hyperlipidemia,[7,13] diabetes mellitus,[7,13] obesity,[13–16] and inadequate physical activity[6,7] in the presence of significant exposure to cigarette smoke[7,17,18] and air pollution.[19] Population aging is further contributing to rising ischemic heart disease incidence.[10]

To improve outcomes for ischemic heart disease and other cardiovascular diseases in China, the China National Center for Cardiovascular Disease (NCCD), the Yale-New Haven Hospital Center for Outcomes Research and Evaluation, the Chinese

Received June 26, 2013; accepted September 17, 2013.

From The China PEACE Collaborative Group: State Key Laboratory of Cardiovascular Disease, China Oxford Centre for International Health Research, Fuwai Hospital, National Center for Cardiovascular Diseases, Chinese Academy of Medical Sciences and Peking Union Medical College, Beijing, People's Republic of China; and The Center for Outcomes Research and Evaluation, Yale-New Haven Hospital, New Haven, CT; Center for Outcomes Research and Evaluation, Yale-New Haven Hospital, New Haven, CT (K.D., Z.L., H.M.K.); Division of Cardiology, Columbia University Medical Center, New York, NY (K.D.); State Key Laboratory of Cardiovascular Disease, China Oxford Centre for International Health Research, Fuwai Hospital, National Center for Cardiovascular Diseases, Chinese Academy of Medical Sciences and Peking Union Medical College, Beijing, People's Republic of China (J.L., X.L., L.J.); Section of Cardiovascular Medicine and the Robert Wood Johnson Clinical Scholars Program, Department of Internal Medicine, Yale University School of Medicine, New Haven, CT (H.M.K.); and Department of Health Policy and Management, Yale School of Public Health, New Haven, CT (H.M.K.).

*Dr Dharmarajan and Dr J. Li are joint first authors.

†Drs Krumholz and Jiang are joint senior authors.

This manuscript was handled independently by Kirsten Bibbins-Domingo, MD, PhD, MS, as a Guest Editor. The Editors had no role in the evaluation of the manuscript or in the decision about its acceptance.

The online-only Data Supplement is available at http://circoutcomes.ahajournals.org/lookup/suppl/doi:10.1161/CIRCOUTCOMES.113.000441/-/DC1.

Correspondence to Lixin Jiang, State Key Laboratory of Cardiovascular Disease, China Oxford Center for International Health Research, Fuwai Hospital, National Center for Cardiovascular Diseases, Chinese Academy of Medical Sciences and Peking Union Medical College, 167 Beilishi Rd, Beijing 100037, People's Republic of China. E-mail lixin.jiang@fwoxford.org

© 2013 American Heart Association, Inc.

***Circ Cardiovasc Qual Outcomes* is available at http://circoutcomes.ahajournals.org** **DOI: 10.1161/CIRCOUTCOMES.113.000441**

Downloaded from http://circoutcomes.ahajournals.org/ at Yale University on November 25, 2013

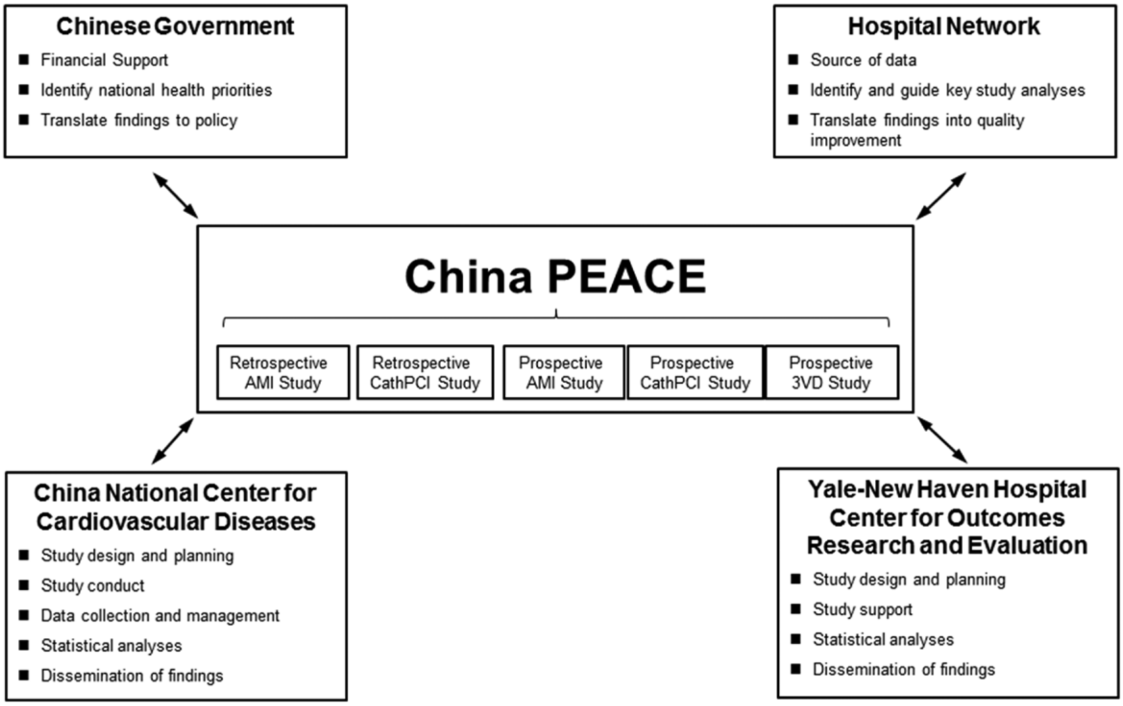

Figure 1. The China Patient-Centered Evaluative Assessment of Cardiac Events (PEACE) initiative. Key partners include the Chinese government, collaborating hospitals, the China National Center for Cardiovascular Disease, and the Yale-New Haven Hospital Center for Outcomes Research and Evaluation. The China PEACE-Retrospective AMI Study is 1 of 5 initial studies from the China PEACE initiative. The topic areas for these 5 projects concern acute myocardial infarction, coronary catheterization/percutaneous coronary intervention, and multi-vessel coronary artery disease. Future studies will focus on cerebrovascular disease and other cardiovascular conditions. 3VD indicates revascularization in patients with triple-vessel disease; AMI, acute myocardial infarction; and PCI, percutaneous coronary intervention.

government, and >200 Chinese hospitals have collaborated to create a national network for research and performance improvement. This platform, entitled China PEACE (Patient-Centered Evaluative Assessment of Cardiac Events), will permit rapid bidirectional flow of information between partnering hospitals and the coordinating center at the NCCD (Figure 1). With funding from the Chinese government, China PEACE will elucidate the clinical epidemiology of cardiovascular disease treatment and its associated outcomes across patients, hospitals, regions, and time. China PEACE, envisioned as a combined research and quality improvement initiative, will disseminate findings of the greatest relevance to participating hospitals and the Chinese government in a manner suitable for the evaluation of care and the development of projects to improve clinical quality and patient outcomes. Through the use of strategies most often used by clinical trials, data quality will be ensured by rigorous monitoring by the central coordinating center at the stages of case ascertainment, data abstraction, and data management. The collaborative research and performance improvement network created by China PEACE will ultimately be leveraged to improve patient outcomes for a broad range of conditions and may be a model for research and quality improvement in other international settings.

The first study from the China PEACE collaboration is the Retrospective Study of Acute Myocardial Infarction (China PEACE-Retrospective AMI Study). Previous research on the characteristics, treatments, and outcomes for patients hospitalized with acute myocardial infarction (AMI) in China has contributed important knowledge but has been largely limited to studies from tertiary care centers in urban regions,[20–22] major metropolitan areas,[23–25] a small subset of provinces,[26,27] or to patients from clinical trial databases,[28] all of whom may not be representative of typical patients. Research involving a larger and more diverse distribution of study sites has contributed to our understanding of the use of evidence-based therapies after AMI,[29] including differences in treatment between secondary and tertiary hospitals[30] as well as in-hospital complications such as bleeding[31] and recurrent angina,[32] but it has not involved a large, nationally representative study population and has not examined temporal trends in AMI treatment and outcomes. These trends over time, in particular, may reflect major changes in the Chinese healthcare system that have occurred in the past decade such as the expansion of health insurance from 30% to 90% of the population between 2001 and 2011,[33] greatly increased resources for rural healthcare beginning in 2003,[34] and further health reforms starting in 2009.[35] Data relating these health reforms to changing healthcare practices are limited. To address these gaps in knowledge, the China PEACE-Retrospective AMI Study will identify trends using a nationally representative sample of >18 000 patient records from 162 randomly selected hospitals for 2001, 2006, and 2011. The hospitals represent diverse geographic regions and include institutions with a range of cardiovascular facilities. Thus, this is the first truly national assessment of practice patterns and outcomes for AMI performed in China.

The China PEACE-Retrospective AMI Study broadly aims to promote improvements in AMI quality of care by generating knowledge about the characteristics, treatments, and outcomes of patients hospitalized with AMI across a representative

Downloaded from http://circoutcomes.ahajournals.org/ at Yale University on November 25, 2013

sample of Chinese hospitals during the past decade. The study is largely descriptive, and rather than test a specific hypothesis, it seeks to characterize current AMI care and associated patient outcomes to provide a foundation for future quality improvement and research. We do anticipate that there will be marked variation in practice and outcomes, demographic and geographic disparities, and ample opportunities for improvements. The specific aims of the China PEACE-Retrospective AMI Study are to (1) describe the characteristics of patients hospitalized with AMI in China, including their clinical and demographic attributes such as occupation and insurance status; (2) characterize patterns of in-hospital treatment, including the use of traditional Chinese medicines; (3) describe mortality rates and other in-hospital outcomes, including the development of heart failure and complications of treatment; (4) determine trends over time in patient characteristics, treatments, and outcomes; (5) develop and test prognostic scores to stratify risk; (6) compare treatment across regions and hospitals and determine whether differences in treatment patterns by setting may be associated with differences in outcomes; (7) examine the alignment of diagnostic testing and treatment strategies with quality measures; (8) compare differences in patient characteristics, treatment approaches, and outcomes between China and other countries; (9) determine the quality of documentation within the medical record; and (10) collaborate with participating hospitals and the Chinese government to disseminate study findings to improve quality of care and outcomes.

This article describes study methodology, abstracted data elements, analytic plan, and preliminary findings of the China PEACE-Retrospective AMI Study. Findings will identify opportunities for quality improvement and guide the development of strategies and tools to improve outcomes for AMI in China.

Methods

Design Overview

The China PEACE-Retrospective AMI Study will examine >18 000 hospitalizations for AMI from a nationally representative network of Chinese hospitals during 2001, 2006, and 2011. The study includes hospitalizations with a principal discharge diagnosis of AMI (International Classification of Diseases, Ninth Revision, Clinical Modification codes 410.xx or International Classification of Diseases, Tenth Revision, Clinical Modification codes I21.xx), including ST-segment–elevation myocardial infarction and non–ST-segment–elevation myocardial infarction. We did not include hospitalizations with a principal discharge diagnosis of unstable angina.

To study 10-year trends in patient characteristics, treatment patterns, and outcomes nationally and within regions of different socioeconomic development, we drew a random, representative sample of patient discharges for each year. We intentionally drew a larger sample for 2011 to study differences in treatment patterns and outcomes across hospitals.

The central ethics committee at the China NCCD approved the PEACE-Retrospective AMI Study. All collaborating hospitals accepted the central ethics approval except for 5 hospitals, which obtained local approval by internal ethics committees. The study is listed at www.clinicaltrials.gov (NCT01624883).

The Chinese government, which provided financial support for the study, had no role in the design or conduct of the study; in the collection, management, analysis, and interpretation of the data; or in the preparation or approval of the article.

Sampling Design

We intended study hospitals to reflect diverse sites of care in China. As hospital volumes and clinical capacities differ between urban and rural areas, as well as among the 3 official economic–geographic regions of Mainland China, we separately identified hospitals in 5 strata: Eastern-rural, Central-rural, Western-rural, Eastern-urban, and Central/Western-urban regions. We considered an area urban if it is part of a downtown or suburban area within a direct-controlled municipality (Beijing, Tianjin, Shanghai, Chongqing) or 1 of 283 prefectural-level cities. We considered surrounding county-level regions, including counties and county-level cities, to be rural. Within this framework, Mainland China is composed of 287 urban regions and 2010 rural regions. We considered Central and Western-urban regions together given their similar per capita income and health services capacity.[36]

We identified cases for study inclusion using a stratified 2-stage cluster sampling design (Figure 2). In the first stage, we identified hospitals using a simple random sampling procedure within each of the 5 study strata. In the 3 rural strata, the sampling framework consisted of the central hospital in each of the predefined rural regions (2010 central hospitals in 2010 rural regions). Within each rural region, the central hospital is the largest general hospital with the greatest clinical capacity for treating acute illness, including AMI. In each of the 2 urban strata, the sampling framework consisted of the highest-level hospitals in each of the predefined urban regions (833 hospitals in 287 urban regions). Hospital level is officially defined by the Chinese government based on clinical resource capacity.[37] For example, secondary hospitals have ≥100 inpatient beds and the capacity to provide acute medical care and preventive care services to populations of ≥100 000, whereas tertiary hospitals are large referral centers in provincial capitals and major cities.[29] We excluded military hospitals, prison hospitals, specialized hospitals without a cardiovascular disease division, and traditional Chinese medicine hospitals. We decided to select representative hospitals from 2011 to reflect current practices and trace this hospital cohort backward to 2006 and 2001 to describe temporal trends. As the hospital number has grown by ≈18% during the past decade,[36,38] the study cohort should be most representative of national treatment patterns and outcomes in 2011.

In the second stage, we drew cases based on the local hospital database for patients with AMI at each sampled hospital using systematic random sampling procedures. In each of the 5 study strata, we determined the sample size required to achieve a 2% precision for describing the primary outcome, in-hospital mortality, which we had estimated to be ≈9% in urban hospitals and 7% in rural county-level hospitals.[5] To achieve a precision of 2% with an α of 0.05 in each of the 3 rural strata, assuming an intraclass correlation of 0.02 and design effect of 1.8, we would need to sample 1150 medical records among hospitals with an average cluster size of 40. Analogously, to achieve a precision of 2% with an α of 0.05 in each of the 2 urban strata, assuming an intraclass correlation of 0.02 and design effect of 2.2, we would need to sample 1750 medical records among hospitals with an average cluster size of 60. These cluster sizes in rural and urban settings seemed reasonable based on our previous survey of treatment for acute coronary syndromes at >1000 hospitals in 2010, which demonstrated that the median volume of hospitalization for AMI was ≈180 annual cases in urban hospitals and 95 annual cases in rural county-level hospitals. Assuming a participation rate of 85% among selected hospitals, we approached 35 hospitals for participation in each stratum for a total of 175 hospitals (70 urban and 105 rural). We doubled cluster sizes for 2011 to improve precision in the description of hospital-level treatment patterns and outcomes. Consequently, the total expected sample volume with the above assumptions was ≈6950 cases in 2001, 6950 cases in 2006, and 13 900 cases in 2011. A more detailed description of the sampling strategy used in the PEACE-Retrospective AMI Study is provided in the supplemental material.

Data Collection

We trained staff at participating hospitals to identify all hospitalizations for AMI from their respective local hospital databases for the years 2001, 2006, and 2011. After we sampled cases at each hospital, we assigned each case a unique study ID. We then required local investigators to gather the original record, scan it, and transmit the scanned copy to the coordinating center. To facilitate this process, the coordinating center provided each study site with a high-speed scanner. To verify compliance with the case finding strategy, research

Downloaded from http://circoutcomes.ahajournals.org/ at Yale University on November 25, 2013

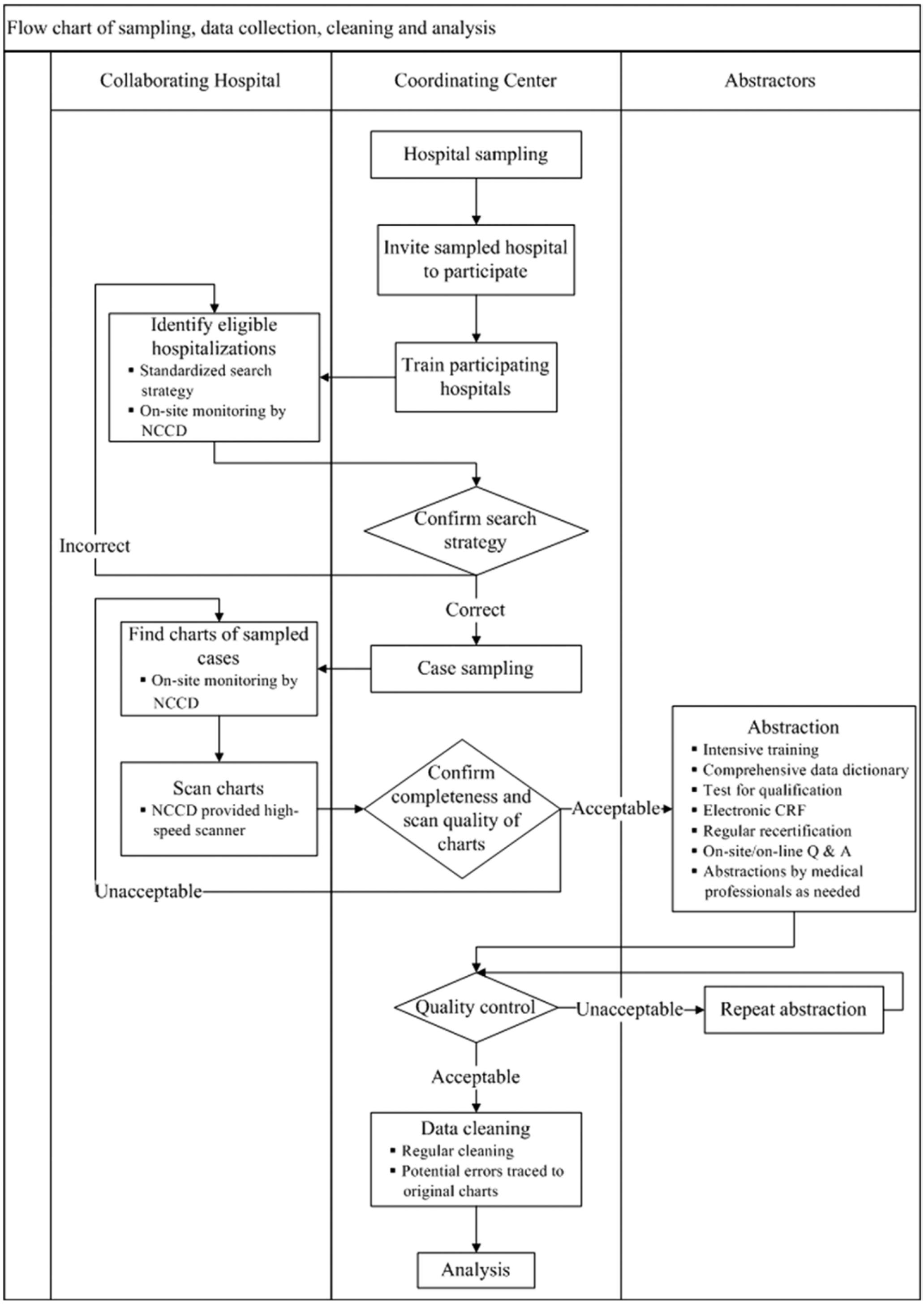

Figure 2. China Patient-Centered Evaluative Assessment of Cardiac Events (PEACE) Retrospective Study of Acute Myocardial Infarction flow chart and associated quality assurance strategies. Flow chart should be read from top to bottom. CRF indicates case report form; NCCD, China National Center for Cardiovascular Diseases; and Q&A, questions and answers.

staff from the study coordinating center visited 46 study sites to repeat the case finding process, confirm that the list of hospitalizations with AMI was complete, and assist in acquiring the sampled cases (Figure 2). These 46 sites provided ≈50% of sampled cases for the China PEACE-Retrospective AMI Study.

After identifying all medical records for sampled cases, participating hospitals copied and transmitted the records to the NCCD after deidentification. Research staff ensured the completeness and quality with which each medical record was scanned; incomplete or poorly scanned records were rescanned and retransmitted (Figure 2). We instructed study sites to include all parts of the medical record, including the face sheet, admission note, daily progress notes, procedure notes, medication administration record, diagnostic procedure reports, laboratory test results, physician orders, nursing notes, and discharge summary. These subdivisions of the medical record are routinely present throughout China.

The China PEACE-Retrospective AMI Study has adhered to rigorous standards for abstraction. Before initiating chart review, each

Downloaded from http://circoutcomes.ahajournals.org/ at Yale University on November 25, 2013

abstractor received 2 weeks of training that included an introduction to the study and instruction about coronary heart disease and its subtypes, component parts of the inpatient medical record including specialized sections such as catheterization reports, and the China PEACE-Retrospective AMI Study data dictionary. We provided all material, including the data dictionary, in Chinese. After training, we certified trainees who were able to abstract 10 sample medical records with >98% accuracy (online-only Data Supplement).

The China PEACE-Retrospective AMI Study used several strategies to improve the accuracy of abstraction. Inexperienced abstractors began with exclusively typewritten rather than hand written medical records. In addition, we randomly audited ≈5% of the abstracted records. If the records were not abstracted with 98% accuracy, all medical records in the audited batch were considered unqualified and were rereviewed by a different abstractor. We used abstractors with formal medical training to identify data elements requiring more advanced medical knowledge for recognition, such as the presence of comorbidities, evidence of pulmonary edema on hospital presentation, and the development of postprocedural complications, such as bleeding or arrhythmia. A physician was always present in the room with abstractors or was available online to answer questions as they arose. As problems were identified, we updated the data dictionary and web-based data management program built for the China PEACE-Retrospective AMI Study into which data were directly entered. We have additionally customized this program to expedite the identification of medications that may have multiple trade names. Finally, we assigned medical records belonging to the same hospital and year to a broad group of reviewers to avoid potential residual disparities in quality among abstractors (online-only Data Supplement).

Data Management

We have treated all data as protected health information and have securely stored it in an encrypted and password-protected database at the coordinating center. We have securely stored paper charts in locked rooms.

We perform ongoing data cleaning systematically. Data managers regularly query data for invalid and illogical values, as well as for duplicate record entries. They identify potential invalid values by searching for outliers in continuous data distributions. Records with identical study identification numbers, hospital identification numbers, medical record identification numbers, and dates of discharge trigger a search for duplicate records. Once a potential error is found, data managers trace and review the relevant records to resolve the issue.

Data Elements

We examined both the English language and the Chinese literature for relevant studies to create a candidate list of potential data elements. Where possible, we included elements particular to the Chinese context such as the use of traditional Chinese medicines. We supplemented these elements with variables used in the Get with the Guidelines ACTION (Acute Coronary Treatment and Intervention Outcomes Network) Registry of the National Cardiovascular Data Registry (NCDR) and the Variation in Recovery: Role of Gender on Outcomes of Young Acute Myocardial Infarction Patients (VIRGO) study. ACTION is an outcome-based registry and quality improvement program that focuses on patients hospitalized with ST-segment–elevation myocardial infarction and non–ST-segment–elevation myocardial infarction.[39] VIRGO is a large, observational study of the presentation, treatment, and outcomes of young women and men with AMI.[40] The use of standardized elements from ACTION and VIRGO permit cross-country comparisons (Table 1). We performed pilot testing of the case report form on >500 medical records from study sites across China to enhance clarity of wording and further guide variable selection. We present data relevant to performance measures during the first 24 hours of hospitalization and at hospital discharge in Table 2. Where possible, we collected data that would allow us to construct the core quality measures used and reported by the Centers for Medicare & Medicaid Services in the United States. Each participating hospital has also completed a survey, modeled on the annual survey of hospitals performed by the American Hospital Association, of its major structural and organizational characteristics.[41] Key variables assessed include bed size, annual volume of AMI, teaching status, and capacity to perform invasive revascularization procedures. The patient case report form and associated data dictionary are available in the online-only Data Supplement.

Statistical Analyses

We will report summary statistics for patient characteristics, use of diagnostic tests, treatments received, and in-hospital outcomes including complications of care across study sites. Weighting will reflect the reciprocal of sampling probability. For each aim, we will use standard parametric and nonparametric techniques for observational data, including *t* tests, χ^2 tests, Wilcoxon rank-sum tests, and generalized linear models. Because patient characteristics, treatments, and outcomes may be correlated within study sites, analyses will account for the effect of clustering. To examine and adjust for differences between comparison groups, we will use linear, logistic, Cox proportional hazard, and Poisson models with a generalized estimating

Table 1. China PEACE-Retrospective Study of Acute Myocardial Infarction Data Elements

Category	Example Elements
Patient demographics	Age, sex, ethnicity, postal code, occupation, and insurance status
Medical history	Diabetes mellitus, hypertension, hyperlipidemia, vascular disease, and prior revascularization
Initial cardiac status	Heart rate, blood pressure, Killip class, heart failure, and cardiac arrest
Laboratory values	Troponin, CK, CK-MB, BNP, sodium, BUN, creatinine, WBC count, and hemoglobin
Medications including dose	Antithrombotic therapy, β-blocker, ACE inhibitor/ARB, statin, and traditional Chinese medicines
Revascularization	Fibrinolysis, PCI (access, anatomy, stent number, stent type, contrast dose, closure device), and CABG surgery
Diagnostic procedures	Echocardiogram, CT angiogram, stress testing, and chest radiograph
Outcomes including in-hospital complications	Death, heart failure, shock, arrhythmia, stroke, bleeding, transfusion, and infection

ACE indicates angiotensin-converting enzyme; ARB, angiotensin receptor blocker; BNP, brain natriuretic peptide; BUN, blood urea nitrogen; CABG, coronary artery bypass graft; CK, creatine kinase; CK-MB, creatine kinase-MB fraction; CT, computed tomography; PCI, percutaneous coronary intervention; PEACE, Patient-Centered Evaluative Assessment of Cardiac Events; and WBC, white blood cell.

Downloaded from http://circoutcomes.ahajournals.org/ at Yale University on November 25, 2013

Table 2. China PEACE-Retrospective Study of Acute Myocardial Infarction Performance Measures

First 24 h	Discharge
Aspirin	Aspirin
Heparin	Clopidogrel
Evaluation of left ventricular function	β-blocker
Time to primary PCI	ACE inhibitor or ARB for LV systolic dysfunction
Time to fibrinolysis	Statin
Overall reperfusion therapy	Smoking cessation counseling
	Cardiac rehabilitation referral

ACE indicates angiotensin-converting enzyme; ARB, angiotensin receptor blocker; PEACE, Patient-Centered Evaluative Assessment of Cardiac Events; LV, left ventricle; and PCI, percutaneous coronary intervention.

equation approach and hierarchical models, where appropriate. We will develop models to stratify patients according to their risk of adverse outcomes. We will assess the relationship of candidate variables to in-hospital outcomes using appropriate statistical techniques for the dependent variable. We will further refine the list of candidate variables based on their clinical relevance.

Progress to Date

As of May 2013, 162 hospitals have agreed to participate in the China PEACE-Retrospective AMI Study (Figure 3). Of the 13 that did not participate, 7 did not have admissions for AMI, and 6 declined participation. Examination of patient databases from participating hospitals yielded 31 601 hospitalizations for AMI (3859 in 2001, 8863 in 2006, and 18 879 in 2011). Of these, we sampled 18 631 for the China PEACE-Retrospective AMI Study (2801 in 2001, 5199 in 2006, and 10 631 in 2011). Of these 18 631 sampled hospitalizations, we acquired medical records for 18 110 (97.2%) and began data abstraction in August 2012. Medical records from 95% (154) of study sites contained all expected sections and represent 89% of all hospitalizations in the China PEACE-Retrospective AMI Study. In the remaining 8 study sites, we did not have access to daily progress notes because of local administrative policies of hospital archives departments. However, these records were considered adequate for inclusion in the study. The use of electronic medical records increased with time: 0% of hospitals used electronic medical records in 2001, 7% of hospitals used electronic medical records in 2006, and 46% of hospitals used electronic medical records in 2011. We will code hospital-level variables for the presence of daily progress notes and electronic medical records to better understand whether these factors introduce bias into study results.

To verify the accuracy of principal discharge diagnoses, we randomly selected 300 medical records and examined concordance between principal discharge diagnosis and electrocardiographic findings consistent with the subtype of AMI (ST-segment–elevation myocardial infarction versus non–ST-segment–elevation myocardial infarction). We found concordance in 95% of cases.

Discussion

Akin to the Cooperative Cardiovascular Project in the United States,[42] China PEACE provides a platform through which government, healthcare providers, and research organizations can translate knowledge of the clinical epidemiology of cardiovascular disease into improved care for patients. In the United States, collaboration between the Healthcare Financing Administration, hospitals, and academic researchers demonstrated frequent underuse of evidence-based therapies for AMI.[43] Rapid feedback of these findings to hospitals resulted in significant improvement in performance on all studied quality indicators and reduced mortality.[44] China PEACE has similar potential to serve as a foundational project that evaluates and, if necessary, elevates cardiovascular care more generally within China. As with the United States in the early 1990s, China is turning increased attention to the treatment of patients hospitalized with acute cardiovascular conditions[45] to improve outcomes across diverse patient populations and sites of care. China PEACE will leverage the unique resources of the Chinese government, a diverse hospital network, and an international research team to translate study findings into action for the benefit of patients.

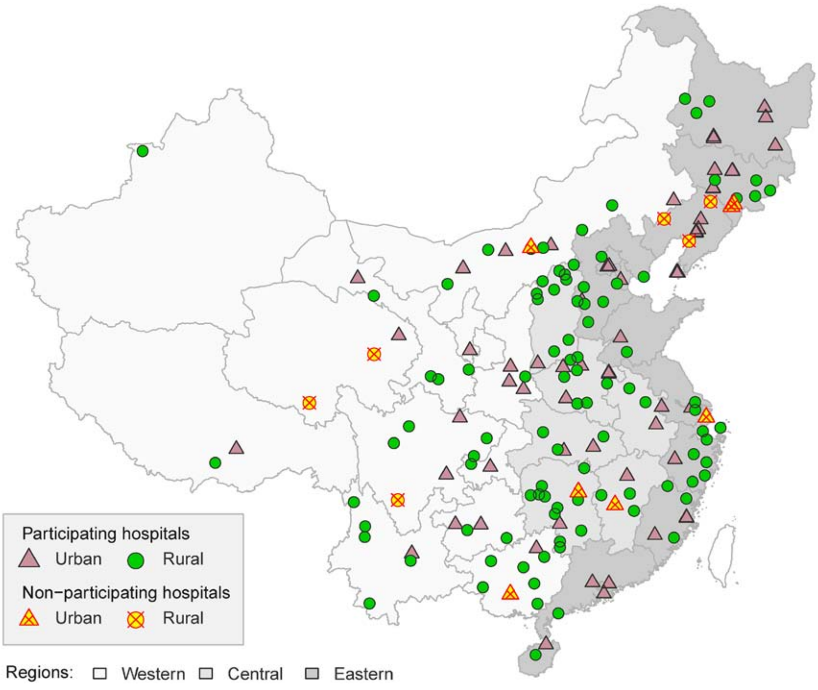

Figure 3. Geographic distribution of participating hospitals in the China Patient-Centered Evaluative Assessment of Cardiac Events (PEACE) Retrospective Study of Acute Myocardial Infarction (AMI). Of 175 sampled hospitals, 13 were unable or unwilling to participate, and 162 provided cases for the China PEACE-Retrospective AMI Study.

Downloaded from http://circoutcomes.ahajournals.org/ at Yale University on November 25, 2013

The partnership among government, hospitals, and researchers is a critical source of strength for China PEACE. For example, clinical champions have informed the content of case report forms, thereby increasing the likelihood that study questions and findings are relevant at the front lines of care. Continuing conversations with hospitals will help align planned analyses to the areas of greatest concern and uncertainty for providers. The NCCD will tailor performance feedback reports to the information needs and clinical goals of each hospital. For example, the NCCD will be able to benchmark site-specific data on patient characteristics, receipt of evidence-based treatment, and outcomes to nearby hospitals, hospitals with similar case mix, top performing hospitals, the full spectrum of Chinese hospitals, or international institutions. In addition, the research teams will serve as hubs to ensure the rapid transfer of data, including information on best practices, between hospitals, and the Chinese government. The direct ties to government will increase the likelihood that study findings influence policy pertinent to AMI and cardiovascular care. Such an arrangement may also facilitate the creation of clinical tools to improve care, such as standing order templates, risk stratification algorithms, and dosing pocket cards.

China PEACE is further distinguished by its use of data quality control strategies, which are much more common in multinational clinical trials than in large retrospective studies. In particular, the China PEACE-Retrospective AMI Study has devoted significant attention to data quality at the stages of case ascertainment, data abstraction, and data management. For example, research staff rigorously monitored study sites to identify all hospitalizations for AMI from census databases. Staff also ensured that medical records for sampled cases were physically found, properly copied, and transmitted in full whenever possible. In addition, the China PEACE-Retrospective AMI Study provided central training for abstractors and required rigorous standards for both initial certification and recertification. Medical records from abstractors who did not achieve recertification requirements were reabstracted by a second reviewer. In developing these systems, China PEACE is also positioned for future studies.

The China PEACE-Retrospective AMI Study has several additional strengths. It contains the largest representative sample of hospitalizations for AMI in China and therefore includes patients from diverse geographic regions and institutions with widely varying resource capacities. Its inclusion of data from 2001, 2006, and 2011 will permit the assessment of trends in patient characteristics, care patterns, and outcomes. Information will be available on topics that have not been well studied (eg, the use of traditional Chinese medicines after AMI). Cross-country comparisons will be possible given the alignment of key data elements with the NCDR ACTION Registry and VIRGO. Finally, further targeted examination of additional data elements not included in the initial case report forms can be performed as novel questions arise, as the NCCD will maintain a physical copy of all charts after the initial abstraction.

The China PEACE-Retrospective AMI Study has some limitations. Study findings depend on the accuracy and completeness of the abstracted medical records. However, this limitation pertains to all retrospective chart reviews, including those performed by the Cooperative Cardiovascular Project and registries. Lack of completeness may signal problems with documentation that are important to note, as detailed and reliable record keeping is needed to measure key processes and outcomes in quality improvement. Therefore, the assessment of the quality of the medical record can be an important contribution of this effort. The China PEACE-Retrospective AMI Study is also restricted to measuring in-hospital outcomes, as we are unable to link patient-level data to a national registry of deaths. However, the length of stay in Chinese hospitals, extended compared with that of many Western countries,[5,36,46,47] should permit more robust estimates of short-term complications including death. Next, we are unable to report on hospital costs and cost-effectiveness related to care for AMI, as well as patient out-of-pocket costs, as this information is not available in the standard medical record, which only contains information on the total charge for hospitalization. Finally, study precision will be slightly lower than anticipated, as we collected data from ≈18 000 medical records rather than the anticipated 28 000. This discrepancy resulted from the substantially lower volume of hospitalizations for AMI than had been anticipated for the years 2001 and 2006, in particular. Our estimates, which were based on 2010 survey data, did not fully account for the marked increase in AMI hospitalizations that we identified between 2001 and 2011. However, the smaller sample size than expected should not substantively affect the precision with which major outcomes such as mortality are described, as expected strata-level precision for mortality decreased by no more than 2% in rural areas and no more than 1% in urban areas (online-only Data Supplement).

China PEACE will ensure transparency in research design, performance, and reporting. Although the Chinese government has funded China PEACE, it has no role in conduct of the study. We will perform all analyses in academic research units at the China NCCD and the Yale-New Haven Hospital Center for Outcomes Research and Evaluation. Government approval of articles will not be required before publication.

China PEACE is intended to spur similar collaborations among government, healthcare institutions, and academic researchers to better understand cardiovascular disease incidence, treatment, and outcomes. Partnerships among local hospitals at the front line of care, domestic research organizations with local expertise and previous track records of success, international experts in research design, and policymakers intent on improving population health can identify important targets for study, build novel research networks, and generate tools useful in improving health outcomes. These partnerships can support a research and health improvement infrastructure that is unconstrained by particular diseases or conditions and is capable of facilitating clinical trials and performance improvement activities. In the future we will seek to involve patients and caregivers in the research team. The China PEACE-Retrospective AMI Study is the first of many projects that can leverage the China PEACE platform to elucidate the patterns of care and outcomes of cardiovascular disease across patients, hospitals, and regions within China.

Acknowledgments

We appreciate the guidance provided by Prof Liming Li (Chinese Academy of Medical Sciences), Prof Zhengming Chen (University of Oxford), Dr Jun Lv, and Dr Qiushan Tao (Peking University) with

Downloaded from http://circoutcomes.ahajournals.org/ at Yale University on November 25, 2013

regard to study design. We appreciate the multiple contributions made by study teams at the China Oxford Centre for International Health Research and the Yale-New Haven Hospital Center for Outcomes Research and Evaluation in the realms of study design and operations. We are grateful for the support provided by the Chinese government.

Sources of Funding

This project was partly supported by grant 201202025 from the Ministry of Health of China. Dr Dharmarajan is supported by grant HL007854 from the National Heart, Lung, and Blood Institute; he is also supported as a Centers of Excellence Scholar in Geriatric Medicine at Yale by the John A. Hartford Foundation and the American Federation for Aging Research. Dr Krumholz is supported by grant U01 HL105270-03 (Center for Cardiovascular Outcomes Research at Yale University) from the National Heart, Lung, and Blood Institute. The sponsors had no role in the conduct of the study; in the collection, management, analysis, and interpretation of the data; or in the preparation or approval of the article.

Disclosures

None.

References

1. He J, Gu D, Wu X, Reynolds K, Duan X, Yao C, Wang J, Chen CS, Chen J, Wildman RP, Klag MJ, Whelton PK. Major causes of death among men and women in China. *N Engl J Med*. 2005;353:1124–1134.
2. Yang G, Wang Y, Zeng Y, Gao GF, Liang X, Zhou M, Wan X, Yu S, Jiang Y, Naghavi M, Vos T, Wang H, Lopez AD, Murray CJ. Rapid health transition in China, 1990-2010: findings from the Global Burden of Disease Study 2010. *Lancet*. 2013;381:1987–2015.
3. Yeh RW, Sidney S, Chandra M, Sorel M, Selby JV, Go AS. Population trends in the incidence and outcomes of acute myocardial infarction. *N Engl J Med*. 2010;362:2155–2165.
4. Go AS, Mozaffarian D, Roger VL, Benjamin EJ, Berry JD, Borden WB, Bravata DM, Dai S, Ford ES, Fox CS, Franco S, Fullerton HJ, Gillespie C, Hailpern SM, Heit JA, Howard VJ, Huffman MD, Kissela BM, Kittner SJ, Lackland DT, Lichtman JH, Lisabeth LD, Magid D, Marcus GM, Marelli A, Matchar DB, McGuire DK, Mohler ER, Moy CS, Mussolino ME, Nichol G, Paynter NP, Schreiner PJ, Sorlie PD, Stein J, Turan TN, Virani SS, Wong ND, Woo D, Turner MB; American Heart Association Statistics Committee and Stroke Statistics Subcommittee. Heart disease and stroke statistics–2013 update: a report from the American Heart Association. *Circulation*. 2013;127:e6–e245.
5. Tunstall-Pedoe H, Kuulasmaa K, Mähönen M, Tolonen H, Ruokokoski E, Amouyel P. Contribution of trends in survival and coronary-event rates to changes in coronary heart disease mortality: 10-year results from 37 WHO MONICA project populations: monitoring trends and determinants in cardiovascular disease. *Lancet*. 1999;353:1547–1557.
6. Yang G, Kong L, Zhao W, Wan X, Zhai Y, Chen LC, Koplan JP. Emergence of chronic non-communicable diseases in China. *Lancet*. 2008;372:1697–1705.
7. Hu SS, Kong LZ, Gao RL, Zhu ML, Wang W, Wang YJ, Wu ZS, Chen WW, Liu MB; Editorial Board. Outline of the report on cardiovascular disease in China, 2010. *Biomed Environ Sci*. 2012;25:251–256.
8. Wu Z, Yao C, Zhao D, Wu G, Wang W, Liu J, Zeng Z, Wu Y. Sino-MONICA project: a collaborative study on trends and determinants in cardiovascular diseases in China, Part i: morbidity and mortality monitoring. *Circulation*. 2001;103:462–468.
9. Zhao D, Liu J, Wang W, Zeng Z, Cheng J, Liu J, Sun J, Wu Z. Epidemiological transition of stroke in China: twenty-one-year observational study from the Sino-MONICA-Beijing Project. *Stroke*. 2008;39:1668–1674.
10. Moran A, Gu D, Zhao D, Coxson P, Wang YC, Chen CS, Liu J, Cheng J, Bibbins-Domingo K, Shen YM, He J, Goldman L. Future cardiovascular disease in China: Markov model and risk factor scenario projections from the coronary heart disease policy model-China. *Circ Cardiovasc Qual Outcomes*. 2010;3:243–252.
11. Zhang XH, Lu ZL, Liu L. Coronary heart disease in China. *Heart*. 2008;94:1126–1131.
12. Yong H, Foody J, Linong J, Dong Z, Wang Y, Ma L, Meng HJ, Shiff S, Dayi H. A systematic literature review of risk factors for stroke in China. *Cardiol Rev*. 2013;21:77–93.
13. Critchley J, Liu J, Zhao D, Wei W, Capewell S. Explaining the increase in coronary heart disease mortality in Beijing between 1984 and 1999. *Circulation*. 2004;110:1236–1244.
14. Ma GS, Li YP, Wu YF, Zhai FY, Cui ZH, Hu XQ, Luan DC, Hu YH, Yang XG. The prevalence of body overweight and obesity and its changes among Chinese people during 1992 to 2002. *Zhonghua Yu Fang Yi Xue Za Zhi*. 2005;39:311–315.
15. Wu Y. Overweight and obesity in China. *BMJ*. 2006;333:362–363.
16. Wang H, Du S, Zhai F, Popkin BM. Trends in the distribution of body mass index among Chinese adults, aged 20-45 years (1989-2000). *Int J Obes (Lond)*. 2007;31:272–278.
17. Yang G, Fan L, Tan J, Qi G, Zhang Y, Samet JM, Taylor CE, Becker K, Xu J. Smoking in China: findings of the 1996 National Prevalence Survey. *JAMA*. 1999;282:1247–1253.
18. Gu D, Wu X, Reynolds K, Duan X, Xin X, Reynolds RF, Whelton PK, He J; InterASIA Collaborative Group. Cigarette smoking and exposure to environmental tobacco smoke in China: the international collaborative study of cardiovascular disease in Asia. *Am J Public Health*. 2004;94:1972–1976.
19. Chen R, Zhang Y, Yang C, Zhao Z, Xu X, Kan H. Acute Effect of Ambient Air Pollution on Stroke Mortality in the China Air Pollution and Health Effects Study. *Stroke*. 2013.
20. Yang XD, Zhao YS, Li YF, Guo XH. Medical comorbidities at admission is predictive for 30-day in-hospital mortality in patients with acute myocardial infarction: analysis of 5161 cases. *J Geriatr Cardiol*. 2011;8:31–34.
21. Zhang H, Sun S, Tong L, Li R, Cao XH, Zhang BH, Zhang LH, Huang JX, Ma CS. Effect of cigarette smoking on clinical outcomes of hospitalized Chinese male smokers with acute myocardial infarction. *Chin Med J (Engl)*. 2010;123:2807–2811.
22. Chu PH, Chiang CW, Cheng NJ, Ko YL, Chang CJ, Chen WJ, Kuo CT, Hsu TS, Lee YS. Gender differences in baseline variables, therapies and outcomes in Chinese patients with acute myocardial infarction. *Int J Cardiol*. 1998;65:75–80.
23. Wang SR, Liu HX, Zhao D, Lei Y, Wang W, Shang JJ, Fang YT, Shi ZX, Huang Y, Li QL; Beijing Collaborative Study Group on Therapies Status of Acute Myocardial Infarction. Study on the therapeutic status of 1242 hospitalized acute myocardial infarction patients in Beijing. *Zhonghua Liu Xing Bing Xue Za Zhi*. 2006;27:991–995.
24. Zhang SY, Hu DY, Sun YH, Yang JG. Current management of patients with ST elevation myocardial infarction in Metropolitan Beijing, China. *Clin Invest Med*. 2008;31:E189–E197.
25. Song L, Hu DY, Yang JG, Sun YH, Liu SS, Li C, Feng Q, Wu D. Factors leading to delay in decision to seek treatment in patients with acute myocardial infarction in Beijing. *Zhonghua Nei Ke Za Zhi*. 2008;47:284–287.
26. Yu LT, Zhu J, Mister R, Zhang Y, Li JD, Wang DL, Liu LS, Flather M. The Chinese registry on reperfusion strategies and outcomes in ST-elevation myocardial infarction. *Zhonghua Xin Xue Guan Bing Za Zhi*. 2006;34:593–597.
27. Zhang B, Jiang DM, Zhou XC, Liu J, Zhang W, Sun YJ, Ren LN, Zhang ZH, Gao Y, Li YZ, Shi JP, Qi GX. Prospective multi-center study of female patients with ST-elevation myocardial infarction in Liaoning province, China. *Chin Med J (Engl)*. 2012;125:1915–1919.
28. Shao XH, Yang YM, Zhu J, Liu LS; China CREATE Investigation Group. Comparison on therapeutic approach and short-term outcomes between male and female patients with ST-segment elevation myocardial infarction. *Zhonghua Xin Xue Guan Bing Za Zhi*. 2012;40:108–114.
29. Gao R, Patel A, Gao W, Hu D, Huang D, Kong L, Qi W, Wu Y, Yang Y, Harris P, Algert C, Groenestein P, Turnbull F; CPACS Investigators. Prospective observational study of acute coronary syndromes in China: practice patterns and outcomes. *Heart*. 2008;94:554–560.
30. Liu Q, Zhao D, Liu J, Wang W, Liu J; Bridging the Gap on Coronary Heart Disease Secondary Prevention in China. Current clinical practice patterns and outcome for acute coronary syndromes in China: results of BRIG project. *Zhonghua Xin Xue Guan Bing Za Zhi*. 2009;37:213–217.
31. Liu X, Chen YD, Lü SZ, Jin ZN, Liu H, Song XT; China GRACE Investigation Group. Analysis of the risk factors of patients with acute coronary syndrome sufferin hemorrhage during hospitalization. *Zhonghua Xin Xue Guan Bing Za Zhi*. 2012;40:902–907.
32. Zhao FH, Chen YD, Song XT, Pan WQ, Jin ZN, Yuan F, Li YB, Ren F, Lü SZ; Sino-GRACE investigators. Predictive factors of recurrent angina after acute coronary syndrome: the global registry acute coronary events from China (Sino-GRACE). *Chin Med J (Engl)*. 2008;121:12–16.
33. Cao Q, Shi L, Wang H, Dong K. Report from China: health insurance in China–evolution, current status, and challenges. *Int J Health Serv*. 2012;42:177–195.

Downloaded from http://circoutcomes.ahajournals.org/ at Yale University on November 25, 2013

34. Hu S, Tang S, Liu Y, Zhao Y, Escobar ML, de Ferranti D. Reform of how health care is paid for in China: challenges and opportunities. *Lancet*. 2008;372:1846–1853.
35. Chen Z. Launch of the health-care reform plan in China. *Lancet*. 2009;373:1322–1324.
36. Ministry of Health of the People's Republic of China. *China Public Health Statistical Yearbook 2011*. Beijing: Peking Union Medical College Publishing House; 2011.
37. Ministry of Health of the People's Republic of China. *The Performance Evaluation Standards for General Hospitals (Revised Version)*. 2009. http://www.moh.gov.cn/cmsresources/mohbgt/cmsrsdocument/doc6535.pdf. Accessed May 28, 2013.
38. Ministry of Health of the People's Republic of China. *China Public Health Statistical Yearbook 2003*. Beijing: Peking Union Medical College Publishing House; 2003.
39. Peterson ED, Roe MT, Rumsfeld JS, Shaw RE, Brindis RG, Fonarow GC, Cannon CP. A call to ACTION (acute coronary treatment and intervention outcomes network): a national effort to promote timely clinical feedback and support continuous quality improvement for acute myocardial infarction. *Circ Cardiovasc Qual Outcomes*. 2009;2:491–499.
40. Lichtman JH, Lorenze NP, D'Onofrio G, Spertus JA, Lindau ST, Morgan TM, Herrin J, Bueno H, Mattera JA, Ridker PM, Krumholz HM. Variation in recovery: role of gender on outcomes of young AMI patients (VIRGO) study design. *Circ Cardiovasc Qual Outcomes*. 2010;3:684–693.
41. American Hospital Association. *AHA Annual Survey Database Fiscal Year 2011*. http://www.ahadataviewer.com/book-cd-products/aha-survey/. Accessed May 27, 2013.
42. Vogel RA. HCFA's Cooperative Cardiovascular Project: a nationwide quality assessment of acute myocardial infarction. *Clin Cardiol*. 1994;17:354–356.
43. Ellerbeck EF, Jencks SF, Radford MJ, Kresowik TF, Craig AS, Gold JA, Krumholz HM, Vogel RA. Quality of care for Medicare patients with acute myocardial infarction: a four-state pilot study from the Cooperative Cardiovascular Project. *JAMA*. 1995;273:1509–1514.
44. Marciniak TA, Ellerbeck EF, Radford MJ, Kresowik TF, Gold JA, Krumholz HM, Kiefe CI, Allman RM, Vogel RA, Jencks SF. Improving the quality of care for Medicare patients with acute myocardial infarction: results from the Cooperative Cardiovascular Project. *JAMA*. 1998;279:1351–1357.
45. Chinese Center for Disease Control and Prevention. *Early diagnosis and treatment are low for patients with acute myocardial infarction in China*. http://www.chinacdc.cn/mtdx/mxfcrxjbxx/201210/t20121015_70567.htm. Accessed February 11, 2013.
46. Chen ZM, Pan HC, Chen YP, Peto R, Collins R, Jiang LX, Xie JX, Liu LS; COMMIT (Clopidogrel and Metoprolol in Myocardial Infarction Trial) collaborative group. Early intravenous then oral metoprolol in 45,852 patients with acute myocardial infarction: randomised placebo-controlled trial. *Lancet*. 2005;366:1622–1632.
47. Chen ZM, Jiang LX, Chen YP, Xie JX, Pan HC, Peto R, Collins R, Liu LS; COMMIT (ClOpidogrel and Metoprolol in Myocardial Infarction Trial) collaborative group. Addition of clopidogrel to aspirin in 45,852 patients with acute myocardial infarction: randomised placebo-controlled trial. *Lancet*. 2005;366:1607–1621.

Downloaded from http://circoutcomes.ahajournals.org/ at Yale University on November 25, 2013
