# Supplementary material for: Trends in Cardiac Biomarker Testing in China for Patients with Acute Myocardial Infarction, 2001 to 2011: China PEACE-Retrospective AMI Study
Source: PLoS One. 2015 Apr 20;10(4):e0122237. doi: 10.1371/journal.pone.0122237 (PMC4404305; doi:10.1371/journal.pone.0122237)
Supplement: S1 Protocol Suppl — (DOCX) [file pone.0122237.s005.docx]

**SUPPLEMENTAL MATERIAL**

The China PEACE (Patient-centered Evaluative Assessment of Cardiac Events) Retrospective

Study of Acute Myocardial Infarction: Study Design

The China PEACE Collaborative Group1, 2

1State Key Laboratory of Cardiovascular Disease, China Oxford Centre for International Health Research, Fuwai Hospital, National Center for Cardiovascular Diseases, Chinese Academy of Medical Sciences and Peking Union Medical College, Beijing, 100037, People's Republic of China; 2The Center for Outcomes Research and Evaluation, Yale-New Haven Hospital, New Haven, Connecticut, United States

**TABLE OF CONTENTS**

Pages 3-5: China PEACE-Retrospective AMI Study Sampling Design

Page 6: China PEACE-Retrospective AMI Study Absolute Precision Achieved for In-Hospital

Mortality in Rural and Urban Study Strata

Pages 7: China PEACE-Retrospective AMI Study Quality Assurance and Quality Control

Strategies in Medical Record Sampling

Pages 8-9: China PEACE-Retrospective AMI Study Quality Assurance and Quality Control

Strategies in Medical Record Abstraction

Pages 10-39: China PEACE-Retrospective AMI Study Case Report Form, Part 1

Pages 40-54: China PEACE-Retrospective AMI Study Case Report Form, Part 2

Pages 55-129: China PEACE-Retrospective AMI Study Data Dictionary

**CHINA PEACE-RETROSPECTIVE AMI STUDY SAMPLING DESIGN**

**Background**

We intended study hospitals to reflect diverse sites of care in China. As hospital volumes and clinical capacities differ between urban and rural areas as well among the 3 official economic- geographic regions of China, we separately identified hospitals in 5 strata: Eastern-rural,

Central-rural, Western-rural, Eastern-urban, and Central/Western-urban regions. We considered an area urban if it is part of a downtown or suburban area within a direct-controlled municipality (Beijing, Tianjin, Shanghai, Chongqing) or 1 of 283 prefectural-level cities. We considered surrounding county-level regions, including counties and county-level cities, to be rural. Within this framework, China is composed of 287 urban regions and 2010 rural regions. We considered Central and Western urban regions together given their similar per capita income and health services capacity as shown below:

**Population, Economy, and Hospitals in Different Geographic Strata of Mainland China**

**Eastern Central Western**

**Rural Setting**

**Population*** 256,899,053 205,567,264 222,491,738

**Income per capita (RMB)†** 9,256 6,351 5,604

**Level of central hospital**

**Tertiary (%)** 33 (5%) 12 (2%) 30 (3%) **Secondary (%)** 586 (92%) 462 (92%) 739 (85%) **Primary (%)** 20 (3%) 26 (5%) 102 (12%) **Total** 639 500 871

**Urban Setting**

**Population*** 336,364,491 150,467,917 144,803,916

**Income per capita (RMB)†** 21,547 15,539 15,523

**Median # of hospitals per urban area (IQR)‡**

**Tertiary** 3 (2-6) 2 (1-3) 2 (1-4)

**Secondary** 5 (3-8) 4 (3-6) 3 (2-6)

* Statistics in 2009 from the National Bureau of Statistics of China

(<http://www.stats.gov.cn/tjsj/ndsj/2010/indexch.htm)>

† Statistics in 2009 from the National Bureau of Statistics of China

[(h](http://www.stats.gov.cn/tjsj/ndsj/2010/indexch.htm))t[tp://www.stats.gov.cn/tjsj/ndsj/2010/indexch.htm)](http://www.stats.gov.cn/tjsj/ndsj/2010/indexch.htm))

‡ Median (interquartile range)

We identified cases for study inclusion using a stratified 2-stage cluster sampling design. In the

first stage, we identified hospitals using a simple random sampling procedure within each of the

5 study strata. In the 3 rural strata, the sampling framework consisted of the central hospital in each of the predefined rural regions (2010 central hospitals in 2010 rural regions). Within each rural region, the central hospital is the largest general hospital with the greatest clinical capacity for treating acute illness. In each of the 2 urban strata, the sampling framework consisted of the highest-level hospitals in each of the predefined urban regions (833 hospitals in 287 urban regions). Hospital level is officially defined by the Chinese government based on clinical

resource capacity. For example, secondary hospitals have at least 100 inpatient beds and the

capacity to provide acute medical care and preventive care services to populations of at least

100,000, while tertiary hospitals are large referral centers in provincial capitals and major cities. We excluded military hospitals, prison hospitals, specialized hospitals without a cardiovascular disease division, and traditional Chinese medicine hospitals. Since the majority of hospitals in China are publicly owned and administered, hospital closure is rare, and hospital number has remained stable over the past decade. We therefore decided to select representative hospitals from 2011 to reflect current practices and trace this cohort backward to 2006 and 2001 to describe temporal trends.

In the second stage, we drew cases based on the local hospital database for patients with acute myocardial infarction at each sampled hospital. We ordered each hospital’s list of eligible cases by date of admission and selected cases using systematic random sampling with equal probabilities. We selected a case at random, after which we selected every *kth* case based on

sample size requirements, where *k* is the sampling interval.

In each of the 5 study strata, we determined the sample size required to achieve a 2% precision for describing the primary outcome, in-hospital mortality, which we had estimated to be approximately 9% in urban hospitals and 7% in rural county-level hospitals.

The following *Equation 1* can be used to define the sample size required (*n*) for a given proportion of the primary outcome (*P*), desired precision (*d*), and specific choice of *α*.

*Equation 1:*

𝑛 =

��∝ ! ∙ 1 − 𝑃

��!

However, because random cases sampled within the same hospital are likely to be more similar to one another than to random cases from another hospital, the effective sample size is reduced. Consequently, a design effect adjustment should be introduced as follows:

*Equation 2:*

𝑛 =

��∝ ! ∙ 1 − 𝑃

��!

×  𝑑𝑒𝑓𝑓

Where the design effect (*deff*) is given by

*Equation 3:*

𝑑𝑒𝑓�� = 1 +  ��  (��′ − 1)

where 𝛿 is the intraclass correlation for the statistic in question and ��′ is the average number of sampled cases within each hospital. ��′ is also known as the cluster size.

With this framework in mind, to achieve a precision of 2% with an *α* of 0.05 in each of the 3 rural strata, assuming an intraclass correlation of 0.02 and design effect of 1.8, we would need to sample 1150 medical records among hospitals with an average cluster size of 40. Analogously, to achieve a precision of 2% with an *α* of 0.05 in each of the 2 urban strata, assuming an intraclass correlation of 0.02 and design effect of 2.2, we would need to sample 1750 medical records among hospitals with an average cluster size of 60. These cluster sizes in rural and urban settings appeared reasonable based upon our previous survey of treatment for acute coronary syndromes at more than 1000 hospitals in 2010, which demonstrated that the median volume of hospitalization for acute myocardial infarction was approximately 180 cases per year in urban hospitals and 95 cases per year in rural county-level hospitals. Assuming an

anticipated participation rate of 85% among selected hospitals, we approached 35 hospitals for participation in each stratum for a total of 175 hospitals (70 urban and 105 rural). We doubled cluster sizes for 2011 to improve precision in the description of hospital-level treatment patterns and outcomes.

Consequently, the total expected sample volume with the above assumptions was

approximately 6,950 cases in 2001, 6,950 cases in 2006, and 13,900 cases in 2011.

**CHINA PEACE-RETROSPECTIVE AMI STUDY ABSOLUTE PRECISION ACHIEVED FOR IN- HOSPITAL MORTALITY IN RURAL AND URBAN STUDY STRATA**

**Study Stratum Sample**

**Size**

**Achieved**

**Actual Data***

**Average Cluster Size**

***deff* Absolute Precision for Rural**

**Strata**

**(7% Mortality)**

**Absolute Precision for Urban Strata**

**(9% Mortality)**

| Western-rural in 2001 | 160 | 5 | 1.09 | 3.9% | --- |
| --- | --- | --- | --- | --- | --- |
| Western-rural in 2006 | 357 | 12 | 1.22 | 2.9% | --- |
| Western-rural in 2011 | 765 | 26 | 1.49 | 2.2% | --- |
| Central-rural in 2001 | 284 | 9 | 1.17 | 3.3% | --- |
| Central-rural in 2006 | 554 | 18 | 1.35 | 2.5% | --- |
| Central-rural in 2011 | 1,359 | 45 | 1.89 | 1.9% | --- |
| Eastern-rural in 2001 | 559 | 19 | 1.35 | 2.4% | --- |
| Eastern-rural in 2006 | 1,082 | 36 | 1.70 | 2.0% | --- |
| Eastern-rural in 2011 | 1,991 | 66 | 2.31 | 1.7% | --- |
| Central/Western-urban in 2001 | 482 | 16 | 1.30 | --- | 2.9% |
| Central/Western-urban in 2006 | 1,230 | 41 | 1.80 | --- | 2.1% |
| Central/Western-urban in 2011 | 2,569 | 86 | 2.69 | --- | 1.8% |
| Eastern-urban in 2001 | 1,313 | 44 | 1.86 | --- | 2.1% |
| Eastern-urban in 2006 | 1,976 | 66 | 2.30 | --- | 1.9% |
| Eastern-urban in 2011 | 3,950 | 132 | 3.61 | --- | 1.7% |
| **Anticipated Data†** | | | | | |
| Rural in 2001 or 2006 | 1,150 | 38 | 1.75 | 2.0% | --- |
| Urban in 2001 or 2006 | 1,750 | 58 | 2.15 | --- | 1.9% |
| Rural in 2011 | 2,300 | 77 | 2.51 | 1.6% | --- |
| Urban in 2011 | 3,500 | 117 | 3.31 | --- | 1.7% |

* Actual data reflects the absolute precision achieved in describing in-hospital mortality in each rural and urban stratum during 2001, 2006, and 2011 based upon the actual sample size, actual average cluster size, and associated design effect.

† Anticipated data reflects the anticipated absolute precision that would be achieved in describing in-hospital mortality in each rural and urban stratum during 2001, 2006, and 2011 based upon the anticipated sample size, anticipated average cluster size, and associated design effect.

**CHINA PEACE-RETROSPECTIVE AMI STUDY QUALITY ASSURANCE AND QUALITY CONTROL STRATEGIES IN MEDICAL RECORD SAMPLING**

As the China PEACE Retrospective Study of Acute Myocardial Infarction was designed to study a nationally representative hospital cohort, we selected hospitals based on random sampling rather than previous collaboration or longstanding experience with retrospective data collection. As we anticipated that many hospitals would have little previous experience with clinical research, we provided participating sites with substantial support to ensure adherence with multiple quality control strategies for identifying the universe of hospitalizations for acute myocardial infarction and the subset sampled for the China PEACE-Retrospective AMI Study.

For all participating hospitals, we first held a local investigator meeting to provide in-depth information on study design and operating procedures. We trained sites on how to identify their universe of hospitalizations for acute myocardial infarction. We specified 3 separate approaches. Our first preference was that sites query their electronic database of hospitalizations for patients discharged with a principal discharge diagnosis of acute myocardial infarction using ICD-9 codes 410 or ICD-10 codes I21. If such a database was not available, we required that site coordinators manually search the written hospitalization log in the hospital archiving office for hospitalizations for acute myocardial infarction. Site coordinators reviewed the original medical record in cases where the diagnosis of acute myocardial infarction was uncertain. Finally, as the least preferred option, we asked that study coordinators manually search the written log of hospitalizations of each hospital ward for cases for acute myocardial infarction. Site coordinators reviewed the original medical records in cases of uncertain diagnosis.

To verify compliance with the search strategy, research staff from the study coordinating center visited 46 study sites to repeat the search process and confirm that the list of hospitalizations with acute myocardial infarction was complete. These 46 sites provided approximately 60% of all hospitalizations for acute myocardial infarction from which we sampled cases for the PEACE Retrospective AMI Study.

After we drew case samples at each hospital using systematic random sampling and assigned each record a unique study ID, we required local investigators to gather the original record, assign it its study ID, scan it, and transmit the scanned copy to the coordinating center. Upon receipt of the scanned record, coordinating center staff verified the accuracy of the study ID and ensured that the record itself was complete and legible. To facilitate this process, the coordinating center provided each study site with a high-speed scanner. In addition,

coordinating center staff provided on-site assistance for almost 50 study sites that provided approximately 50% of sampled cases.

To ensure transparency in all sampling performed by the NCCD, we have recorded all sampling procedures including the contents of the sampling framework database that contains all eligible cases for sampling in their predefined sequence and the seeds used in random number generation.

**CHINA PEACE-RETROSPECTIVE AMI STUDY QUALITY ASSURANCE AND QUALITY CONTROL STRATEGIES IN MEDICAL RECORD ABSTRACTION**

**Background**

Medical record abstraction can be guided by the types of data being abstracted. We have defined as *hard data elements* those elements that can be abstracted directly from the chart without use of professional judgment. Examples include the date of admission, patient sex, patient age, serum creatinine on hospital day 1, etc. Based on the results of a pilot study of 500 medical records, we required that a data element be abstracted with greater than 98% accuracy to be considered *hard*. The quality of abstraction of hard data elements depends primarily on

the conscientiousness of abstractors as well as the legibility of the records themselves.

In contrast, *soft data elements* are those that require more advanced medical knowledge for abstraction. Examples of soft data elements include the presence of comorbidities, evidence of pulmonary edema on hospital presentation, development of post-procedural complications such as bleeding or arrhythmia, and so on.

Within the Chinese medical record, hard data elements are found predominantly in the medical record face sheet and section for laboratory testing results. Soft elements are found throughout all other sections of the medical record including the admission record, discharge record, physician orders, diagnostic reports, etc.

**Training and Qualification of Abstractors**

China PEACE has made use of abstractors with and without formal medical training. Abstractors without formal medical training were hired from companies with experience in medical data entry and/or medical record abstraction. Abstractors with medical training included both undergraduate and post-graduate students from medical school.

Each abstractor was given a set of training materials about medical record abstraction, including *CHINA PEACE: A Brief Introduction*, *China PEACE: Operation Manual of Medical Record Abstraction*, and 10 standard training medical records.

Each abstractor also underwent the following training courses: (1) Introduction to the China PEACE protocol; (2) Coronary heart disease and its subtypes; (3) Component parts of the inpatient medical record and their contents; (4) China PEACE Retrospective Study of Acute Myocardial Infarction data dictionary; (5) Frequently asked questions in medical record abstraction; (6) Quality assurance and quality control measures in China PEACE; and (7) Intensive guidance in abstracting 10 standard training medical records followed by group discussion and retraining as needed.

Once training was completed, each abstractor reviewed 10 standard training medical records. Supervisors were responsible for evaluating the integrity of abstraction. For qualification, accuracy of abstraction had to be greater than 98 percent, including for soft data elements. Failure to meet this standard resulted in additional training as required.

**Quality Control in Medical Record Abstraction**

We assigned the Front Page and Laboratory Results sections of the medical records to qualified non-medical abstractors, as these sections contain almost exclusively hard data elements. We performed a second abstraction of all records abstracted by non-medical abstractors to ensure accuracy.

Abstractors with formal medical training abstracted all sections of the medical chart containing soft data elements. These include the admission note, daily progress notes, procedure notes, diagnostic testing reports, medication administration record, physician orders, nursing notes, and discharge summary. We randomly audited approximately 5% of the abstracted records. If the records were not abstracted with 98% accuracy, all medical records in the audited batch were considered unqualified and were re-reviewed by a different abstractor. Discrepancies in abstraction were resolved by review of the original medical record.

To minimize abstraction errors, abstractors started by abstracting only printed medical records. After gaining experience, these individuals were allowed to begin abstracting hand-written records. In addition, a physician was always present in the room with abstractors or was available online to answer questions and address areas of concern as they arise. Common problems have led to updates and further customization of the data dictionary and web-based data management program built for the China PEACE-Retrospective AMI Study into which data were directly entered. This computer program has been customized to expedite the

identification of medications that may have more than one trade name. Furthermore, medical records belonging to the same hospital and year were assigned to a broad group of reviewers to avoid potential residual disparities in quality among different abstractors

**Data Management and Cleaning**

Ongoing data cleaning is performed in a systematic manner. Data is regularly queried for invalid and illogical values as well as for duplicate record entry. Invalid values are identified as outliers in continuous data distributions. Duplicate records are identified by the presence of similar study identification numbers, hospital identification numbers, medical record identification numbers, and the date of discharge. Once a potential error is found, the issue is resolved after tracing and reviewing the relevant records.

Frequent sources of error result in additional intensive training for abstractors and supervisors, if necessary. The study data dictionary is updated, as needed.

**CHINA PEACE-RETROSPECTIVE AMI STUDY CASE REPORT FORM, PART 1**

**Contents**

Face Sheet .............................................................................................................. 111

Discharge Records .................................................................................................... 11

Admission Records & History of Diseases ................................................................ 17

Personal History ........................................................................................................ 21

Physical Examination............................................................................................... 211

Auxiliary Examination ................................................................................................ 22

Daily Records ............................................................................................................ 26

In-Hospital Device Placement.................................................................................... 29

Coronary Angiography ............................................................................................. 300

PCI & PTCA Reports ............................................................................................... 311

Nursing Records ...................................................................................................... 333

CABG Report ........................................................................................................... 333

Imaging Examination ............................................................................................... 334

In-Hospital ECG ......................................................................................................... 35

Long-term Physician Orders ...................................................................................... 36

Short-term Physician Orders ..................................................................................... 37

Discharge Medications .............................................................................................. 38

Temperature Report ................................................................................................... 39

**Note:** The case report form parallels common sections of the Chinese medical record. In certain instances, questions within the case report form have been repeated for different sections of the medical record to maximize the sensitivity of abstraction.

**Part 1**

**1 Study ID _____**【 **Free Text (F)**】

**Face Sheet**

**2 Medical Record Number _____**【 **F**】

**3 Hospital Number _____**【 **F**】

**4 Gender**【 **Single Choice (SC)**】

--Male

--Female

--Unrecorded

**5.1 Admission Date _____**【 **Select Date from Calendar (C)**】

**5.2 Admission Time _____**【 **F**】

**Discharge Records**

**6 In-Hospital Death**【 **SC**】

--Yes

--No

**7 In-Hospital Death Date _____**【 **C**】

**8 Admission Diagnosis (Related to Coronary Heart Disease)**

【 **Multiple Choices Permitted (MC)**】

--Coronary Heart Disease

--Acute Coronary Syndrome

--Acute Extensive Anterior Myocardial Infarction

--Acute Myocardial Infarction

--Acute Anterior Myocardial Infarction

--Acute Septal Myocardial Infarction

--Acute Inferior Myocardial Infarction

--Acute Lateral Myocardial Infarction

--Acute Posterior Myocardial Infarction

--Acute Right Ventricular Myocardial Infarction

--Acute Non ST-Elevation Myocardial Infarction

--Acute ST-Elevation Myocardial Infarction

--Subendocardial Myocardial Infarction

--Acute Myocardial Infarction Suspected

--Previous Q-wave Myocardial Infarction

--Unstable Angina Pectoris

--Stable Angina Pectoris

--Prinzmetal's Angina

--Angina (Unrecorded Subtype)

--None of the Above Is Recorded

**9 Admission Diagnosis (Unrelated to Coronary Heart Disease)**【 **MC**】

--Cardiac Arrest

--Cardiogenic Shock

--Heart Failure

--Ventricular Fibrillation/Ventricular Tachycardia

--Hypertension

--Dyslipidemia

--Diabetes Mellitus

--Diabetic Nephropathy

--Hemorrhagic Stroke (Cerebral Hemorrhage/Subarachnoid Hemorrhage)

--Ischemic Stroke (Cerebral Infarction/Cerebral Embolism/Cerebral Thrombosis)

--Stroke (Unspecified)

--Pneumonia

--COPD Exacerbation

--Gastrointestinal Bleeding

--Hepatitis

--Cirrhosis

--Chronic Renal Failure

--Acute Renal Failure

--Dialysis (Hemodialysis/Peritoneal Dialysis)

--Trauma

--None of the Above Is Recorded

**10 Summary of In-Hospital Events**

【 **Every Item Has Three Options—Y/N/Unrecorded**】

--Repeat/Recurrent Angina

--Repeat/Recurrent Myocardial Infarction

--Cardiac Rupture

--Papillary Muscle Rupture

--Ventricular Septal Perforation

--Cardiac Tamponade

--Cardiogenic Shock

--Cardiac Arrest

--Atrial Fibrillation or Flutter

--Ventricular Tachycardia/Ventricular Fibrillation

--Heart Failure (New Onset)

--Gastrointestinal Bleeding

--Genitourinary Bleeding

--Intracranial/Subdural Bleeding

--Retroperitoneal Bleeding

--Access Site Bleeding (Including Hematoma at Access Site)

--Pericardial Bleeding

--Bleeding (Unspecified)

--Hemorrhagic Shock

--Venous Thromboembolism

--Pulmonary Embolism

--Deep Vein Thrombosis

--Peripheral Embolization

--Access Site Arteriovenous Fistula

--Ischemic Stroke (Cerebral Infarction/Thrombosis/Cerebral Embolism)

--Hemorrhagic Stroke (Cerebral Hemorrhage/Subarachnoid Hemorrhage)

--Stroke (Unspecified)

--Acute Renal Failure

--Dialysis (Hemodialysis/Peritoneal Dialysis)

--Infection (Nosocomial Infection)

--Contrast Reaction

--Septicemia

**11 In-Hospital Device**【 **Every Item Has Three Options—Y/N/Unrecorded**】

--In-Hospital Implantation of Intra-Aortic Balloon Pump (IABP)

--In-hospital Implantation of a Permanent Pacemaker

--In-hospital Implantation of Implantable Cardioverter Defibrillator (ICD)

**12 Did The Patient Refuse the Following Treatment?**

【 **Every Item Has Three Options—Y/N/Unrecorded**】

--Refused Percutaneous Coronary Intervention (PCI)

--Refused Fibrinolysis

--Refused CABG

**13 Documented Reasons for Non-prescription of the Following Agents in the First 24**

**Hours**【 **MC**】

--Aspirin

--Beta-blocker

--Fibrinolysis

--None of Above Is Recorded

**14 If Applicable, Specify the Documented Reason for Non-prescription of Aspirin in the**

**First 24 Hours _____**【 **F**】

**15 If Applicable, Specify the Documented Reason for Non-prescription of Beta-blocker**

**in the First 24 Hours _____**【 **F**】

**16 If Applicable, Specify the Documented Reason for Non-prescription of Fibrinolysis**

**in the First 24 Hours _____**【 **F**】

**17 Documented Reasons for Non-prescription of the Following Agents at Discharge**

【 **MC**】

--Aspirin

--Angiotensin Converting Enzyme Inhibitors (ACE Inhibitor)

--Angiotensin Receptor Blocker (ARB)

--Beta-blocker

--Statin

--None of Above Is Recorded

**18 If Applicable, Specify the Documented Reason for Non-prescription of Aspirin at**

**Discharge _____**【 **F**】

**19 If Applicable, Specify the Documented Reason for Non-prescription of ACE Inhibitor**

**at Discharge_____**【 **F**】

**20 If Applicable, Specify the Documented Reason for Non-prescription of ARB at**

**Discharge _____**【 **F**】

**21 If Applicable, Specify the Documented Reason for Non-prescription of Beta-blocker**

**at Discharge _____**【 **F**】

**22 If Applicable, Specify the Documented Reason for Non-prescription of Statin at**

**Discharge _____**【 **F**】

**23 Discharge Status**【 **SC**】

--Patient or Relatives Demand Transfer to another Hospital

--Physician Suggests Transfer to another Hospital

--Patient Left against Medical Advice

--Other

--None of the Above is Recorded

**24 Discharge Diagnosis (Related to Coronary Heart Disease)**【 **MC**】

--Coronary Heart Disease

--Acute Coronary Syndrome

--Acute Extensive Anterior Myocardial Infarction

--Acute Anterior Myocardial Infarction

--Acute Septal Myocardial Infarction

--Acute Inferior Myocardial Infarction

--Acute Lateral Myocardial Infarction

--Acute Posterior Myocardial Infarction

--Acute Right Ventricular Myocardial Infarction

--Acute Non ST-Elevation Myocardial Infarction

--Acute ST-Elevation Myocardial Infarction

--Subendocardial Myocardial Infarction

--Acute Myocardial Infarction

--Acute Myocardial Infarction Suspected

--Previous Q-Wave Myocardial Infarction

--Unstable Ang**i**na Pectoris

--Stable Angina Pectoris

--Prinzmetal's Angina

--Angina (Unrecorded Subtype)

--Repeated/Recurrent Unstable Angina

--Repeated/Recurrent Myocardial Infarction

--None of the Above Is Recorded

**25 Discharge Diagnosis**【 **MC**】

--Cardiac Rupture

--Papillary Muscle Rupture

--Ventricular Septal Perforation

--Cardiac Tamponade

--Cardiogenic Shock

--Cardiac Arrest

--Atrial Fibrillation or Flutter

--Ventricular Tachycardia/Ventricular Fibrillation

--Heart Failure (New Onset)

--Gastrointestinal Bleeding

--Genitourinary Bleeding

--Intracranial/Subdural Bleeding

--Retroperitoneal Bleeding

--Access Site Bleeding (Including Hematoma at Access Site)

--Pericardial Bleeding

--Bleeding (Unspecified)

--Hemorrhagic Shock

--Venous Thromboembolism

--Pulmonary Embolism

--Deep Vein Thrombosis

--Peripheral Embolization

--Access Site Arteriovenous Fistula

--Ischemic Stroke (Cerebral Infarction/Thrombosis/Cerebral Embolism)

--Hemorrhagic Stroke (Cerebral Hemorrhage/Subarachnoid Hemorrhage)

--Stroke (Unspecified)

--Pneumonia

--COPD Exacerbation

--Acute Renal Failure

--Chronic Renal Failure

--Dialysis (Hemodialysis/Peritoneal Dialysis)

--Infection (Nosocomial Infection)

--Septicemia

--Contrast Reaction

--Dyslipidemia

--Hypertension

--Diabetes Mellitus

--Diabetic Nephropathy

--Trauma

--Hepatitis

--Cirrhosis

--Anemia

--None of Above Is Recorded

**26 Discharge Suggestions**

【 **Every Item Has Three Options—Y/N/Unrecorded**】

--Regular Blood Lipid Assessment

--Dietary Improvement

--Weight Reduction

--Smoking Cession

--Regular Exercise

--PCI

--CABG

--None of Above Is Recorded

**27 Medications at Discharge _____**【 **Drug Database(D)**】

**28 Administration**【**SC**】

--P.O. /Undefined

--I.V. drip/ I.V. gtt (intravenously guttae)

--I.V.

--I.H. (Hypodermic Injection)

--I.D. (Intradermal Injection)

--Others

**Dose _____**【 **F**】

**Unit** 【 **SC**】

--g

--mg

--ml

--U

--Piece/ #

--mg/kg

--mg/h

--mg/min

--mg/kg·h

--ml/kg

--ml/h

--ml/min

--ml/kg·min

--U/kg

--U/min

--U/h

--U/kg·h

--ug

--ug/kg

--ug/min

--ug/kg·min

--MU

--BU

--%

--Other

--Unrecorded

**Frequency**【 **SC**】

--Qd /QN

--Bid/q12h

--Tid/q8h

--Q6h

--As Needed

--Other

--Unrecorded

【**29-56**】 **Repeat Questions No.27 to 28 for the Other 14 Available Agents**

**Admission Records & History of Diseases**

**57.1 Ischemic Symptoms Onset Date _____ Days Ago**【 **F**】

**57.2 Ischemic Symptoms Onset Time _____ Hours Ago**【 **F**】

**58.1 Ischemic Symptoms Onset Date _____**【 **C**】

**58.2 Ischemic Symptoms Onset Time _____**【 **F**】

**59 Was the time of Ischemic Symptom Onset Estimated?**【 **SC**】

--Yes

--No

**60 Did the Patient Have Chest Discomfort (Chest Pain/Chest Discomfort/Chest**

**Pressure/Other Symptoms in Chest)?**【 **SC**】

--Yes

--No

--Unrecorded

**61 Did the Chest Discomfort Last 10 or More Minutes?**【 **SC**】

--Yes

--No

--Unrecorded

**62 Did the patient Have Other Ischemic Symptoms (Shortness of Breath/Pain at**

**Non-chest Sites/Nausea/Vomit/Fatigue)?**【 **SC**】

--Yes

--No

--Unrecorded

**63 Did Other Ischemic Symptoms Last 10 or More Minutes?**【 **SC**】

--Yes

--No

--Unrecorded

**64 Means of Transport to the First Facility**【 **SC**】

--Ambulance

--Self/Family

--Other

--Unrecorded

**65.1 Patient was Taken by Ambulance to the Hospital _____ Days Ago**【 **F**】

**65.2 Patient was Taken by Ambulance to the Hospital at the Following Time _____**

**Hours Ago**【 **F**】

**66.1 Date When Patient was Taken by Ambulance to the Hospital _____**【 **C**】

**66.2 Time When Patient was Taken by Ambulance to the Hospital _____**【 **F**】

**67 Was the Time at Which the Patient was Taken by Ambulance to the Hospital**

**Estimated?**【 **SC**】

--Yes

--No

**68 Was Medical Assistance Received in an Outside Facility Prior to This**

**Hospitalization?**【 **SC**】

--Yes

--No

--Unrecorded

**69.1 Arrival at Outside Facility Date, _____Days Ago**【 **F**】

**69.2 Arrival at Outside Facility Time, _____ Hours Ago**【 **F**】

**70.1 Date of Arrival at Outside Facility _____**【 **C**】

**70.2 Time of Arrival at Outside Facility _____**【 **F**】

**71 Was the Time at Which Patient Arrived at the Outside Facility Estimated?**【 **SC**】

--Yes

--No

**72.1 Transfer from Outside Facility Date, _____ Days Ago**【 **F**】

**72.2 Transfer from Outside Facility Time, _____ Hours Ago**【 **F**】

**73.1 Transfer from Outside Facility Date _____**【 **C**】

**73.2 Transfer from Outside Facility Time _____**【 **F**】

**74 Was the Time of Transfer from the Outside Facility Estimated?**【 **SC**】

--Yes

--No

**75 Name of the Transferring Facility _____**【 **F**】

**76 Means of Transfer**【 **SC**】

--Ambulance

--Air

--Other

--Unrecorded

**77 Treatment Prior to Arrival**【 **Every Item Has Three Options—Y/N/Unrecorded**】

--Aspirin

--Clopidogrel

--Ticlopidine

--Antiplatelet Therapy

--Fibrinolysis (If Yes, Specify: SK, UK, Rt-PA, Other)

--Glucose Insulin Potassium (GIK) Solution

--External Defibrillation

--CPR/Chest Compression

--Temporary Cardiac Pacing

**78 Time of Arrival at Your Hospital _____**【 **F**】

**79 Was Time of Arrival at Your Hospital Estimated?**【 **SC**】

--Yes

--No

--Unrecorded

**80 Treatment in Emergency Room Prior to Admission**

【 **Every Item Has Three Options—Y/N/Unrecorded**】

--Aspirin

--Clopidogrel

--Ticlopidine

--Antiplatelet Therapy

--Fibrinolysis (If Yes, Specify: SK, UK, Rt-PA, Other)

--Glucose Insulin Potassium (GIK) Solution

--External Defibrillation

--CPR/Chest Compression

--Temporary Cardiac Pacing

**81.1 Cardiogenic Shock at Presentation**【 **SC**】

(Sustained (>30 minutes) Episode of Systolic Blood Pressure <90 mm Hg/Cardiac Index

<2.2L/min/m2/Requirement for Parenteral Inotropic or Vasopressor Agents/Mechanical

Support [e.g. IABP/Extracorporeal Circulation/Ventricular Assist Devices])

--Yes

--No

--Unrecorded

**81.2 Gastrointestinal Bleeding at Presentation** (Hematemesis/Hematochezia/Melena)

【 **SC**】

--Yes

--No

--Unrecorded

**82 Past Medical History (Related to Heart Diseases)**

【 **Every Item Has Three Options—Y/N/Unrecorded**】

--Coronary Heart Disease

--Angina Pectoris

--Myocardial Infarction

--Fibrinolysis

--PCI

--CABG

--Ventricular Tachycardia/Ventricular Fibrillation

--Atrial Fibrillation or Flutter

--Permanent Pacemaker

--AICD

--Heart Failure

**83.1 Specify the First Home Medication _____**【 **F**】

**83.2 Specify the Second Home Medication _____**【 **F**】

**83.3 Specify the Third Home Medication _____**【 **F**】

**83.4 Specify the Fourth Home Medication _____**【 **F**】

**83.5 Specify the Fifth Home Medication _____**【 **F**】

**84 Symptoms of Heart Failure**【 **Every Item Has Three Options—Y/N/Unrecorded**】

--Recurrent Dyspnea While Supine

--Worsening Dyspnea on Light Exertion

**85 Past Medical History (Not Directly Related to Heart Disease)**

【 **Every Item Has Three Options—Y/N/Unrecorded**】

--Hypertension

--Diabetes Mellitus

--Dyslipidemia

--Peripheral Vascular Disease (Intermittent Claudication/Lower Limb Arterial Embolism)

--Venous Thromboembolism

--Pulmonary Embolism

--Deep Vein Thrombosis

--Ischemic Stroke (Cerebral Embolism/Cerebral Infarction/Cerebral Thrombosis)

--Hemorrhagic Stroke (Cerebral Hemorrhage/Subarachnoid Hemorrhage)

--Stroke (Unspecified)

--Carotid Artery Surgery/Intervention

--Asthma

--Chronic Lung Disease

(COPD/Chronic Bronchitis/Emphysema/Asbestosis/Mesothelioma/Black

Lung Disease/Pneumoconiosis/Radiation Induced Pneumonitis/Radiation Fibrosis)

--Liver Disease History

--Major Surgery within the Past 4 Weeks

--Cancer Other Than Skin Cancer

--Chronic Renal Failure

--Currently On Dialysis

**86 Treatment for Cancer within the Past 6 Months**【 **SC**】

--Yes

--No

--Unrecorded

**87 Liver Disease**【 **MC**】

--History of Hepatitis B Infection

--History of Hepatitis C Infection

--Cirrhosis

--Unrecorded

**88 Drug Allergy _____**【 **F**】

**Personal History**

**89 Smoking History**【 **SC**】

--Never

--Past

--Current

--Unrecorded

**90 If Current, Duration _____ Years _____ Months**【 **F**】

**91 If Past, Duration _____ Years _____Months**【 **F**】

**92 Smoking Frequency _____Cigarettes/Day**【 **F**】

**93 Menopausal Status**【 **SC**】

--Yes

--No

--Unrecorded

**94 Pregnancy Status**【 **SC**】

--Yes

--No

**95 Postpartum (within 6 Weeks after Giving Birth) Status**【 **SC**】

--Yes

--No

**Physical Examination**

**96 Vital Signs**【 **F**】

--Temperature at Presentation to This Facility_____ Celsius

--Heart Rate at Presentation to This Facility ____ bpm

--Respiratory Rate at Presentation to This Facility _____bpm

--Systolic Blood Pressure at Presentation to This Facility _____mmHg

--Diastolic Blood Pressure at Presentation to This Facility _____mmHg

**97 Signs of Heart Failure**

【 **Every Item Has Three Options—Y/N/Unrecorded**】

--Jugular Venous Distension

--Rales

--Pulmonary Edema

--Fluid Retention (Lower Extremity Edema)

**Auxiliary Examination**

**98.1 Date of the First ECG at Hospital _____**【 **C**】 **(“20140101” Means No ECG )**

**98.2 Time of the First ECG at Hospital _____**【 **F**】

**99 Original Interpretation of ECG**【 **MC**】

--Acute MI

--Complete LBBB (Unspecified)

--ST-Elevation

--ST-Depression

--Previous MI (Q Wave)

--Ventricular Fibrillation

--Ventricular Tachycardia

--Atrial Fibrillation

--2nd Degree Atrioventricular Block Type 1

--2nd Degree Atrioventricular Block Type 2

--3nd Degree Atrioventricular Block

**100.1 Date of the Second ECG at Hospital____**【 **C**】 **(“20140101” Means No ECG )**

**100.2 Time of the Second ECG at Hospital _____**【 **F**】

**101 Original Interpretation of ECG**【 **MC**】

--Acute MI

--Complete LBBB (Unspecified)

--ST-Elevation

--ST-Depression

--Previous MI (Q Wave)

--Ventricular Fibrillation

--Ventricular Tachycardia

--Atrial Fibrillation

--2nd Degree Atrioventricular Block Type 1

--2nd Degree Atrioventricular Block Type 2

--3nd Degree Atrioventricular Block

**102.1 Date of the Third ECG at Hospital____**【 **C**】 **(“20140101” Means No ECG )**

**102.2 Time of the Third ECG at Hospital _____**【 **F**】

**103 Original Interpretation of ECG**【 **MC**】

--Acute MI

--Complete LBBB (Unspecified)

--ST-Elevation

--ST-Depression

--Previous MI (Q Wave)

--Ventricular Fibrillation

--Ventricular Tachycardia

--Atrial Fibrillation

--2nd Degree Atrioventricular Block Type 1

--2nd Degree Atrioventricular Block Type 2

--3nd Degree Atrioventricular Block

**104.1 Date of the Fourth ECG at Hospital ____**【 **C**】 **(“20140101” Means No ECG )**

**104.2 Time of the Fourth ECG at Hospital _____**【 **F**】

**105 Original Interpretation of ECG**【 **MC**】

--Acute MI

--Complete LBBB (Unspecified)

--ST-Elevation

--ST-Depression

--Previous MI (Q Wave)

--Ventricular Fibrillation

--Ventricular Tachycardia

--Atrial Fibrillation

--2nd Degree Atrioventricular Block Type 1

--2nd Degree Atrioventricular Block Type 2

--3nd Degree Atrioventricular Block

**106.1 Date of the Fifth ECG at Hospital____**【 **C**】 **(“20140101” Means No ECG )**

**106.2 Time of the Fifth ECG at Hospital _____**【 **F**】

**107 Original Interpretation of ECG**【 **MC**】

--Acute MI

--Complete LBBB (Unspecified)

--ST-Elevation

--ST-Depression

--Previous MI (Q Wave)

--Ventricular Fibrillation

--Ventricular Tachycardia

--Atrial Fibrillation

--2nd Degree Atrioventricular Block Type 1

--2nd Degree Atrioventricular Block Type 2

--3rd Degree Atrioventricular Block

**108 Was Chest X-ray or Other Lung Imaging (Chest CT/MRI) Performed?**【 **SC**】

--Yes

--No

**109 Chest X-ray/Other Lung Examination (Chest CT/MRI) Date _____**【 **C**】

**110 Chest X-ray/Other Lung Examination (Chest CT/MRI) Results**

【 **Every Item Has Two Options—Y/N**】

--Pulmonary Edema

--Pulmonary Demonstrable Infiltrate/Pneumonia

**111 Was CK Tested at the Prior Hospital or in the Emergency Room of Your Hospital?**

--Yes

--No

**112.1 CK Value _____**【 **F**】

**112.2 Unit of CK _____**【 **SC**】

--IU/L

--Ng/ml

--Mg/ml

--%

--Mg/IU

--Ml/IU

**112.3 CK Date _____**【 **C**】

**112.4 CK Time _____**【 **F**】

**113 Was CK-MB Tested at the Prior Hospital or in the Emergency Room of Your**

**Hospital?**

--Yes

--No

**114.1 CK-MB Value _____**【 **F**】

**114.2 Unit of CK-MB _____**【 **SC**】

--IU/L

--Ng/ml

--Mg/ml

--%

--Mg/IU

--Ml/IU

**114.3 CK-MB Date_____**【 **C**】

**114.4 CK-MB Time_____**【 **F**】

**115 Was Troponin Tested at the Prior Hospital or in the Emergency Room of Your**

**Hospital?**【 **MC**】

--Troponin I

--Troponin T

--Troponin (Unspecified)

--None

**116 Value for Troponin**

--Positive

--Negative

--Numerical Value

**117.1 Troponin I Value _____**【 **F**】

**117.2 Unit of Troponin I _____**【 **SC**】

--Ng/mL

--Other

**118.1 Troponin T Value _____**【 **F**】

**118.2 Unit of Troponin T _____**【 **SC**】

--Ng/mL

--Other

**119.1 Troponin (Unspecified) Value _____**【 **F**】

**119.2 Unit of Troponin (Unspecified) _____**【 **SC**】

--Ng/mL

--Other

**120.1 Troponin Test Date_____**【 **C**】

**120.2 Troponin Test Time _____**【 **F**】

**121 Lab Tests Performed at the Prior Hospital or in the Emergency Room**

**of Your Hospital?**

【 **Every Item Has Two Options--Y or N**】

-- Routine Blood Test

-- Blood Chemistry Test

-- Coagulation Test

**122.1 White Blood Cell (WBC) Value _____ X 109**【 **F**】

**122.2 Neutrophil Ratio _____ %**【 **F**】

**122.3 Hemoglobin Value _____ g/L**【 **F**】

**122.4 Platelet Count Value _____ x109**【 **F**】

**122.5 Routine Blood Test Date _____**【 **C**】

**123.1 ALT Value _____U/L**【 **F**】

**123.2 AST Value _____U/L**【 **F**】

**123.3 Glucose Value _____ mmol/L**【 **F**】

**123.4 Total Bilirubin Value _____umol/L**【 **F**】

**123.5 Creatinine Value _____umol/L**【 **F**】

**123.6 Blood Chemistry Test Date _____**【 **C**】

**124.1 PT Value _____s**【 **F**】

**124.2 APTT Value _____s**【 **F**】

**124.3 INR Value _____**【 **F**】

**124.4 Coagulation Test Date_____**【 **C**】

**125 Admission Diagnosis (Related to Coronary Heart Disease)**【 **MC**】 **Same As**

**No.9**

**126 Admission Diagnosis (Unrelated to Coronary Heart Disease)**【 **MC**】 **Same As**

**No.10**

**127 Killip Classification**【 **SC**】

--I

--II

--III

--IV

--Unrecorded

**128 NYHA Classification**【 **SC**】

--I

--II

--III

--IV

--Unrecorded

**Daily Records**

**129 Are Daily Records Included with this Medical Record?**【 **SC**】

--Yes

--No

**130 In-Hospital Events**

【 **Every Item Has Three Options—Y/N/Unrecorded**】

--Died

--Repeat/Recurrent Unstable Angina

--Repeat/Recurrent Myocardial Infarction

--Cardiac Rupture

--Papillary Muscle Rupture

--Ventricular Septal Perforation

--Cardiac Tamponade

--Cardiogenic Shock

--Cardiac Arrest

--Atrial Fibrillation or Flutter

--Ventricular Tachycardia/Ventricular Fibrillation

--Heart Failure (New Onset)

--Bleeding Events

--Hemorrhagic Shock

--Venous Thromboembolism (Pulmonary Embolism/Deep Vein Thrombosis)

--Peripheral Embolization

--Access Site Arteriovenous Fistula

--Ischemic Stroke (Cerebral Infarction/Thrombosis/Cerebral Embolism)

--Hemorrhagic Stroke (Cerebral Hemorrhage/Subarachnoid Hemorrhage)

--Stroke (Unspecified)

--Acute Renal Failure

--Dialysis (Hemodialysis/Peritoneal Dialysis)

--Infection (Nosocomial Infection)

--Contrast Reaction

**131 In-Hospital Device Placement**

【 **Every Item Has Three Options—Y/N/Unrecorded**】

--In-Hospital Implantation of Intra-Aortic Balloon Pump (IABP)

--In-Hospital Implantation of a Permanent Pacemaker

--In-Hospital Implantation of Implantable Cardioverter Defibrillator (ICD)

**132 Did the Patient Refuse the Following Treatment?**

【 **Every Item Has Three Options—Y/N/Unrecorded**】

--Refused Percutaneous Coronary Intervention (PCI)

--Refused Fibrinolysis

--Refused CABG

**133 Cardiac Arrest Date _____**【 **C**】

**134 Stroke Date _____**【 **C**】

**135.1 Bleeding Date _____**【 **C**】

**135.2 Bleeding Time _____**【 **C**】

**136 Location of Bleeding**【 **SC**】

--Unrecorded

--Access site

--Intracranial (Subdural)

--Intraocular

--Intraspinal

--Retroperitoneal

--Pericardial

--Gastrointestinal

--Genitourinary

--Other, Specify _____【 **F**】

**137 Interventions for Management of Bleeding**【 **SC**】

--Local Compression

--Open Surgery

--Endoscopic

--Transfusion

--Others

--None

--Unrecorded

**138 Hospital-Acquired Infection Site**【 **SC**】

--Pulmonary

--Genitourinary

--Gastrointestinal

--Skin

--Surgical site/Access site

--Other, Specify _____【 **F**】

--Unrecorded

**139 Documented Reasons for Non-prescription of the Following Agents in the First 24**

**Hours**【 **MC**】

--Aspirin

--Beta-blocker

--Fibrinolysis

--None of Above Is Recorded

**140 If Applicable, Specify the Documented Reason for Non-prescription of Aspirin in the**

**First 24 Hours _____**【 **F**】

**141 If Applicable, Specify the Documented Reason for Non-prescription of Beta-blocker in the First 24 Hours _____**【 **F**】

**142 If Applicable, Specify the Documented Reason for Non-prescription of Fibrinolysis in the First 24 Hours _____**【 **F**】

**143 Documented Reasons for Non-prescription of the Following Agents at Discharge**【

**MC**】

--Aspirin

--Angiotensin Converting Enzyme Inhibitors (ACE Inhibitor)

--Angiotensin Receptor Blocker (ARB)

--Beta-blocker

--Statin

--None of Above Is Recorded

**144 If Applicable, Specify the Documented Reason for Non-prescription of Aspirin at**

**Discharge _____**【 **F**】

**145 If Applicable, Specify the Documented Reason for Non-prescription of ACE Inhibitor at Discharge _____**【 **F**】

**146 If Applicable, Specify the Documented Reason for Non-prescription of ARB at**

**Discharge _____**【 **F**】

**147 If Applicable, Specify the Documented Reason for Non-prescription of Beta-blocker at Discharge _____**【 **F**】

**148 If Applicable, Specify the Documented Reason for Non-prescription of Statin at**

**Discharge _____**【 **F**】

**149 PCI Complication**

【 **Every Item Has Three Options—Y/N/Unrecorded**】

--Cardiac Tamponade

--Contrast Reaction

--Peripheral Embolization

--Access Site Arteriovenous Fistula

--Access Complication Requiring Surgery/Intervention

--Retroperitoneal Bleeding

**150 Stent Thrombosis after PCI**【 **SC**】

--Yes

--No

--Unrecorded

**151 Reasons for Repeat PCI**【 **SC**】

--Staged Procedure

--Ongoing or Recurrent Ischemia

--Other

--Unrecorded

**In-Hospital Device Placement**

**152 ICD**【 **SC**】

--Yes

--No

**153 ICD Date _____**【 **C**】

**154 IABP**【 **SC**】

--Yes

--No

**155 IABP Date _____**【 **C**】

**156 Pacemaker**【 **SC**】

--Yes

--No

**157 Pacemaker Date _____**【 **C**】

**Coronary Angiography**

**158 Did the Patient Undergo Diagnostic Catheterization or Diagnostic Coronary**

**Angiography?**【 **SC**】

--Yes

--No

**159.1 Date Patient Arrived at Catheterization Lab _____**【 **C**】

**159.2 Time Patient Arrived at Catheterization Lab _____**【 **F**】

**159.3 Time of Arterial Access_____**【 **C**】

**160 Coronary Angiography Access Site**【 **SC**】

--Radial artery

--Femoral artery

--Brachial artery

--Other

--Unrecorded

**161 Arterial Dominance**【 **SC**】

--Left Coronary Artery

--Right Coronary Artery

--Co-dominant

--Unrecorded

**162 Percent Stenosis Available**【 **MC**】

--Left Main artery (LM)

--Proximal LAD

--Mid/Distal LAD/Diagonal Branches

--Circ/OMs/LPDA and LPL Branches

--RCA/RPDA/RPL/AM Branches

--Ramus

--Unrecorded

**163 LM Stenosis Percent _____**【 **F**】

**164 Proximal LAD Stenosis Percent _____**【 **F**】

**165 Mid/Distal LAD/Diagonal Stenosis Percent _____**【 **F**】

**166 Circ/OMs/LPDA/LPL Stenosis Percent _____**【 **F**】

**167 RCA/PRDA/RPL/AM Stenosis Percent _____**【 **F**】

**168 Ramus Stenosis Percent _____**【 **F**】

**169 Was PCI Performed Immediately Following Diagnostic Catheterization?**【 **SC**】

--Yes

--No

**170 Closure Method**【 **SC**】

--Seal (Angioseal/Vasoseal)

--Suture

--Manual Compression

--Unrecorded

--Other, Specify _____ 【**F**】

**171 Was A Second Coronary Angiography Performed during Hospitalization?**【 **SC**】

--Yes

--No

【**172-183**】 **Repeat Questions No.159 to 171 for A Second Coronary Angiography**

**PCI & PTCA Reports**

**184 Is There a PCI Report?**【 **SC**】

--Yes

--No

**185 The Date of PCI _____**【 **C**】

**186 PCI Access Site**【 **SC**】

--Radial artery

--Femoral artery

--Brachial artery

--Other

--Unrecorded

**187 Did Guidewire Cross Lesion?**【 **SC**】

--Yes

--No

**188 Time of Guidewire Insertion _____**【 **F**】

**189 Time of the First Treatment of Lesion** (AngioJet/Other Thrombectomy/Aspiration

Device/Laser/Rotational Atherectomy) **_____**【 **F**】

**190 Time of The First Balloon Inflation______**【 **F**】

**191 Did the Patient Receive a Coronary Stent?**【 **SC**】

--Yes

--No

**192 Time of The First Stent Deployment _____**【 **F**】

**193.1 Brand Name of The First Stent _____**【 **D**】

**193.2 Brand Name of The Second Stent _____**【 **D**】

**193.3 Brand Name of The Third Stent _____**【 **D**】

**193.4 Brand Name of The Fourth Stent _____**【 **D**】

**193.5 Brand Name of The Fifth Stent _____**【 **D**】

**194 Type of Contrast Dye Used**【 **SC**】

--Urografin

--Iopamidol

--Iopromide

--Iohexol

--Iodixanol

--Iomeprol

--Ioversol

--Other

--Unrecorded

**195 Total Volume of Contrast Dye Used _____ml**【 **F**】

**196 Insertion of IABP during PCI procedure**【 **SC**】

--Yes

--No

--Unrecorded

**197 Closure Method**【 **SC**】

--Seal (Angioseal/Vasoseal)

--Suture

--Manual Compression

--Other method

--Unrecorded

**198 PCI Complication**

【 **Every Item Has Three Options—Y/N/Unrecorded**】

--Cardiac Tamponade

--Contrast Reaction

--Peripheral Embolization

--Access Site Arteriovenous Fistula

--Access Complication Requiring Surgery/Intervention

--Retroperitoneal Bleeding

**199 Stent Thrombosis after PCI**【 **SC**】

--Yes

--No

--Unrecorded

**200 Repeat PCI**【 **SC**】

--Yes

--No

**201 The Date of the Repeat PCI _____**【 **C**】

**202 Reasons for Repeat PCI**【 **SC**】

--Staged Procedure

--Ongoing or Recurrent ischemia

--Other

【**203-216**】 **Repeat Questions No.186 to 199 for a Second PCI**

**Nursing Records**

**217.1 Date Patient Arrived at Catheterization Lab For the First PCI_____**【 **C**】

**217.2 Time Patient Arrived at Catheterization Lab For the First PCI _____**【 **F**】

**217.3 Time of Arterial Access For the First PCI _____**【 **F**】

**218.1 Date Patient Arrived at Catheterization Lab For the Second PCI_____**【 **C**】

**218.2 Time Patient Arrived at Catheterization Lab For the Second PCI _____**【 **F**】

**218.3 Time of Arterial Access For the Second PCI _____**【 **F**】

**CABG Report**

**219 CABG during Hospitalization**【 **SC**】

--Yes

--No

**220.1 CABG Date______**【 **C**】

**220.2 CABG Time______**【 **F**】

**Imaging Examination**

**221 Was Chest X-ray or Other Lung Imaging (Chest CT/MRI) Performed?**【 **SC**】

--Yes

--No

**222 Chest X-ray/Other Lung Examination (Chest CT/MRI) Date _____**【 **C**】

**223 Chest X-ray/Other Lung Examination (Chest CT/MRI) Results**

【 **Every Item Has Two Options—Y/N**】

--Pulmonary Edema

--Pulmonary Demonstrable Infiltrate/Pneumonia

**224 Was a Second Chest X-ray or Other Lung Imaging (Chest CT/MRI) Performed?**【 **SC**

】

--Yes

--No

**225 The Second Chest X-ray/Other Lung Examination (Chest CT/MRI) Date _____**【 **C**】

**226 The Second Chest X-ray/Other Lung Examination (Chest CT/MRI) Results**

【 **Every Item Has Two Options—Y/N**】

--Pulmonary Edema

--Pulmonary Demonstrable Infiltrate/Pneumonia

**227 Was a Third Chest X-ray or Other Lung Imaging (Chest CT/MRI) Performed?**【 **SC**】

--Yes

--No

**228 The Third Chest X-ray/Other Lung Examination (Chest CT/MRI) Date _____**【 **C**】

**229 The Third Chest X-ray/Other Lung Examination (Chest CT/MRI) Results**

【 **Every Item Has Two Options—Y/N**】

--Pulmonary Edema

--Pulmonary Demonstrable Infiltrate/Pneumonia

**230 Was Echocardiography Performed?**【 **SC**】

--Yes

--No

**231 Echocardiography Date _____**【 **C**】

**232 Echocardiography LVEF Value _____%**【 **F**】

**233 Was Stress Test Performed?** 【 **SC**】

--Yes

--No

**234 Stress Test Date _____**【 **C**】

**235.1 Stress Test Type**【 **SC**】

--ECG only

--Radionuclide

--Echocardiography

--Cardiac MRI

--Unrecorded

**235.2 Method of Stress Test**【 **SC**】

--Exercise

--Pharmacologic

--Unrecorded

**236 Was Cardiac CT Angiogram Performed?**【 **SC**】

--Yes

--No

**237 Cardiac CT Angiogram Date _____**【 **C**】

**238 Ejection Fraction (EF) Value of Cardiac CT _____**%【**F**】

**239 Was a Multi-Gated Acquisition (MUGA) Scan Performed?**【 **SC**】

--Yes

--No

**240 MUGA Scan Ejection Fraction (EF) Value _____%**【 **F**】

**In-Hospital ECG**

**241 Original Interpretation of ECG**

【 **Every Item Has Three Options—Y/N/Unrecorded**】

--Acute MI

--Complete LBBB (Unspecified)

--ST-Elevation

--ST-Depression

--Previous MI(Q Wave)

--Ventricular Fibrillation

--Ventricular Tachycardia

--Atrial Fibrillation

--2nd Degree Atrioventricular Block Type 1

--2nd Degree Atrioventricular Block Type 2

--3nd Degree Atrioventricular Block

**Long-term Physician Orders**

**242 Name of Long-term Medication _____**【 **D**】

**243 Start Date and Time _____**【 **F**】

**Administration Method**【**SC**】

--P.O. /Undefined

--I.V. drip/ I.V. gtt (intravenously guttae)

--I.V.

--I.H. (hypodermic injection)

--I.D. (intradermal injection)

--Others

**Dose _____**【 **F**】

**Unit**【 **SC**】

--g

--mg

--ml

--U

--Piece/ #

--mg/kg

--mg/h

--mg/min

--mg/kg·h

--ml/kg

--ml/h

--ml/min

--ml/kg·min

--U/kg

--U/min

--U/h

--U/kg·h

--ug

--ug/kg

--ug/min

--ug/kg·min

--MU

--BU

--Other

--Unrecorded

**Frequency**【**SC**】

--Qd /QN

--Bid/q12h

--Tid/q8h

--Q6h

--As Needed

--Other

--Unrecorded

**End Date and Time _____**【 **F**】

【**244-401**】 **Repeat Questions No.242 to 243 for the Other 79 Available Agents**

**Short-term Physician Orders**

**402 Name of Short-term Medication _____**【 **D**】

**403 Start Date and Time _____**【 **F**】

**Administration**【**SC**】

--P.O. /Undefined

--I.V. drip/ I.V. gtt (intravenously guttae)

--I.V.

--I.H. (hypodermic injection)

--I.D. (intradermal injection)

--Others

**Dose_____**【 **F**】

**Unit**【 **SC**】

--g

--mg

--ml

--U

--Piece #

--mg/kg

--mg/h

--mg/min

--mg/kg·h

--ml/kg

--ml/h

--ml/min

--ml/kg·min

--U/kg

--U/min

--U/h

--U/kg·h

--ug

--ug/kg

--ug/min

--ug/kg·min

--MU

--BU

--Other

--Unrecorded

**Frequency**【**SC**】

--Qd /QN

--Bid/q12h

--Tid/q8h

--Q6h

--As Needed

--Other

--Unrecorded

【**404-601**】 **Repeat Questions No.402 to 403 for the Other 99 Available Agents**

**Discharge Medications**

**602 Discharge Agent _____**【 **D**】

**603 Administration Method**【**SC**】

--P.O. /Undefined

--I.V. drip/ I.V. gtt (intravenously guttae)

--I.V.

--I.H. (hypodermic injection)

--I.D. (intradermal injection)

--Others

**Dose _____**【 **F**】

**Unit _____**【 **SC**】

--g

--mg

--ml

--U

--Piece/ #

--mg/kg

--mg/h

--mg/min

--mg/kg·h

--ml/kg

--ml/h

--ml/min

--ml/kg·min

--U/kg

--U/min

--U/h

--U/kg·h

--ug

--ug/kg

--ug/min

--ug/kg·min

--MU

--BU

--Other

--Unrecorded

**Frequency**【**SC**】

--Qd /QN

--Bid/q12h

--Tid/q8h

--Q6h

--As Needed

--Others

--Unrecorded

【**604-631**】 **Repeat Questions No.602 to 603 for the Other 14 Available Agents**

**Temperature Report**

**632 Height _____ cm**【 **F**】

**633 Admission Weight _____ kg**【 **F**】

**634 Study ID _____**【 **F**】

**CHINA PEACE-RETROSPECTIVE AMI STUDY CASE REPORT FORM, PART 2**

**Contents**

Medical Record Face Sheet ........................................ **Error! Bookmark not defined.**

Lab Test ..................................................................................................................... 46

**1 Study ID _____**【 **Free Text (F)**】

**Medical Record Face Sheet**

**2 Medical Record Number _____**【 **F**】

**3 Medical Insurance**【 **Single Choice (SC)**】

--Self-pay/No insurance

--Free Medical Care

--Urban Residents/Worker Medical Insurance

--Comprehensive Arrangement for Serious Disease

--Farmer Medical Insurance

--Poverty Assistance Insurance

--Business Insurance

--Other Social Insurance

--Unrecorded

**4 Gender**【 **SC**】

--Male

--Female

**5 Age _____**【**F**】

**6 Marital Status**【 **SC**】

--Married

--Single

--Divorced

--Widowed

--Unrecorded

**7 Occupation**【 **SC**】

--Retired

--Worker (Industrial/Mining/Construction/Other Similar Type of Work)

--Farmer

--Office work

--Cadre

--Teacher

--Student

--Physician

--Businessman

--Engineer

--Policeman or Soldier

--Freelancers

--Do Not Work

--Other

--Unrecorded

**8 Ethnicity**【 **SC**】

--Han

--Zhuang

--Man

--Hui

--Miao

--Weiwuer

--Tujia

--Yi

--Menggu

--Zang

--Buyi

--Other

--Unrecorded

**9 Nationality**【 **SC**】

--China

--Other

--Unrecorded

**10 Location of Residence**【 **SC**】

--Urban

--Rural

--Overseas

--Unrecorded

**11 Postal Code of Residence _____**【 **F**】

**12 Admission Date _____**【 **C**】

**13 Admission Time _____**【 **F**】

**14 The Department of Admission**【 **SC**】

--Cardiovascular Department

--Internal Medicine Department

--Geriatrics

--Cadre's ward

--Other

--Unrecorded

**15 Was Patient Transferred from One Department to Another?**【 **SC**】

--Yes

--No

--Unrecorded

**16 Discharge Department**【 **SC**】

--Cardiovascular Department

--Internal Medicine Department

--Geriatrics

--Cadre's Ward

--Other

--Unrecorded

**17 The Length of Hospitalization _____ Days**【 **F**】

**18 Date Diagnosis Was Confirmed ______**【 **C**】

**19 Admission Diagnoses (Related to Coronary Heart Disease)**

【 **Multiple Choices Permitted (MC)**】

--Coronary Heart Disease

--Acute Coronary Syndrome

--Acute Myocardial Infarction

--Acute Extensive Anterior Myocardial Infarction

--Acute Anterior Myocardial Infarction

--Acute Septal Myocardial Infarction

--Acute Inferior Myocardial Infarction

--Acute Lateral Myocardial Infarction

--Acute Posterior Myocardial Infarction

--Acute Right Ventricular Myocardial Infarction

--Acute Non-ST-Elevation Myocardial Infarction

--Acute ST-Elevation Myocardial Infarction

--Subendocardial Myocardial Infarction

--Suspected Acute Myocardial Infarction

--Previous Q-wave Myocardial Infarction

--Unstable Angina Pectoris

--Stable Angina Pectoris

--Prinzmetal's Angina

--Angina (Unrecorded Subtype)

--None of the Above Is Recorded

**20 Admission Diagnoses (Unrelated to Coronary Heart Disease)**【 **MC**】

--Cardiac Arrest

--Cardiogenic Shock

--Ventricular Fibrillation/Ventricular Tachycardia

--Heart Failure

--Hemorrhagic Stroke (Cerebral Hemorrhage/Subarachnoid Hemorrhage)

--Ischemic Stroke (Cerebral Infarction/Cerebral Embolism/Cerebral Thrombosis)

--Stroke (Unspecified)

--Pneumonia

--COPD Exacerbation

--Acute Renal Failure

--Chronic Renal Failure

--Dialysis (Hemodialysis/Peritoneal Dialysis)

--Dyslipidemia

--Hypertension

--Diabetes Mellitus

--Trauma

--Hepatitis

--Cirrhosis

**21 Discharge Diagnoses**【 **MC**】

--Coronary Heart Disease

--Acute Coronary Syndrome

--Acute Myocardial Infarction

--Acute Extensive Anterior Myocardial Infarction

--Acute Anterior Myocardial Infarction

--Acute Septal Myocardial Infarction

--Acute Inferior Myocardial Infarction

--Acute Lateral Myocardial Infarction

--Acute Posterior Myocardial Infarction

--Acute Right Ventricular Myocardial Infarction

--Acute Non ST-Elevation Myocardial Infarction

--Acute ST-Elevation Myocardial Infarction

--Subendocardial Myocardial Infarction

--Acute Myocardial Infarction Suspected

--Previous Q-Wave Myocardial Infarction

--Unstable Angina Pectoris

--Stable Angina Pectoris

--Prinzmetal's Angina

--Angina (Unrecorded Subtype)

--Repeated/Recurrent Myocardial Infarction

--Repeated/Recurrent Unstable Angina

--Died

--Cardiac Rupture

--Cardiac Tamponade

--Cardiogenic Shock

--Cardiac Arrest

--Atrial Fibrillation or Flutter

--Ventricular Tachycardia/Ventricular Fibrillation

--Heart Failure (New Onset)

--Bleeding

--Hemorrhagic Shock

--Venous Thromboembolism

--Pulmonary Embolism

--Deep Vein Thrombosis

--Peripheral Embolization

--Access Site Arteriovenous Fistula

--Ischemic Stroke (Cerebral Infarction/Thrombosis/Cerebral Embolism)

--Hemorrhagic Stroke (Cerebral Hemorrhage/Subarachnoid Hemorrhage)

--Stroke (Unspecified)

--Pneumonia

--COPD Exacerbation

--Acute Renal Failure

--Chronic Renal Failure

--Dialysis (Hemodialysis/Peritoneal Dialysis)

--Infection (Nosocomial Infection)

--Septicemia

--Contrast Reaction

--Dyslipidemia

--Hypertension

--Diabetes Mellitus

--Trauma

--Hepatitis

--Cirrhosis

**22 ICD (for Every Diagnosis)**【 **SC**】

--ICD-9

--ICD-10

--Unrecorded

**23 ICD-9 (Specify) _____**【 **F**】

**24 ICD-10 (Specify) _____**【 **F**】

**25 Nosocomial Infection (Specify) _____**【 **F**】

**26 Drug Allergy (Specify) _____**【 **F**】

**27 HBs-Ag**【 **SC**】

--Untested

--Negative

--Positive

**28 HCV-Ab**【 **SC**】

--Untested

--Negative

--Positive

**29 HIV-Ab**【 **SC**】

--Untested

--Negative

--Positive

**30 Procedure Date _____**【 **F**】

**31 Procedures Performed during Hospitalization**【 **MC**】

--Percutaneous Coronary Intervention (PCI)

--Coronary Angiography (CAG)

--PCI+CAG

--Percutaneous Transluminal Coronary Angioplasty (PTCA)

--Coronary Artery Bypass Graft Surgery (CABG)

--Intracranial Decompression

--Intra-aortic Balloon Pump Placement (IABP)

--Extra Corporeal Membrane Oxygenation (ECMO)

--Left Ventricular Assist Device (LVAD)

--Pacemaker

--Angiostomy

--Other (Specify) ______【 **F**】

**32 Gross Charge _____ Yuan**【 **F**】

**33 Autopsy**【 **SC**】

--Yes

--No

--Unrecorded

**34 The Type of Blood Transfusion**【 **MC**】

--No Blood Transfusion

--Red Blood Cell

--Platelet

--Blood Plasma

--Whole Blood

--Other (Specify)______ 【 **F**】

**Lab Test**

**35 Unit of Myohemoglobin**【 **SC**】

--IU/L

--ng/ml

--mg/ml

--Other

**36 Myohemoglobin Upper Limit of Normal (ULN) _____**【 **F**】

**37 Initial Myohemoglobin Value _____**【 **F**】

**38 Initial Myohemoglobin Date and Time _____**【 **C**】

**39 Maximum Myohemoglobin _____**【 **F**】

**40 Maximum Myohemoglobin Date and Time _____**【 **C**】

**41 Unit of Creatine Kinase (CK) Level**【 **SC**】

--IU/L

--ng/ml

--mg/ml

--%

--mg/IU

--ml/IU

**42 Initial CK Upper Limit of Normal (ULN) _____**【 **F**】

**43 Initial Creatine Kinase (CK) Value _____**【 **F**】

**44 Initial Creatine Kinase (CK) Date and Time _____**【 **C**】

**45 Maximum Creatine Kinase (CK) Value _____**【 **F**】

**46 Maximum Creatine Kinase (CK) Date and Time _____**【 **C**】

**47 Unit of CKMB Level**【 **SC**】

--IU/L

--ng/ml

--mg/ml

--%

--mg/IU

--ml/IU

**48 Initial CK Upper Limit of Normal (ULN) _____**【 **F**】

**49 Initial CK-MB Value _____**【 **F**】

**50 Initial CK-MB Date and Time _____**【 **F**】

**51 Unit of CKMB**【 **SC**】

--IU/L

--ng/ml

--mg/ml

--%

--mg/IU

--ml/IU

**52 Maximum CK-MB Value _____**【 **F**】

**53 Maximum CKMB Date and Time _____**【 **C**】

**54 Unit of Troponin I**【 **SC**】

--ng/ml

--Other

**55 Initial Troponin I Value**【 **SC**】

--Positive

--Negative

--Trace (+/-)

--Numerical Value

**56 Initial Troponin I Upper Reference Limit (URL) _____**【 **F**】

**57 Initial Troponin I Value _____**【 **F**】

**58 Initial Troponin I Date and Time _____**【 **C**】

**59 Maximum Troponin I Value _____**【 **F**】

**60 Maximum Troponin I Date and Time _____**【 **C**】

**61 Unit of Troponin T**【 **SC**】

--ng/ml

--Other

**62 Initial Troponin T Value**【 **SC**】

--Positive

--Negative

--Numerical Value

**63 Initial Troponin T Upper Reference Limit (URL) _____**【 **F**】

**64 Initial Troponin T Value _____**【 **F**】

**65 Initial Troponin T Date and Time _____**【 **C**】

**66 Maximum Troponin T value _____**【 **F**】

**67 Maximum Troponin T Date and Time _____**【 **C**】

**68 Unit of Troponin (Unspecified)** 【 **SC**】

--ng/ml

--Other

**69 Initial Troponin (Unspecified) Value**【 **SC**】

--Positive

--Negative

--Numerical Value

**70 Initial Troponin (Unspecified) Upper Reference Limit (URL) _____**【 **F**】

**71 Initial Troponin (Unspecified) Value _____**【 **F**】

**72 Initial Troponin (Unspecified) Date and Time _____**【 **C**】

**73 Maximum Troponin (Unspecified) Value _____**【 **F**】

**74 Maximum Troponin (Unspecified) Date and Time _____**【 **C**】

**75 First White Blood Cell (WBC) Value _____ X 109**【 **F**】

**76 Date and Time _____**【 **C**】

**77 Initial Neutrophil Ratio _____ %**【 **F**】

**78 Date and Time _____**【 **C**】

**79 Initial Hemoglobin Value _____ g/L**【 **F**】

**80 Date and Time**

**_____**【 **C**】

**81 Initial Platelet Count Value _____ x109**【 **F**】

**82 Initial Hematocrit Value _____**【 **F**】

**83 Date and Time _____**【 **C**】

**84 Minimum Platelet Count Value _____x109**【 **F**】

**85 Date and Time**

**_____**【 **C**】

**86 Lowest Recorded Hemoglobin Value _____g/L**【 **F**】

**87 Date and Time _____**【 **C**】

**88 Last Hemoglobin Value _____g/L**【 **F**】

**89 Date and Time _____**【 **C**】

**90 Minimum Hematocrit Value _____**【 **F**】

**91 Date and Time _____**【 **C**】

**92 Last Hematocrit Value _____**【 **F**】

**93 Initial Urine Routine Date and Time _____**【 **C**】

**94 Urine Protein**【 **SC**】

--Negative

--Positive/+

--Trace（+/-）

--++

--+++

--++++

**95 Initial LDH Value _____ (U/L; IU/L)**【 **F**】

**96 Date and Time _____**【 **C**】

**97 Units of Glucose**【 **SC**】

--mg/dl

--mmol/L

--Other

**98 Initial Glucose Value _____**【 **F**】

**99 Initial ALT Value _____U/L**【 **F**】

**100 Date and Time _____**【 **C**】

**101 Initial AST Value _____U/L**【 **F**】

**102 Date and Time _____**【 **C**】

**103 Maximum ALT _____U/L**【 **F**】

**104 Date and Time _____**【 **C**】

**105 Maximum AST _____U/L**【 **F**】

**106 Date and Time _____**【 **C**】

**107 Lowest ALT _____U/L**【 **F**】

**108 Date and Time _____**【 **C**】

**109 Lowest AST _____U/L**【 **F**】

**110 Date and Time _____**【 **C**】

**111 Initial Total Bilirubin Value _____umol/L**【 **F**】

**112 Date and Time _____**【 **C**】

**113 Initial Direct Bilirubin Value _____umol/L**【 **F**】

**114 Date and Time _____**【 **C**】

**115 Units of Creatinine**【 **SC**】

--mg/dl

--mmol/L

--Other

**116 Initial Creatinine Value _____**【 **F**】

**117 Date and Time _____**【 **C**】

**118 Maximum Creatinine Value _____**【 **F**】

**119 Date and Time _____**【 **C**】

**120 Last Creatinine Value _____**【 **F**】

**121 Date and Time _____**【 **C**】

**122 Units of BUN**【 **SC**】

--mg/dl

--mmol/L

--Other

**123 Initial BUN Value _____**【 **F**】

**124 Date and Time _____**【 **C**】

**125 Maximum BUN Value _____**【 **F**】

**126 Date and Time _____**【 **C**】

**127 Units of Lipid**【 **SC**】

--mg/dl

--mmol/L

--Other

**128 Initial Lipid Date and Time _____**【 **C**】

**129 Total Cholesterol Value _____**【 **F**】

**130 HDL Cholesterol Value _____**【 **F**】

**131 LDL Cholesterol Value _____**【 **F**】

**132 Triglycerides Value _____**【 **F**】

**133 Initial Potassium Value _____ (mEq/L; mmol/L)**【 **F**】

**134 Date and Time _____**【 **C**】

**135 Last Potassium Value _____(mEq/L; mmol/L)**【 **F**】

**136 Date and Time _____**【 **C**】

**137 Units of BNP/NT-pBNP**【 **SC**】

--pg/ml

--ug/L

--ug/ml

--Fmol/L

--Other

**138 Initial BNP Value _____**【 **F**】

**139 Date and Time _____**【 **C**】

**140 Initial NT-pBNP Value _____**【 **F**】

**141 Date and Time _____**【 **C**】

**142 Units of CRP**【 **SC**】

--mg/L

--pg/ml

--Other

**143 Initial CRP Value_____**【 **F**】

**144 Units of HsCRP**【 **SC**】

--mg/L

--pg/ml

--Other

**145 Initial HsCRP Value _____**【 **F**】

**146 Initial Coagulation Examinations Date and Time _____**【 **C**】

**147 INR Value _____**【 **F**】

**148 APTT Value _____ s**【 **F**】

**149 Initial PT Value _____s**【 **F**】

**150 The Second Coagulation Examinations Date and Time _____**【 **C**】

**151 INR Value _____**【 **F**】

**152 APTT Value _____s**【 **F**】

**153 Initial PT Value _____s**【 **F**】

**154 The Third Coagulation Examinations Date and Time _____**【 **C**】

**155 INR Value _____**【 **F**】

**156 APTT Value _____s**【 **F**】

**157 Initial PT Value _____s**【 **F**】

**158 The Fourth Coagulation Examinations Date and Time _____**【 **C**】

**159 INR Value _____**【 **F**】

**160 APTT Value _____s**【 **F**】

**161 Initial PT Value _____ s**【 **F**】

**162 The Fifth Coagulation Examinations Date and Time _____**【 **C**】

**163 INR Value _____**【 **F**】

**164 APTT Value _____s**【 **F**】

**165 Initial PT Value _____ s**【 **F**】

**166 The Sixth Coagulation Examinations Date and Time _____**【 **C**】

**167 INR Value _____**【 **F**】

**168 APTT Value _____s**【 **F**】

**169 Initial PT Value _____s**【 **F**】

**170 HBsAg**【 **SC**】

--Positive

--Negative

--Numerical Value _____【 **F**】

**171 HBsAg Upper Reference Limit (URL) _____**【 **F**】

**172 Anti-HBs/HBs-Ab**【 **SC**】

--Positive

--Negative

--Numerical Value _____【 **F**】

**173 Anti-HBs/HBs-Ab Upper Reference Limit (URL) _____**【 **F**】

**174 HBeAg**【 **SC**】

--Positive

--Negative

--Numerical Value _____【 **F**】

**175 HBeAg Upper Reference Limit (URL) _____**【 **F**】

**176 Anti-HBeAg/HBe-Ab**【 **SC**】

--Positive

--Negative

--Numerical Value _____【 **F**】

**177 Anti-HBeAg/HBe-Ab Upper Reference Limit (URL) _____**【 **F**】

**178 Anti-HBcAg/HBc-Ab**【 **SC**】

--Positive

--Negative

--Numerical Value _____【**F**】

**179 Anti-HBcAg/HBc-Ab Upper Reference Limit (URL) _____**【 **F**】

**180 PreS1**【 **SC**】

--Positive

--Negative

--Numerical Value _____【 **F**】

**181 PreS1 Upper Reference Limit (URL) _____**【 **F**】

**182 HCV-Ab (Unspecified)**【 **SC**】

--Positive

--Negative

--Numerical Value _____【 **F**】

**183 HCV-Ab (Unspecified) Upper Reference Limit (URL) _____**【 **F**】

**184 HCV IgG**【 **SC**】

--Positive

--Negative

--Numerical Value _____【 **F**】

**185 HCV IgG Upper Reference Limit (URL) _____**【 **F**】

**186 HCV IgM**【 **SC**】

--Positive

--Negative

--Numerical Value _____【 **F**】

**187 HCV IgM Upper Reference Limit (URL) _____**【 **F**】

**CHINA PEACE-RETROSPECTIVE AMI STUDY DATA DICTIONARY**

**Study ID**

Coding Instructions: Indicate the identification number for the study.

**Medical Record Number**

Coding Instructions: Indicate the identification number for this record.

**Hospital Number**

Coding Instructions: Indicate the identification number for this hospital.

**Are Daily Records Filed into This Medical Record?**

Coding Instructions: Indicate if there are daily records available as a part of this medical record

Target Value: N/A Selections: (1) No (2) Yes

Supporting Definition: Availability of daily progress notes with medical record

Source: Definition per CORE team

**Gender**

Coding Instructions: Indicate the gender of the patient. Selections: (1) Male (2) Female (3) Unrecorded

**Age**

Coding Instructions: Indicate the age of the patient in years.

**Medical Insurance**

Coding Instructions: Indicate the patient’s medical insurance status. Target Value: N/A

Selections:

Urban residents/worker medical insurance

Farmer medical insurance

Business insurance

Comprehensive arrangement for serious disease

Poverty assistance insurance

Other social insurance

Free medical care

Self-pay/no insurance

Unrecorded

Supporting Definitions:

Urban residents/worker medical insurance: the medical insurance provided by the government for people who live in urban areas; also is the type of insurance provided by a company or factory for their employees.

Farmer medical insurance: a new medical insurance type funded by the government for farmers.

Business insurance: Insurance paid for by individuals to insurance companies. Comprehensive arrangement for serious disease: Insurance for people living in urban areas who are employed by a company or factory. Also may apply to persons who are retired. Specifications vary by city. Covered diseases are different than that covered by urban residents/worker medical insurance.

Poverty assistance insurance: Medical aid provided by the government for poverty-

stricken individuals to cover a portion of expenses

Free medical care: The government provides free medical treatment for a specific group of people such as for disabled members of the armed forces.

Self-pay/no insurance: Indicated for those without medical insurance or for whom certain

diseases are not covered by insurance. Patient will pay for these costs.

Patients may have more than one insurance type at a given time. Abstractor should select all that apply

Source: Definition per China team.

**Height**

Coding Instructions: Indicate the patient’s height in centimeters

Note(s): Measurement from the transferring facility is acceptable

Target Value: The first value between arrival at this facility and discharge

Selections: (none)

Supporting Definitions: (none)

Source: Adapted from AHA Get with the Guidelines ACTION registry

**Admission Weight**

Coding Instructions: Indicate he patient’s weight in kilograms

Note(s): Measurement from the transferring facility is acceptable

Target Value: The first value between first medical contact and discharge

Selections: (none)

Supporting Definitions: (none)

Source: Adapted from AHA Get with the Guidelines ACTION registry

**Marital Status**

Coding Instructions: Indicate the marital status of the patient

Target Value: N/A Selections:

Married Single Divorced Widowed Unrecorded

Supporting Definitions:

Married – A formal union with a person of the opposite sex, typically as recognized by law, by which they are considered husband and wife.

Single- Never been married.

Divorced – Previous marriage has been legally dissolved by a court or another legal body. Widowed- A person who has lost his/her spouse to death and has not remarried.

Source: Definition per CORE team; Oxford English Dictionary

**Occupation**

Coding Instructions: Indicate the occupation of the patient

Target Value: N/A Selections:

Retired

Worker (Industrial/Mining/Construction/Other Similar Type of Work)

Farmer Office work Cadre

Teacher Student Physician Businessman Engineer

Policeman or Soldier Freelancers Unemployed

Other

Unrecorded

Supporting Definitions: Documentation of occupation by treating physician

Source: Definition per CORE team; China team

**Ethnicity**

Coding Instructions: Indicate the ethnicity of the patient

Target Value: N/A Selections:

Han

Zhuang

Man Hui Miao

Weiwuer

Tujia Yi Menggu Zang Buyi Other

Unrecorded

Supporting Definitions: The highlighted groups comprise the top 11 ethnicities by population size among 56 ethnic groups within China.

Source: Definition per CORE team; China team

**Nationality**

Coding Instructions: Indicate the nationality of the patient

Target Value: N/A Selections:

China Other Unrecorded

Supporting Definition: Nationality is the status of belonging to a nation by origin, birth, or naturalization

Source: Definition per CORE team; Oxford English Dictionary

**Location of Residence**

Coding Instructions: Indicate the patient’s location of residence based on the supporting definition.

Target Value: The value on arrival to facility

Selections: (1) Urban (2) Rural (3) Overseas (4) Unrecorded

Supporting Definitions:

Urban residence includes the areas of a city or municipality or Hong Kong, Macao and Taiwan regions.

Rural residence includes the areas belonging to the countryside including associated townships and villages.

Source: Definition per China team

**Postal Code of Residence**

Coding Instructions: Indicate patient’s postal code of primary residence

Target Value: The value on arrival to facility

Selections: (none)

Supporting Definitions: (none) Source: Definition per China team

**Date of Arrival at Your Hospital**

Coding Instructions: Indicate the date the patient arrived at your facility. Target Value: N/A

Selections: (none)

Supporting Definitions: (none)

Source: Adapted from AHA Get with the Guidelines ACTION registry

**Time of Arrival at Your Hospital**

Coding Instructions: Indicate the time the patient arrived at your facility. Target Value: N/A

Selections: (none)

Supporting Definitions: (none)

Source: Adapted from AHA Get with the Guidelines ACTION registry

**Was Time of Arrival at Your Hospital Estimated?**

Coding Instructions: Indicate if the time the patient arrived at your facility was uncertain and therefore estimated.

Target Value: N/A

Selections: (1) Yes (2) No (3) Unrecorded

Supporting Definitions: (none)

Source: Adapted from AHA Get with the Guidelines ACTION registry

**Admission Date**

Coding Instructions: Indicate the date the patient was admitted to your facility

Target Value: N/A Selections: (none)

Supporting Definitions: (none) Source: Definition per CORE team

**Admission Time**

Coding Instructions: Indicate the time the patient was admitted to your facility

Target Value: N/A Selections: (none)

Supporting Definitions: (none) Source: Definition per CORE team

**Department of Admission**

Coding Instructions: Indicate the department responsible for the patient admission. Target Value: N/A

Selections: (1) Cardiovascular Department (2) Internal Medicine Department (3) Geriatrics (4)

Cadre’s Ward (5) Other (6) Unrecorded

Supporting Definitions: (none) Source: Definition per China team

**Patient Transferred from One Department to Another**

Coding Instructions: Indicate if the patient was transferred to another department after initial admission.

Target Value: N/A

Selections: (1) Yes (2) No (3) Unrecorded

Supporting Definitions: (none) Source: Definition per China team

**Discharge Department**

Coding Instructions: Indicate the department responsible for the patient’s discharge. Target Value: N/A

Selections: (1) Cardiovascular Department (2) Internal Medicine Department (3) Geriatrics (4) Cadre’s Ward (5) Other (6) Unrecorded

Supporting Definitions: (none) Source: Definition per China team

**Length of Hospitalization**

Coding Instructions: Indicate the duration of the patient’s stay in the hospital in days. Target Value: The duration between date of admission and date of discharge. Selections: N/A

Supporting Definitions: (none) Source: Definition per CORE team

**Date Diagnosis was Confirmed**

Coding Instructions: Indicate the date on which the diagnosis was confirmed. Target Value: N/A

Selections: N/A

Supporting Definitions: (none) Source: Definition per CORE team

**Means of Transport to First Facility**

Coding Instructions: Indicate the means of transportation to the facility where the patient first received treatment.

Target Value: N/A

Selections: (1) Self/Family (2) Ambulance (3) Other (4) Unrecorded

Supporting Definitions: (none)

Source: Adapted from AHA Get with the Guidelines ACTION registry

**Date When Patient Was Taken By Ambulance to the Hospital**

Coding Instructions: Indicate the date when the patient was first taken by ambulance to your facility.

Target Value: N/A Selections: (none)

Supporting Definitions: (none)

Source: Adapted from AHA Get with the Guidelines ACTION registry

**Time When Patient Was Taken By Ambulance to the Hospital**

Coding Instructions: Indicate the time when the patient was first taken by ambulance to your facility.

Target Value: N/A Selections: (none)

Supporting Definitions: (none)

Source: Adapted from AHA Get with the Guidelines ACTION registry

**Was the Time the Patient was Taken by Ambulance to Hospital Estimated?**

Coding Instructions: Indicate if the time when the patient was first taken by ambulance to your facility was not explicitly written in the medical chart and was therefore estimated by the abstractor.

Target Value: N/A Selections: (1) No (2) Yes Supporting Definitions: (none)

Source: Adapted from AHA Get with the Guidelines ACTION registry

**Did The Patient Receive Medical Assistance at an Outside Facility Prior to Arrival?**

Coding Instructions: Indicate if the patient received medical assistance at an outside facility prior to arrival.

Target Value: N/A

Selections: (1) No (2) Yes

Supporting Definitions: (none)

Source: Adapted from AHA Get with the Guidelines ACTION registry

**Means of Transfer**

Coding Instructions: Indicate the means of transportation from the outside facility to your facility. Target Value: The last value between transfer from outside facility and arrival at this facility Selections: (1) Ambulance (2) Air (3) Other (4) Unrecorded

Supporting Definitions: (none)

Source: Adapted from AHA Get with the Guidelines ACTION registry

**Date of Arrival at Outside Facility**

Coding Instructions: Indicate the date the patient arrived at the outside facility. Target Value: N/A

Selections: (none)

Supporting Definitions: (none)

Source: Adapted from AHA Get with the Guidelines ACTION registry

**Time of Arrival at Outside Facility**

Coding Instructions: Indicate the time the patient arrived at the outside facility. Target Value: N/A

Selections: (none)

Supporting Definitions: (none)

Source: Adapted from AHA Get with the Guidelines ACTION registry

**Was the Time of Arrival at Outside Facility Estimated?**

Coding Instructions: Indicate if the time the patient arrived at the outside facility was not explicitly written in the medical chart and was therefore estimated by the abstractor.

Target Value: N/A Selections: (1) No (2) Yes Supporting Definitions: (none)

Source: Adapted from AHA Get with the Guidelines ACTION registry

**Transfer from Outside Facility Date**

Coding Instructions: Indicate the date the patient left the outside facility. Target Value: N/A

Selections: (none)

Supporting Definitions: (none)

Source: Adapted from AHA Get with the Guidelines ACTION registry

**Transfer from Outside Facility Time**

Coding Instructions: Indicate the time the patient left the outside facility. Target Value: N/A

Selections: (none)

Supporting Definitions: (none)

Source: Adapted from AHA Get with the Guidelines ACTION registry

**Was the Time of Transfer from Outside Facility Estimated?**

Coding Instructions: Indicate if the time the patient left the outside facility was estimated. Target Value: N/A

Selections: (1) No (2) Yes

Supporting Definitions: (none)

Source: Adapted from AHA Get with the Guidelines ACTION registry

**Name of Transferring Facility**

Coding Instructions: Indicate the name of the facility from which the patient was transferred. Target Value: N/A

Selections: (none)

Supporting Definitions: (none)

Source: Adapted from AHA Get with the Guidelines ACTION registry

**Gross Charge of Hospitalization in Yuan**

Coding Instructions: Indicate total charge for patient hospitalization

Target Value: N/A Selections: (none)

Supporting Definitions: The entire charge associated with the hospitalization in Yuan*.*

Source: Definition per China team

**Time since Symptom Onset**

Coding Instructions: How much time has passed since onset of ischemic symptoms? Target Value: N/A

Selections: (1) ___ Days (2) ___Hours

Supporting Definitions: Indicate the time since the recent ischemic episode started. Source: Definition per CORE team; China team

**Time of Symptom Onset (precise)**

Coding Instructions: Indicate the time the patient first noted ischemic symptoms that lasted greater than or equal to 10 minutes.

If an estimated symptom onset time is recorded, code 'symptom onset time estimated'.

Target Value: The first time symptoms lasted greater than 10 minutes on the symptom onset date

Selections: (none)

Supporting Definitions: (none)

Note: If the symptom onset time is not specified in the medical record, it may be recorded as

0700 for morning; 1200 for lunchtime; 1500 for afternoon; 1800 for dinnertime; 2200 for evening and 0300 if awakened from sleep.

Source: Adapted from AHA Get with the Guidelines ACTION registry

**Was the Symptom Onset Time Estimated?**

Coding Instructions: Indicate if the symptom onset time was estimated. Target Value: N/A

Selections: (1) No (2) Yes

Supporting Definitions: (none)

Source: Adapted from AHA Get with the Guidelines ACTION registry

**Patient Chest Discomfort**

Coding Instructions: Did the patient experience chest symptoms including chest pain, chest discomfort, chest pressure, or other chest symptoms?

Target Value: N/A

Selections: (1) No (2) Yes

Supporting Definitions: (none)

Source: Definition per CORE team, adapted from the AHA Get with the Guidelines ACTION

registry

**Did the Chest Discomfort Last 10 or More Minutes?**

Coding Instructions: Did the patient’s chest discomfort last 10 or more minutes? Target Value: N/A

Selections: (1) Yes (2) No (3) Unrecorded

Supporting Definitions: (none)

Source: Definition per CORE team; China team

**Did the Patient Have Ischemic Symptoms Other than Chest Discomfort?**

Coding Instructions: Indicate if there is physician documentation of other ischemic symptoms. Target Value: N/A

Selections: (1) No (2) Yes

Supporting Definitions: Documentation of one or more of these symptoms warrants a “Yes”

answer: shortness of breath, pain at non-chest sites, nausea, vomiting, or fatigue

Source: Definition per CORE team

**Did Other Ischemic Symptoms Last 10 or More Minutes?**

Coding Instructions: Did the patient’s other ischemic symptoms last 10 or more minutes? Target Value: N/A

Selections: (1) Yes (2) No (3) Unrecorded

Supporting Definitions: (none)

Source: Definition per CORE team; China team

**Admission Diagnosis (Related to Coronary Heart Disease)**

Coding Instructions: Indicate the admission diagnosis related to coronary heart disease documented in the medical record.

Note(s): Mark as many of the choices that apply.

Selections: Coronary Heart Disease, Acute Coronary Syndrome, Acute Myocardial Infarction, Acute Extensive Anterior Myocardial Infarction, Acute Anterior Myocardial Infarction, Acute Septal Myocardial Infarction, Acute Inferior Myocardial Infarction, Acute Lateral Myocardial Infarction, Acute Posterior Myocardial Infarction, Acute Right Ventricular Myocardial Infarction, Acute Non-ST-Elevation Myocardial Infarction, Acute ST-Elevation Myocardial Infarction, Subendocardial Myocardial Infarction, Acute Myocardial Infarction Suspected, Previous Q-wave Myocardial Infarction, Unstable Angina Pectoris

(Variant Angina Pectoris, Resting Angina, Supine Angina, Worsening Angina, Post-Infarction Angina), Stable Angina Pectoris, Prinzmetal’s Angina, Angina (Unrecorded Subtype) Supporting Definition: If none of the above options is noted in the chart, mark “None of the above is recorded”.

Source: Definition per CORE team

**Admission Diagnosis (Unrelated to Coronary Heart Disease)**

Coding Instructions: Indicate the admission diagnosis unrelated to coronary heart disease documented in the medical record.

Note(s): Mark as many of the choices that apply.

Selections: Cardiac Arrest, Cardiogenic Shock, Heart Failure, Ventricular Fibrillation/Ventricular Tachycardia, Hypertension, Dyslipidemia, Diabetes Mellitus, Diabetic Nephropathy, Hemorrhagic Stroke, Ischemic Stroke, Stroke (Unspecified), Pneumonia, COPD Exacerbation, Gastrointestinal Bleeding, Hepatitis, Cirrhosis, Chronic Renal Failure, Acute Renal Failure, Dialysis, Trauma.

Supporting Definition: If none of the above options is noted in the chart, mark “None of the

above is recorded”.

Source: Definition per CORE team

**Antiplatelet Therapy Prior to Arrival**

Coding Instructions: Indicate whether the patient was given aspirin, clopidogrel, ticlopidine or other antiplatelet therapy by a health provider (EMS, transferring hospital personnel, etc.) prior to arrival at this hospital or if the patient self-administered aspirin after symptom onset.

Target Value: N/A Selections: (1) No (2) Yes Supporting Definitions: (none)

Source: Adapted from AHA Get with the Guidelines ACTION registry

**Fibrinolysis Prior to Arrival**

Coding Instructions: Indicate whether the patient was given a fibrinolytic by a health provider

(EMS, transferring hospital, etc.) prior to arrival at this hospital. Target Value: N/A

Selections: (1) No (2) Yes. If yes, please specify the name of the fibrinolytic agent that was

used.

Supporting Definitions: (none)

Source: Adapted from AHA Get with the Guidelines ACTION registry

**Glucose Insulin Potassium (GIK) Solution Prior to Arrival**

Coding Instructions: Indicate whether the patient was given GIK by a health provider (EMS, transferring hospital, etc.) prior to arrival at this hospital

Target Value: N/A Selections: (1) No (2) Yes Supporting Definitions: (none) Source: Definition per CORE team

**CPR/Chest Compressions Prior to Arrival**

Coding Instructions: Indicate if cardiopulmonary resuscitation (CPR) in any form, including chest compression, was performed for the patient, prior to arrival

Target Value: Any occurrence between first medical contact and arrival to this facility

Selections: (1) No (2) Yes

Supporting Definitions: CPR comprises of a series of interventions performed for patients with sudden cardiac arrest in order to restore the perfusion and oxygenation of vital organs. Chest or abdominal compression, ascertainment of patent airways, rescue breathing, as well as electrical cardioversion and defibrillation are the cornerstones of CPR. Medications (including

epinephrine, lidocaine, amiodarone, and atropine) may or may not be used during CPR. Source: Definition per CORE team from: Field JM, Hazinski MF, Sayre MR, Chameides L, Schexnayder SM, Hemphill R, Samson RA, Kattwinkel J, Berg RA, Bhanji F, Cave DM, Jauch EC, Kudenchuk PJ, Neumar RW, Peberdy MA, Perlman JM, Sinz E, Travers AH, Berg MD, Billi JE, Eigel B, Hickey RW, Kleinman ME, Link MS, Morrison LJ, O'Connor RE, Shuster M, Callaway CW, Cucchiara B, Ferguson JD, Rea TD, Vanden Hoek TL. Part 1: executive summary: 2010 American Heart Association Guidelines for Cardiopulmonary Resuscitation and Emergency Cardiovascular Care. Circulation. 2010;122(suppl 3):S640–S656.

**External Defibrillation Prior to Arrival**

Coding Instructions: Indicate if there is documentation of electrical defibrillation by lay responders or EMS personnel prior to arrival

Target Value: N/A

Selections: (1) No (2) Yes Supporting Definitions: (none) Source: Definition per CORE team

**Temporary Cardiac Pacing Prior to Arrival**

Coding Instructions: Indicate if there is documentation of temporary cardiac transcutaneous or transvenous pacing by EMS personnel prior to arrival

Target Value: N/A Selections: (1) No (2) Yes Supporting Definitions: (none) Source: Definition per CORE team

**Cardiac Arrest Prior to Admission**

Coding Instructions: Indicate if the patient had an episode of cardiac arrest prior to admission to this facility.

Note(s): Evaluated by ED personnel and either (1) received attempts at external defibrillation or chest compressions or (2) were pulseless but did not receive attempts to defibrillate or cardiopulmonary resuscitation (CPR).

Target Value: Any occurrence prior to admission to this facility. Selections: (1) No (2) Yes

Supporting Definitions: 'Sudden' cardiac arrest is the sudden cessation of cardiac activity so that

the victim becomes unresponsive, with no normal breathing and no signs of circulation. If corrective measures are not taken rapidly, this condition progresses to sudden death. Cardiac arrest should be used to signify an event as described above that is reversed, usually by CPR, and/or defibrillation or cardioversion, or cardiac pacing. Sudden cardiac arrest is not the same as sudden cardiac death. Sudden cardiac death describes a fatal event.

Source: ACC/AHA/HRS 2006 Key Data Elements and Definitions for Electrophysiological

Studies and Procedures; adapted from AHA Get with the Guidelines ACTION registry

**Antiplatelet Therapy in the Emergency Department Prior to Admission**

Coding Instructions: Indicate whether the patient was given aspirin, clopidogrel, ticlopidine or other antiplatelet therapy by a health provider (EMS, transferring hospital, etc.) in the emergency department prior to admission.

Target Value: Any occurrence between arrival to the ED and admission

Selections: (1) No (2) Yes

Supporting Definitions: (none)

Source: Adapted from AHA Get with the Guidelines ACTION registry

**Fibrinolysis in the Emergency Department Prior to Admission**

Coding Instructions: Indicate whether the patient was given a fibrinolytic by a health provider

(EMS, transferring hospital, etc.) in the emergency department prior to admission. Target Value: Any occurrence between arrival to the ED and admission

Selections: (1) No (2) Yes. If yes, please specify the name of the fibrinolytic agent that was used.

Supporting Definitions: (none)

Source: Adapted from AHA Get with the Guidelines ACTION registry

**Glucose Insulin Potassium (GIK) Solution in the Emergency Department Prior to**

**Admission**

Coding Instructions: Indicate whether the patient was given GIK by a health provider (EMS, transferring hospital, etc.) in the emergency department prior to admission.

Target Value: Any occurrence between arrival to the ED and admission

Selections: (1) No (2) Yes Supporting Definitions: (none) Source: Definition per CORE team

**CPR/Chest Compressions in the Emergency Department Prior to Admission**

Coding Instructions: Indicate if cardiopulmonary resuscitation (CPR) in any form, including chest compression, was performed for the patient in the emergency department prior to admission Target Value: Any occurrence between arrival to the ED and admission

Selections: (1) No (2) Yes

Supporting Definitions: CPR comprises of a series of interventions performed for patients with sudden cardiac arrest in order to restore the perfusion and oxygenation of vital organs. Chest or abdominal compression, ascertainment of patent airways, rescue breathing, as well as electrical cardioversion and defibrillation are the cornerstones of CPR. Medications (including

epinephrine, lidocaine, amiodarone, and atropine) may or may not be used during CPR. Source: Definition per CORE team from: Field JM, Hazinski MF, Sayre MR, Chameides L, Schexnayder SM, Hemphill R, Samson RA, Kattwinkel J, Berg RA, Bhanji F, Cave DM, Jauch EC, Kudenchuk PJ, Neumar RW, Peberdy MA, Perlman JM, Sinz E, Travers AH, Berg MD, Billi JE, Eigel B, Hickey RW, Kleinman ME, Link MS, Morrison LJ, O'Connor RE, Shuster M, Callaway CW, Cucchiara B, Ferguson JD, Rea TD, Vanden Hoek TL. Part 1: executive summary: 2010 American Heart Association Guidelines for Cardiopulmonary Resuscitation and Emergency Cardiovascular Care. Circulation. 2010;122(suppl 3):S640–S656.

**External Defibrillation in Emergency Department Prior to Admission**

Coding Instructions: Indicate if there is documentation of electrical defibrillation in the emergency department prior to admission

Target Value: Any occurrence between arrival to the ED and admission

Selections: (1) No (2) Yes

Supporting Definitions: (none) Source: Definition per CORE team

**Temporary Cardiac Pacing In Emergency Department Prior to Admission**

Coding Instructions: Indicate if there is documentation of temporary transcutaneous or transvenous cardiac pacing in the emergency department prior to admission

Target Value: Any occurrence between arrival to the ED and admission

Selections: (1) No (2) Yes Supporting Definitions: (none) Source: Definition per CORE team

**ACUTE COEXISTING CONDITIONS AT PRESENTATION TO THIS FACILITY Heart Failure on Presentation to This Facility**

Coding Instructions: Indicate if there is physician documentation or report of HF on presentation to this facility

Target Value: Any occurrence between first medical contact and arrival at this facility

Selections: (1) No (2) Yes

Supporting Definitions: Heart failure is defined as physician documentation or report of any of the following clinical symptoms of heart failure, described as: unusual dyspnea on light exertion, recurrent dyspnea occurring in the supine position, fluid retention; or the description of rales, jugular venous distension, pulmonary edema on physical exam, or pulmonary edema on chest

x-ray presumed to be due to cardiac dysfunction. A low ejection fraction without clinical evidence of heart failure does not qualify as heart failure.

Source: Acute Coronary Syndromes Data Standards (JACC 2001 38: 2114 - 30), The Society of

Thoracic Surgeons; adapted from AHA Get with the Guidelines ACTION registry

**Cardiogenic Shock on Presentation to This Facility**

Coding Instructions: Indicate if the patient was in a state of cardiogenic shock on presentation to this facility.

Note(s): Transient episodes of hypotension reversed with IV fluid or atropine do not constitute cardiogenic shock. The hemodynamic compromise (with or without extraordinary supportive therapy) must persist for at least 30 minutes.

Target Value: Any occurrence between first medical contact and arrival at this facility

Selections: (1) No (2) Yes

Supporting Definitions: Cardiogenic shock is defined as a sustained (>30 minutes) episode of systolic blood pressure <90 mm Hg, and/or cardiac index <2.2 L/min/m2 determined to be secondary to cardiac dysfunction, and/or the requirement for parenteral inotropic or vasopressor agents or mechanical support (e.g., IABP, extracorporeal circulation, ventricular assist devices) to maintain blood pressure and cardiac index above those specified levels.

Source: Acute Coronary Syndromes Data Standards (JACC 2001 38: 2114 - 30); adapted from

AHA Get with the Guidelines ACTION registry

**Pneumonia on Presentation to This Facility**

Coding Instructions: Indicate if there is physician documentation or report of pneumonia on presentation to this facility

Target Value: Any occurrence between first medical contact and arrival at this facility

Selections: (1) No (2) Yes

Supporting Definitions: Pneumonia is defined as physician documentation of pneumonia plus the presence of a demonstrable infiltrate by chest radiograph or other imaging.

Source: Infectious Diseases Society of America/American Thoracic Society consensus guidelines on the management of community-acquired pneumonia in adults. Clin Infect Dis.

2007 Mar 1;44 Suppl 2:S27-72; definition per CORE team

**COPD Exacerbation on Presentation to This Facility**

Coding Instructions: Indicate if there is physician documentation or report of exacerbated

(acute) COPD on presentation to this facility

Target Value: Any occurrence between first medical contact and arrival at this facility

Selections: (1) No (2) Yes

Supporting Definitions: Exacerbated COPD is defined as physician documentation of exacerbated COPD. Other clinical signs and radiographic findings are non-specific. Source: Definition per CORE team

**Acute Stroke on Presentation to This Facility**

Coding Instructions: Indicate if there is physician documentation or report of an acute stroke on presentation to this facility.

Target Value: Any occurrence between first medical contact and arrival at this facility

Selections: (1) No (2) Yes

Supporting Definitions: Acute stroke is defined as physician documentation of an acute stroke

Source: Definition per CORE team

**Active Gastrointestinal Bleeding on Presentation to This Facility**

Coding Instructions: Indicate if there is physician documentation or report of active gastrointestinal bleeding on presentation to this facility.

Target Value: Any occurrence between first medical contact and arrival at this facility

Selections: (1) No (2) Yes

Supporting Definitions: Active gastrointestinal bleeding is defined as physician documentation of active hematemesis (upper gastrointestinal bleeding), active hematochezia (lower gastrointestinal bleeding), or active melena (upper gastrointestinal bleeding).

Source: Definition per CORE team

**Acute Renal Failure on Presentation to This Facility**

Coding Instructions: Indicate if there is physician documentation or report of acute renal failure on presentation to this facility

Target Value: Any occurrence between first medical contact and arrival at this facility

Selections: (1) No (2) Yes

Supporting Definitions: Acute renal failure is defined as physician documentation of acute renal failure

Source: Definition per CORE team

**Acute Trauma on Presentation to This Facility**

Coding Instructions: Indicate if there is physician documentation or report of acute trauma on presentation to this facility

Target Value: Any occurrence between first medical contact and arrival at this facility

Selections: (1) No (2) Yes

Supporting Definitions: Acute trauma is defined as physician documentation of acute trauma at presentation to the facility.

Source: Definition per CORE team

**NYHA Functional Classification on Arrival**

Coding Instructions: Indicate the physician documentation of New York Heart Association functional class on the patient’s arrival to this facility

Target Value: N/A

Selections:

I – No limitation of physical activity. Ordinary physical activity does not cause undue fatigue, palpitation, or dyspnea (shortness of breath)

II - Slight limitation of physical activity. Comfortable at rest, but ordinary physical activity

results in fatigue, palpitation, or dyspnea.

III - Marked limitation of physical activity. Comfortable at rest, but less than ordinary activity causes fatigue, palpitation, or dyspnea.

IV - Unable to carry out any physical activity without discomfort. Symptoms of cardiac insufficiency present at rest. If any physical activity is undertaken, discomfort is increased. Unrecorded

Supporting Definitions: The NYHA functional classification system relates symptoms to everyday activities and the patient's quality of life.

Source: Definition per CORE team; The Criteria Committee of the New York Heart Association. Diseases of the heart and blood vessels. In: Nomenclature and Criteria for Diagnosis. 7th ed. Boston: Little, Brown, 1973:286.

**Killip Classification on Arrival**

Coding Instructions: Indicate the physician documentation of Killip classification on the patient’s arrival to this facility

Target Value: N/A

Selections:

I - No rales over the lung fields and no S3.

II - Rales 50% or less over the lung fields or presence of an S3. Includes patients documented as having bibasilar rales.

III - Rales more than 50% of the lung fields/frank pulmonary edema. Includes patients documented as having rales throughout.

IV - Cardiogenic Shock

Unrecorded

Supporting Definitions: Killip classification is a method of prognostication and risk-stratification in patients with acute myocardial infarction.

Source: Adapted from VIRGO registry

**PAST MEDICAL HISTORY – RELATED TO HEART DISEASE History of Angina or Coronary Heart Disease**

Coding Instructions: Mark “Yes”, if documented history of angina or coronary heart disease is present

Target Value: Any occurrence between birth and arrival at this facility

Selections: (1) No (2) Yes

Supporting Definitions: Indicate if there is physician documentation of history of angina or history of coronary artery disease. Coronary heart disease may be abbreviated as CHD. Presence of at least one of the two warrants a “Yes” answer.

Source: Definition per CORE team

**History of Myocardial Infarction**

Coding Instructions: Indicate if the patient has had at least one documented previous myocardial infarction.

Target Value: Any occurrence between birth and arrival at this facility

Selections: (1) No (2) Yes

Supporting Definitions: A myocardial infarction is evidenced by any of the following:

1. A rise and fall of cardiac biomarkers (preferably troponin) with at least one of the values in the abnormal range for that laboratory [typically above the 99th percentile of the upper reference limit (URL) for normal subjects] together with at least one of the following manifestations of myocardial ischemia:

a. Ischemic symptoms.

b. ECG changes indicative of new ischemia (new ST-T changes, new left bundle branch block, or loss of R wave voltage).

c. Development of pathological Q- waves in 2 or more contiguous leads in the

ECG (or equivalent findings for true posterior MI).

d. Imaging evidence of new loss of viable myocardium or new regional wall motion abnormality.

e. Documentation in the medical record of the diagnosis of acute myocardial infarction based on the cardiac biomarker pattern in the absence of any items enumerated in a-d due to conditions that may mask their appearance (e.g., peri- operative infarct when the patient cannot report ischemic symptoms; baseline left bundle branch block or ventricular pacing).

2. ECG changes associated with prior myocardial infarction can include the following

(with or without prior symptoms):

a. Any Q-wave in leads V2-V3 >=0.02 seconds or QS complex in leads V2 and

V3.

b. Q-wave >=0.03 seconds and >=0.1 mV deep or QS complex in leads I, II, aVL, aVF, or V4-V6 in any two leads of a contiguous lead grouping (I, aVL, V6; V4-V6; II, III, and aVF).

c. R-wave >=0.04 seconds in V1-V2 and R/S >=1 with a concordant positive T-

wave in the absence of a conduction defect.

3. Imaging evidence of a region with new loss of viable myocardium at rest in the absence of a non-ischemic cause. This can be manifest as:

a. Echocardiographic, CT, MR, ventriculographic or nuclear imaging evidence of left ventricular thinning or scarring and failure to contract appropriately (i.e., hypokinesis, akinesis, or dyskinesis).

b. Fixed (non-reversible) perfusion defects on nuclear radioisotope imaging (e.g.,

MIBI, thallium).

4. Medical record documentation of prior myocardial infarction.

Source: Joint ESC-ACC-AHA-WHF 2007 Task Force Consensus Document "Universal

Definition of Myocardial Infarction"; adapted from AHA Get with the Guidelines ACTION registry

**History of Heart Failure**

Coding Instructions: Indicate if there is a previous history of heart failure.

Note(s): A previous hospital admission with principal diagnosis of heart failure is considered evidence of heart failure history.

Target Value: Any occurrence between birth and arrival at this facility

Selection: (1) No (2) Yes

Supporting Definitions: Heart failure is defined as physician documentation or report of any of the following clinical symptoms of heart failure described as unusual dyspnea on light exertion, recurrent dyspnea occurring in the supine position, fluid retention; or the description of rales, jugular venous distension, pulmonary edema on physical exam, or pulmonary edema on chest x-ray presumed to be cardiac dysfunction. A low ejection fraction without clinical evidence of heart failure does not qualify as heart failure.

Source: Acute Coronary Syndromes Data Standards (JACC 2001 38: 2114 - 30), The Society of

Thoracic Surgeons; adapted from AHA Get with the Guidelines ACTION registry

**History of Percutaneous Coronary Intervention**

Coding Instructions: Indicate if the patient had a previous percutaneous coronary intervention

(PCI) of any type (balloon angioplasty, stent or other).

Note (s): Timeframe does NOT include the current admission.

Target Value: Any occurrence between birth and arrival at this facility

Selections: (1) No (2) Yes

Supporting Definitions: A percutaneous coronary intervention (PCI) is the placement of an angioplasty guide wire, balloon, or other device (e.g. stent, atherectomy, brachytherapy, or thrombectomy catheter) into a native coronary artery or coronary artery bypass graft for the purpose of mechanical coronary revascularization.

Source: Adapted from AHA Get with the Guidelines ACTION registry

**History of Fibrinolysis**

Coding Instructions: Indicate if the patient has previously received intravenous fibrinolysis. Note(s): Timeframe does NOT include the current admission.

Target Value: Any occurrence between birth and arrival at this facility

Selections: (1) No (2) Yes

Supporting Definitions: (none)

Source: Adapted from AHA Get with the Guidelines ACTION registry

**History of CABG**

Coding Instructions: Indicate whether the patient had a coronary artery bypass graft (CABG). Note(s): Timeframe does NOT include the current admission.

Target Value: Any occurrence between birth and arrival at this facility

Selections: (1) No (2) Yes

Supporting Definitions: (none)

Source: Adapted from AHA Get with the Guidelines ACTION registry

**History of Atrial Fibrillation or Flutter**

Coding Instructions: Indicate if there is a previous history of atrial fibrillation or flutter

Note(s): Code "No" if patient was first diagnosed with atrial fibrillation or flutter after reperfusion during this admission. If there is no prior documentation of atrial arrhythmias, it is acceptable to code "No"

Target Value: Any occurrence between birth and arrival at this facility

Selections: (1) No (2) Yes Supporting Definitions: (none) Source: Definition per CORE team

**History of Ventricular Tachycardia or Ventricular Fibrillation**

Coding Instructions: Indicate if there is a previous history of ventricular tachycardia or ventricular fibrillation

Note(s): Code "No" if patient was first diagnosed with ventricular tachycardia or fibrillation after reperfusion during this admission. If there is no prior documentation of ventricular arrhythmias, it is acceptable to code "No"

Target Value: Any occurrence between birth and arrival at this facility

Selections: (1) No (2) Yes Supporting Definitions: (none) Source: Definition per CORE team

**History of Permanent Pacemaker**

Coding Instructions: Indicate if the patient has a history of having a permanent pacemaker

Target Value: N/A Selections: (1) No (2) Yes

Supporting Definitions: Permanent pacemaker includes single-chamber, dual chamber, and biventricular pacemakers

Source: Definition per CORE team

**History of Automatic Implantable Cardioverter Defibrillator**

Coding Instructions: Indicate if the patient has a history of having an automatic implantable cardioverter defibrillator

Target Value: N/A

Selections: (1) No (2) Yes Supporting Definitions: (none) Source: Definition per CORE team

**GENERAL PAST MEDICAL HISTORY History of Hypertension**

Coding Instructions: Indicate if the patient has been diagnosed previously with hypertension Note(s): Code "No" if hypertension was first diagnosed after reperfusion during this admission. Selections: (1) No (2) Yes

Target Value: Any occurrence between birth and arrival at this facility

Supporting Definitions: Hypertension is defined by any one of the following:

1. History of hypertension diagnosed and treated with medication, diet and/or exercise

2. Prior documentation of blood pressure greater than 140 mm Hg systolic and/or 90 mm Hg diastolic for patients without diabetes or chronic kidney disease, or prior documentation of blood pressure greater than 130 mm Hg systolic or 80 mm Hg diastolic on at least two occasions for patients with diabetes or chronic kidney disease.

3. Currently on pharmacological therapy for the treatment of hypertension.

Source: Acute Coronary Syndromes Data Standards (JACC 2001 38: 2114 - 30), The Society of

Thoracic Surgeons; adapted from AHA Get with the Guidelines ACTION registry

**History of Dyslipidemia**

Coding Instructions: Indicate if the patient has a history of dyslipidemia diagnosed and/or treated by a physician.

Target Value: Any occurrence between birth and arrival at this facility

Selections: (1) No (2) Yes

Supporting Definitions: Dyslipidemia is defined by the National Cholesterol Education Program criteria and includes documentation of the following:

1. Total cholesterol greater than 200 mg/dL (5.18 mmol/l); or

2. Low-density lipoprotein (LDL) greater than or equal to 130 mg/dL (3.37 mmol/l); or

3. High-density lipoprotein (HDL) less than 40 mg/dL (1.04 mmol/l).

4. Currently on pharmacologic therapy for the treatment of dyslipidemia.

For patients with known coronary artery disease, treatment is initiated if LDL is greater than 100 mg/dL (2.59 mmol/l), and this would qualify as hypercholesterolemia.

Source: Acute Coronary Syndromes Data Standards (JACC 2001 38: 2114 - 30), The Society of

Thoracic Surgeons; adapted from AHA Get with the Guidelines ACTION registry

**History of Chronic Renal Failure**

Coding Instructions: Indicate if the patient has a history of chronic renal failure Target Value: Any occurrence between birth and arrival at this facility Selections: (1) No (2) Yes

Supporting Definitions: Chronic renal failure can be coded for any of the following:

1. A documented history of renal failure, and/or

2. A history of creatinine > 2.0 mg/dL, and/or

3. A documented history of chronic renal disease

Prior renal transplant patients are not included unless creatinine has been >2.0 mg/dL

since transplantation

Source: Adapted from VIRGO registry

**Currently on Dialysis**

Coding Instructions: Indicate if the patient is currently undergoing either hemodialysis or peritoneal dialysis on an ongoing basis as a result of renal failure.

Note(s): Code "No" if patient was not on dialysis until after reperfusion during this admission. Target Value: The value on arrival at this facility

Selections: (1) No (2) Yes

Supporting Definitions: (none)

Source: Adapted from AHA Get with the Guidelines ACTION registry

**History of Chronic Lung Disease**

Coding Instructions: Indicate if the patient has a history of chronic lung disease.

A history of chronic inhalation reactive disease (asbestosis, mesothelioma, black lung disease or pneumoconiosis) qualifies as chronic lung disease. Radiation induced pneumonitis or radiation fibrosis also qualifies as chronic lung disease. A history of atelectasis is a transient condition and does not qualify.

Notes: Code "No" if patient was first diagnosed with chronic lung disease after reperfusion during this admission.

Target Value: Any occurrence between birth and arrival at this facility

Selections: (1) No (2) Yes

Supporting Definitions: Chronic lung disease can include patients with chronic obstructive pulmonary disease, chronic bronchitis, or emphysema. It can also include a patient who is currently being chronically treated with inhaled or oral pharmacological therapy (e.g., beta- adrenergic agonist, anti-inflammatory agent, leukotriene receptor antagonist, or steroid). Patients with asthma or seasonal allergies are not considered to have chronic lung disease. Source: Adapted from AHA Get with the Guidelines ACTION registry

**History of Asthma**

Coding Instructions: Mark “Yes”, if there is physician documentation of a history of asthma

Target Value: N/A Selections: (1) No (2) Yes Supporting Definitions: (none) Source: Definition per CORE team

**History of Diabetes Mellitus. History and Risk Factors**

Coding Instructions: Indicate if the patient has a history of diabetes mellitus, regardless of duration of disease or need for antidiabetic agents.

Note(s): Code "No" if the patient was first diagnosed with diabetes mellitus after reperfusion during this admission.

Target Value: Any occurrence between birth and arrival at this facility

Selections: (1) No (2) Yes

Supporting Definitions: Diabetes mellitus is diagnosed by a physician or can be defined as a fasting blood sugar greater than 7 mmol/l or 126 mg/dL. It does not include gestational diabetes. Diabetes mellitus can also be identified by history of pharmacologic treatment for condition. Source: Acute Coronary Syndromes Data Standards (JACC 2001 38: 2114 - 30), The Society of Thoracic Surgeons; adapted from AHA Get with the Guidelines ACTION registry

**History of Peripheral Vascular Disease**

Coding Instructions: Indicate if there is physician documentation of a history of peripheral vascular disease

Target Value: N/A Selections: (1) No (2) Yes

Supporting Definitions: Mark “Yes” if there is exact mention of “a history of peripheral vascular

disease,” or extremity claudication, or history of lower extremity percutaneous or surgical revascularization procedure.

Source: Definition per CORE team

**History of Venous Thromboembolic Disease**

Coding Instructions: Mark “yes” if documented history of venous thromboembolic disease. Target Value: N/A

Selections: (1) No (2) Yes

Supporting Definitions: Venous thromboembolic disease is also called VTE. VTE includes both pulmonary embolism (PE) and deep vein thrombosis (DVT).

Source: Adapted from VIRGO registry

**Cancer Other than Skin Cancer**

Coding Instructions: Mark “Yes”, if documented history of malignancy (hematological, solid organ, or metastases), excluding skin cancer

Target Value: N/A Selections: (1) No (2) Yes

Supporting Definitions: Cancer includes carcinoma, sarcoma, melanoma, leukemia (any type), lymphoma, Hodgkin’s disease, myeloma, or malignant tumor.

Source: Modified from VIRGO registry

**Cancer Treatment within the Past 6 Months**

Coding Instructions: Indicate if there is documentation about curative or palliative cancer therapy within 6 months prior to the index admission

Target Value: N/A Selections: (1) No (2) Yes

Supporting Definitions: Mark “Yes” if the patient received any surgery, or any chemotherapy, or

any radiotherapy, or any palliative or hospice services because of a cancer diagnosis, or any combination of these, related to a diagnosed cancer.

Source: Definition per CORE team

**Major Surgery Within the Past Four Weeks**

Coding Instructions: Indicate if the patient has a history of major surgery within the past four weeks prior to the index admission

Target Value: Any occurrence within four weeks prior to index admission. Selections: (1) No (2) Yes

Supporting Definitions: Major surgery is defined as any surgery that requires general

anesthesia, or that involves opening the great cavities of body (cranium, chest, abdomen, pelvic cavity), or those in which severe bleeding is likely to occur, or that is likely to be life-threatening. Examples include:

1. Cranial surgery (including decompression following a bleed, tumor removal, or others)

2. Thoracic surgery (including coronary bypass surgery, heart valve surgery, other heart

surgery, any lung surgery, any mediastinal surgery)

3. Abdominal surgery (any surgery related to organs in the abdominal cavity, including the

stomach, bowels, liver, kidneys, or others)

4. Pelvic surgery (including Cesarean section surgery, vaginal hysterectomy, cystocele

surgery, rectocele surgery, or others)

5. Any surgery not meeting the above criteria but being performed under general

anesthesia

6. Any surgery not meeting the above criteria but lasting more than 60 minutes (e.g. orthopedic surgery lasting for >60 minutes, other types of surgery lasting for >60 minutes).

The definition is meant to be distinct from minor surgery. Minor surgery would be defined as any invasive procedure in which only the skin or mucus membranes and connective tissues are resected. Examples include:

1. Vascular cutdown for catheter placement

2. Implanting pumps in subcutaneous tissue

3. Biopsy procedures

4. Suturing of superficial skin or mucosal lacerations

Source: Definition per CORE team, based on: Juul AB, Wetterslev J, Kofoed-Enevoldsen A, Callesen T, Jensen G, Gluud C; Diabetic Postoperative Mortality and Morbidity group. The Diabetic Postoperative Mortality and Morbidity (DIPOM) trial: rationale and design of a multicenter, randomized, placebo-controlled, clinical trial of metoprolol for patients with diabetes mellitus who are undergoing major noncardiac surgery. Am Heart J. 2004; 147: 677-83. and Holford CP. Graded compression for preventing deep venous thrombosis. Br Med J. 1976; 2:

969-70. and Kucher N, Koo S, Quiroz R, Cooper JM, Paterno MD, Soukonnikov B, Goldhaber SZ. Electronic alerts to prevent venous thromboembolism among hospitalized patients. N Engl J Med. 2005; 352: 969-77. and Earl R. Definition of major and minor surgery: A question and an answer. Ann Surg. 1917; 65: 799. and Wicki J, Perneger TV, Junod AF, Bounameaux H, Perrier A. Assessing clinical probability of pulmonary embolism in the emergency ward: a simple score. Arch Intern Med. 2001; 161: 92-7.

**History of Hepatitis B Infection**

Coding Instructions: Mark “Yes”, if documented history of hepatitis B infection

Target Value: N/A Selections: (1) No (2) Yes Supporting Definitions: (none) Source: Definition per CORE team

**History of Hepatitis C Infection**

Coding Instructions: Mark “Yes”, if documented history of hepatitis C infection

Target Value: N/A

Selections: (1) No (2) Yes Supporting Definitions: (none) Source: Definition per CORE team

**History of Liver Cirrhosis**

Coding Instructions: Mark “Yes”, if documented history of liver cirrhosis is present

Target Value: N/A Selections: (1) No (2) Yes Supporting Definitions: (none) Source: Definition per CORE team

**Prior Carotid Artery Surgery/Intervention**

Coding Instructions: Indicate if the patient has a history of prior carotid artery surgery or stenting

Target Value: Any occurrence between birth and arrival at this facility

Selections: (1) No (2) Yes

Supporting Definitions: Previous carotid artery surgery/ intervention for carotid artery stenosis. This does not include neurological disease processes such as metabolic and/or anoxic ischemic encephalopathy.

Source: The Society of Thoracic Surgeons; adapted from AHA Get with the Guidelines ACTION

registry

**Prior Ischemic Stroke**

Coding Instructions: Indicate if the patient has had an ischemic stroke. Target Value: Any occurrence between birth and arrival at this facility Selections: (1) No (2) Yes

Supporting Definitions: A prior stroke is defined as any confirmed neurological deficit of abrupt onset caused by a disturbance in cerebral blood supply that did not resolve within 24 hours. Source: Adapted from AHA Get with the Guidelines ACTION registry

**Prior Hemorrhagic Stroke**

Coding Instructions: Indicate if the patient has had a hemorrhagic stroke. Target Value: Any occurrence between birth and arrival at this facility Selections: (1) No (2) Yes

Supporting Definitions: A prior stroke is defined as any confirmed neurological deficit of abrupt

onset caused by a disturbance in cerebral blood supply that did not resolve within 24 hours. Source: Adapted from AHA Get with the Guidelines ACTION registry

**Prior Stroke, Unknown Subtype**

Coding Instructions: Indicate if the patient has had a stroke, but subtype (ischemic, hemorrhagic) is unknown.

Target Value: Any occurrence between birth and arrival at this facility

Selections: (1) No (2) Yes

Supporting Definitions: A prior stroke is defined as any confirmed neurological deficit of abrupt onset caused by a disturbance in cerebral blood supply that did not resolve within 24 hours. Source: Adapted from AHA Get with the Guidelines ACTION registry

**PERSONAL HISTORY Smoking History**

Coding Instructions: Indicate the smoking status of the patient

Target Value: N/A

Selections: (1) Never smoked (2) Current Smoker (3) Past Smoker (4) Unrecorded

Supporting Definitions: Past smoker is defined as a person who was a daily smoker in the past and stopped smoking at least three months prior to admission date, or chart documentation of “past smoker”

Source: Definition per CORE team, based on: McCabe RE, Chudzik SM, Antony MM, Young L, Swinson RP, Zolvensky MJ. Smoking behaviors across anxiety disorders. J Anxiety Disord

2004; 18: 7-18.

**Smoking Duration among Current Smokers**

Coding Instructions: Indicate the duration of time since the patient first started smoking

Target Value: N/A Selections: (none)

Supporting Definitions: Provide the duration in months

Source: Definition per CORE team

**Smoking Duration for Past Smokers**

Coding Instructions: Indicate the duration of time between when the patient first started smoking and the cessation of smoking

Target Value: N/A

Selections: (none)

Supporting Definitions: Provide the duration in months

Source: Definition per CORE team

**Smoking Frequency**

Coding Instructions: Indicate the average number of cigarettes smoked per day

Target Value: N/A Selections: (none)

Supporting Definitions: Provide the information as cigarettes per day

Source: Definition per CORE team

**Pregnancy Status**

Coding Instructions: Indicate if the patient is currently pregnant. Target Value: N/A

Selections: (1) No (2) Yes

Supporting Definitions: (none)

Source: Adapted from VIRGO registry

**Postpartum Status**

Coding Instructions: Indicate if the patient is currently postpartum

Target Value: N/A Selections: (1) No (2) Yes

Supporting Definitions: The postpartum period begins immediately after birth and extends for about 6 weeks.

Source: Adapted from VIRGO registry

**Menopausal Status**

Coding Instructions: Indicate if the patient is currently postmenopausal

Target Value: N/A Selections: (1) No (2) Yes

Supporting Definitions: Menopause implies at least 12 months of amenorrhea after the final menstrual period

Source: Definition by CORE team

**PHYSICAL EXAMINATION**

**Heart Rate at Presentation to This Facility**

Coding Instructions: Indicate the first measurement or earliest record of heart rate (in beats per minute).

Note(s): Measurement from EMS or the transferring facility is also acceptable. Target Value: The first value after arrival at this facility

Selections: (none)

Supporting Definitions: (none)

Source: Adapted from AHA Get with the Guidelines ACTION registry

**Systolic Blood Pressure at Presentation to This Facility**

Coding Instructions: Indicate the first measurement or earliest record of systolic blood pressure

(in mmHg).

Note(s): Measurement from EMS or the transferring facility is also acceptable. Target Value: The first value after arrival at this facility

Selections: (none)

Supporting Definitions: (none)

Source: Adapted from AHA Get with the Guidelines ACTION registry

**Diastolic Blood Pressure at Presentation to This Facility**

Coding Instructions: Indicate the first measurement or earliest record of diastolic blood pressure

(in mmHg).

Note(s): Measurement from EMS or the transferring facility is also acceptable. Target Value: The first value after arrival at this facility

Selections: (none)

Supporting Definitions: (none)

Source: Adapted from AHA Get with the Guidelines ACTION registry

**Temperature at Presentation to This Facility**

Coding Instructions: Indicate the first measurement or earliest record of temperature (degrees

Celsius).

Note(s): Measurement from EMS or the transferring facility is also acceptable. Target Value: The first value after arrival at this facility

Selections: (none)

Supporting Definitions: (none)

Source: Adapted from AHA Get with the Guidelines ACTION registry

**Respiratory Rate at Presentation to This Facility**

Coding Instructions: Indicate the first measurement or earliest record of respiratory rate (breaths per minute).

Note(s): Measurement from EMS or the transferring facility is also acceptable.

Target Value: The first value after arrival at this facility

Selections: (none)

Supporting Definitions: (none)

Source: Adapted from AHA Get with the Guidelines ACTION registry

**Specify Home Medications**

Coding Instructions: Indicate the names of medications been taken by patient routinely at home prior to this hospitalization

Note(s): "Routinely" refers to the daily use of medications as prescribed, even if the patient misses a dose

Target Value: Any occurrence between 2 weeks prior to first medical contact and first medical

contact

Selections: (none)

Supporting Definitions: (none)

Source: Adapted from AHA Get with the Guidelines ACTION registry

**AUXILIARY EXAMINATION Was ECG Obtained at Hospital**

Coding Instructions: Indicate if an ECG was obtained at the hospital

Target Value: N/A Selections: (1) No (2) Yes

Supporting Definitions: This will be considered for the first 5 ECGs in the hospital

Source: Definition per CORE team

**Date of ECG at Hospital**

Coding Instructions: Indicate the date ECG was obtained at the hospital

Target Value: N/A Selections: (none)

Supporting Definitions: This will be considered for the first 5 ECGs in the hospital

Source: Definition per CORE team

**Time of ECG at Hospital**

Coding Instructions: Indicate the time ECG was obtained at the hospital

Target Value: N/A Selections: (none)

Supporting Definitions: This will be considered for the first 5 ECGs in the hospital

Source: Definition per CORE team

**ECG Results**

Coding Instructions: Indicate the documented physician interpretation of the ECG Note(s): Mark as many of the choices that apply.

Target Value: N/A

Selections: (1) acute myocardial infarction (2) left bundle branch block (3) ST-elevation myocardial infarction (4) ST-depression (5) Q-wave myocardial infarction (6) ventricular fibrillation (7) ventricular tachycardia (8) atrial fibrillation (9) 2nd degree atrioventricular block type 1 (10) 2nd degree atrioventricular block type 2 (11) 3rd degree atrioventricular block Source: Definition per China team

**OTHER DIAGNOSTIC TESTS Chest X-ray**

Coding Instructions: Indicate whether the patient underwent a chest X-ray or not

Target Value: Any occurrence between arrival at admitting hospital and discharge

Selections: (1) No (2) Yes

Supporting Definition: (none)

Source: Adapted from AHA Get with the Guidelines ACTION registry

**Chest X-ray Date**

Coding Instructions: Indicate the date of chest X-ray.

Target Value: If more than one chest X-ray was obtained, then indicate the date of each chest

X-ray.

Selections: (none) Supporting Definition: (none)

Source: Adapted from AHA Get with the Guidelines ACTION registry

**Pulmonary Edema on Chest X-ray**

Coding Instructions: Indicate whether pulmonary edema was diagnosed on each chest X-ray or not

Target Value: Any occurrence between arrival at admitting hospital and discharge

Selections: (1) No (2) Yes

Supporting Definition: (none)

Source: Adapted from AHA Get with the Guidelines ACTION registry

**Pulmonary Infiltrate on Chest X-ray**

Coding Instructions: Indicate whether a pulmonary infiltrate consistent with pneumonia was diagnosed on each chest X-ray or not

Target Value: Any occurrence between arrival at admitting hospital and discharge

Selections: (1) No (2) Yes Supporting Definition: (none) Source: Definition per CORE team

**Echocardiography**

Coding Instructions: Indicate whether the patient underwent echocardiography or not Target Value: Any occurrence between arrival at admitting hospital and discharge Selections: (1) No (2) Yes

Supporting Definition: (none)

Source: Adapted from AHA Get with the Guidelines ACTION registry

**Echocardiography Date**

Coding Instructions: Indicate the date of echocardiography.

Target Value: If more than one echocardiogram was obtained, then indicate the date of echocardiography closest to discharge.

Selections: N/A

Supporting Definition: (none)

Source: Adapted from VIRGO registry

**Echocardiography Ejection Fraction (EF) Value**

Instructions: Indicate the value of EF in percent documented in the echocardiography report. Target Value: Any occurrence between arrival at admitting hospital and discharge

Selections: (none)

Supporting Definition: LVEF: The left ventricular ejection fraction is the percentage of the blood emptied from the left ventricle at the end of the contraction. The left ventricular ejection fraction can be assessed via invasive (i.e. LV gram) or noninvasive (i.e. Echo, MR, CT or Nuclear)

testing.

Source: ACC Clinical Data Standards, The Society of Thoracic Surgeons; adapted from AHA Get with the Guidelines ACTION registry

**Stress Testing**

Coding Instructions: Indicate whether the patient underwent stress testing or not Target Value: Any occurrence between arrival at admitting hospital and discharge Selections: (1) No (2) Yes

Supporting Definition: (none)

Source: Adapted from AHA Get with the Guidelines registry

**Stress Testing Date**

Coding Instructions: Indicate date of stress testing.

Target Value: If more than one stress test was obtained, then indicate the date of stress test closest to discharge.

Selections: N/A

Supporting Definition: (none)

Source: Adapted from AHA Get with the Guidelines ACTION registry

**Type of Stress Test**

Coding Instructions: Indicate the type of stress testing performed

Target Value: N/A Selections:

1. ECG only

2. Radionuclide

3. Echocardiography

4. Cardiac MRI

5. Unrecorded

Supporting Definition: (none)

Source: Adapted from AHA Get with the Guidelines ACTION registry

**Method of Stress Test**

Coding Instructions: Indicate method of performing stress test

Target Value: N/A

Selections: (1) Exercise (2) Pharmacologic (3) Unknown

Supporting Definition: (none)

Source: Adapted from VIRGO registry

**Cardiac CT Angiogram**

Coding Instructions: Indicate if the patient received a cardiac CT scan (Cardiac Cat Scan) Target Value: Any occurrence between arrival at admitting hospital and discharge Selections: (1) No (2) Yes

Supporting Definition: (none)

Source: Adapted from VIRGO registry

**Cardiac CT Date**

Coding Instructions: Indicate date of cardiac CT. Target Value: N/A

Selections: N/A

Supporting Definition: (none)

Source: Adapted from VIRGO registry

**Cardiac CT Ejection Fraction (EF) Value**

Coding Instructions: Indicate the value of EF in percent documented in the CT scan report. Target Value: Any occurrence between arrival at admitting hospital and discharge Selections: (none)

Supporting Definition: The left ventricular ejection fraction is the percentage of the blood emptied from the left ventricle at the end of the contraction. The left ventricular ejection fraction can be accessed via invasive (i.e. LV gram) or noninvasive (i.e. Echo, MR, CT or Nuclear) testing.

Source: ACC Clinical Data Standards, the Society of Thoracic Surgeons; adapted from VIRGO

registry

**Multi-Gated Acquisition (MUGA) Scan**

Coding Instructions: Indicate whether the patient underwent MUGA or not

Target Value: Any occurrence between arrival at admitting hospital and discharge

Selections: (1) No (2) Yes Supporting Definition: (none) Source: Definition per CORE team

**MUGA Scan Ejection Fraction (EF) Value**

Coding Instructions: Indicate the value of EF in percent documented in the MUGA scan report. Target Value: Any occurrence between arrival at admitting hospital and discharge

Selections: (none)

Supporting Definition: LVEF: The left ventricular ejection fraction is the percentage of the blood emptied from the left ventricle at the end of the contraction. The left ventricular ejection fraction can be accessed via invasive (i.e. LV gram) or noninvasive (i.e. Echo, MR, CT or Nuclear) testing.

Source: ACC Clinical Data Standards, the Society of Thoracic Surgeons; definition per CORE

team

**LABORATORY TESTS**

**Initial Myohemoglobin Date and Time**

Coding Instructions: Indicate the date and time when the initial myohemoglobin sample was collected (not the date results reported).

Target Value: The first value between first medical contact and discharge

Selections: (none)

Supporting Definition: (none) Source: Definition per CORE team

**Units of Myohemoglobin**

Coding Instructions: Indicate the sample unit of measure of myohemoglobin level. Target Value: N/A

Selections:

(1) IU/L (2) ng/ml (3) mg/mL (4) Other

Supporting Definition: (none)

Source: Adapted from AHA Get with the Guidelines ACTION registry

**Initial Myohemoglobin Value**

Coding Instructions: Indicate myohemoglobin value.

Target Value: The first value between first medical contact and discharge

Selections: (none)

Supporting Definition: (none) Source: Definition per CORE team

**Maximum Myohemoglobin Date and Time**

Coding Instructions: Indicate the date and time of collection of the maximum myohemoglobin value.

Target Value: Any occurrence during the entire hospital stay

Selections: N/A

Supporting Definition: (none) Source: Definition per CORE team

**Maximum Myohemoglobin Value**

Coding Instructions: Indicate the value of maximum myohemoglobin. Target Value: Any occurrence during the entire hospital stay Selections: (none)

Supporting Definition: (none) Source: Definition per CORE team

**Initial Creatine Kinase (CK) Value**

Coding Instructions: Indicate the value of the initial CK.

Notes: Initial CK level corresponds to the first sample obtained within the first 24 hours of care. It may also have been collected at the transferring hospital.

Target Value: Any occurrence between first medical contact and 24 hours after arrival at first facility

Selections: N/A

Supporting Definition: (none)

Source: Adapted from AHA Get with the Guidelines ACTION registry

**Unit of Creatine Kinase (CK) Level**

Coding Instructions: Indicate the sample unit of measure of CK level. Target Value: N/A

Selections:

(1) IU/L (2) %

(3) mg/mL

(4) ng/mL (5) mg/IU (6) MI/IU

Supporting Definition: (none)

Source: Adapted from AHA Get with the Guidelines ACTION registry

**Initial CK Upper Limit of Normal (ULN)**

Coding Instructions: Indicate the ULN of the initial CK sample.

Note(s): If a range is given for ULN values, record the highest number in the range. Examples: If the reference range given is 0.0-1.5, record ULN as 1.5. If the reference range given is < 1.5, record ULN as 1.5 as well.

The initial sample value refers to the first sample obtained within the first 24 hours of care. Target Value: N/A

Selections: N/A

Supporting Definition: (none)

Source: Adapted from AHA Get with the Guidelines ACTION registry

**Initial Creatine Kinase (CK) Date and Time**

Coding Instructions: Indicate the date and time of collection of the initial CK.

Notes: Initial CK level corresponds to the first sample obtained within the first 24 hours of care. It may also have been collected at the transferring hospital.

Target Value: Any occurrence between first medical contact and 24 hours after arrival at first facility

Selections: N/A

Supporting Definition: (none)

Source: Adapted from AHA Get with the Guidelines ACTION registry

**Maximum Creatine Kinase (CK) Level**

Coding Instructions: Indicate the maximum CK level recorded during hospital stay. Target Value: Any occurrence during the entire hospital stay.

Selections: N/A

Supporting Definition: (none)

Source: Adapted from AHA Get with the Guidelines ACTION registry

**Maximum Creatine Kinase (CK) Date and Time**

Coding Instructions: Indicate the date and time of collection of the maximum CK. Target Value: Any occurrence during the entire hospital stay

Selections: N/A

Supporting Definition: (none)

Source: Adapted from AHA Get with the Guidelines ACTION registry

**Initial CK-MB Value**

Coding Instructions: Indicate the value of the initial CK-MB.

Notes: If a CK-MB value was not calculated at baseline for normal CPK results, record a value of 0 (zero). Initial CK level corresponds to the first sample obtained within the first 24 hours of care. It may also have been collected at the transferring hospital.

Target Value: Any occurrence between first medical contact and 24 hours after arrival at first facility

Selections: N/A

Supporting Definition: (none)

Source: Adapted from AHA Get with the Guidelines ACTION registry

**Initial CK-MB Date and Time**

Coding Instructions: Indicate the date and time of collection of the initial CK-MB.

Notes: Initial CK level corresponds to the first sample obtained within the first 24 hours of care. It may also have been collected at the transferring hospital.

Target Value: Any occurrence between first medical contact and 24 hours after arrival at first facility

Selections: N/A

Supporting Definition: (none)

Source: Adapted from AHA Get with the Guidelines ACTION registry

**Unit of CK-MB Level**

Coding Instructions: Indicate the sample unit of measure of CK-MB level. Target Value: N/A

Selections:

(1) IU/L (2) %

(3) (mg/mL)/IU

(4) ng/mL

Supporting Definition: (none)

Source: Adapted from AHA Get with the Guidelines ACTION registry

**Initial CK-MB Upper Limit of Normal (ULN)**

Coding Instructions: Indicate the ULN of the initial CK-MB sample.

Note(s): If a range is given for ULN values, record the highest number in the range. Examples: If the reference range given is 0.0-1.5, record ULN as 1.5. If the reference range given is < 1.5, record ULN as 1.5 as well.

The initial sample value refers to the first sample obtained within the first 24 hours of care. Target Value: N/A

Selections: N/A

Supporting Definition: (none)

Source: Adapted from AHA Get with the Guidelines ACTION registry

**Initial Troponin I Value**

Coding Instructions: Indicate the value of initial troponin I.

Notes: Initial troponin I level corresponds to the first sample obtained within the first 24 hours of care. It may also have been collected at the transferring hospital.

Target Value: Any occurrence between first medical contact and 24 hours after arrival at first facility

Selections: (1) Positive (2) Negative (3) Trace (+/-) (4) Numerical value

Supporting Definition: (none)

Source: Adapted from AHA Get with the Guidelines ACTION registry

**Unit of Troponin I**

Coding Instructions: Indicate the sample unit of measure of troponin I level. Target Value: N/A

Selections: (1) ng/mL (2) Other

Supporting Definition: (none)

Source: Adapted from AHA Get with the Guidelines ACTION registry

**Initial Troponin I Upper Reference Limit (URL)**

Coding Instructions: Indicate the URL of the initial troponin I sample. Target Value: N/A

Selections: N/A

Supporting Definition: Upper Reference Limit (URL):

Defined as the 99th percentile of troponin levels for a normal reference population.

Source: Joint ESC-ACC-AHA-WHF 2007 Task Force consensus document "Universal Definition of Myocardial Infarction"; adapted from AHA Get with the Guidelines ACTION registry

**Initial Troponin I Date and Time**

Coding Instructions: Indicate the date and time of collection of the initial troponin I.

Notes: Initial troponin I level corresponds to the first sample obtained within the first 24 hours of care. It may also have been collected at the transferring hospital.

Target Value: Any occurrence between first medical contact and 24 hours after arrival at first

facility

Selections: N/A

Supporting Definition: (none)

Source: Adapted from AHA Get with the Guidelines ACTION registry

**Maximum Troponin I Level**

Coding Instructions: Indicate the maximum troponin I level recorded during hospital stay. Target Value: Any occurrence during the entire hospital stay.

Selections: N/A

Supporting Definition: (none)

Source: Adapted from AHA Get with the Guidelines ACTION registry

**Maximum Troponin I Date and Time**

Coding Instructions: Indicate the date and time of collection of the maximum troponin I. Target Value: Any occurrence during the entire hospital stay

Selections: N/A

Supporting Definition: (none)

Source: Adapted from AHA Get with the Guidelines ACTION registry

**Initial Troponin T Value**

Coding Instructions: Indicate the value of initial troponin T.

Notes: Initial troponin T level corresponds to the first sample obtained within the first 24 hours of care. It may also have been collected at the transferring hospital.

Target Value: Any occurrence between first medical contact and 24 hours after arrival at first

facility

Selections: N/A

Supporting Definition: (none)

Source: Adapted from AHA Get with the Guidelines ACTION registry

**Unit of Troponin T**

Coding Instructions: Indicate the sample unit of measure of troponin T level. Target Value: N/A

Selections: (1) ng/mL (2) Other

Supporting Definition: (none)

Source: Adapted from AHA Get with the Guidelines ACTION registry

**Initial Troponin Upper Reference Limit (URL)**

Coding Instructions: Indicate the URL of the initial troponin T sample. Target Value: N/A

Selections: N/A

Supporting Definition: Upper Reference Limit (URL):

Defined as the 99th percentile of troponin levels for a normal reference population.

Source: Joint ESC-ACC-AHA-WHF 2007 Task Force consensus document "Universal Definition of Myocardial Infarction"

Source: Adapted from AHA Get with the Guidelines ACTION registry

**Initial Troponin T Date and Time**

Coding Instructions: Indicate the date and time of collection of the initial troponin T.

Notes: Initial troponin level corresponds to the first sample obtained within the first 24 hours of care. It may also have been collected at the transferring hospital.

Target Value: Any occurrence between first medical contact and 24 hours after arrival at first

facility

Selections: N/A

Supporting Definition: (none)

Source: Adapted from AHA Get with the Guidelines ACTION registry

**Maximum Troponin T Level**

Coding Instructions: Indicate the maximum troponin T level recorded during hospital stay. Target Value: Any occurrence during the entire hospital stay.

Selections: N/A

Supporting Definition: (none)

Source: Adapted from AHA Get with the Guidelines ACTION registry

**Maximum Troponin T Date and Time**

Coding Instructions: Indicate the date and time of collection of the maximum troponin T. Target Value: Any occurrence during the entire hospital stay

Selections: N/A

Supporting Definition: (none)

Source: Adapted from AHA Get with the Guidelines ACTION registry

**Initial Troponin (Unspecified) Value**

Coding Instructions: Indicate the value of initial troponin (unspecified).

Notes: Initial troponin (unspecified) level corresponds to the first sample obtained within the first

24 hours of care. It may also have been collected at the transferring hospital.

Target Value: Any occurrence between first medical contact and 24 hours after arrival at first facility

Selections: 1) Positive, 2) Negative 3) Numerical Value

Supporting Definition: (none)

Source: Adapted from AHA Get with the Guidelines ACTION registry

**Unit of Troponin (Unspecified)**

Coding Instructions: Indicate the sample unit of measure of troponin (unspecified) level. Target Value: N/A

Selections: (1) ng/mL (2) Other

Supporting Definition: (none)

Source: Adapted from AHA Get with the Guidelines ACTION registry

**Initial Troponin (Unspecified) Upper Reference Limit (URL)**

Coding Instructions: Indicate the URL of the initial troponin (unspecified) sample. Target Value: N/A

Selections: N/A

Supporting Definition: Upper Reference Limit (URL):

Defined as the 99th percentile of troponin levels for a normal reference population.

Source: Joint ESC-ACC-AHA-WHF 2007 Task Force consensus document "Universal Definition of Myocardial Infarction"

Source: Adapted from AHA Get with the Guidelines ACTION registry

**Initial Troponin (Unspecified) Date and Time**

Coding Instructions: Indicate the date and time of collection of the initial troponin (unspecified).

Notes: Initial troponin level corresponds to the first sample obtained within the first 24 hours of care. It may also have been collected at the transferring hospital.

Target Value: Any occurrence between first medical contact and 24 hours after arrival at first

facility

Selections: N/A

Supporting Definition: (none)

Source: Adapted from AHA Get with the Guidelines ACTION registry

**Maximum Troponin (Unspecified) Level**

Coding Instructions: Indicate the maximum troponin (unspecified) level recorded during hospital stay.

Target Value: Any occurrence during the entire hospital stay. Selections: N/A

Supporting Definition: (none)

Source: Adapted from AHA Get with the Guidelines ACTION registry

**Maximum Troponin (Unspecified) Date and Time**

Coding Instructions: Indicate the date and time of collection of the maximum troponin

(unspecified).

Target Value: Any occurrence during the entire hospital stay

Selections: N/A

Supporting Definition: (none)

Source: Adapted from AHA Get with the Guidelines ACTION registry

**Initial Hemoglobin Date and Time**

Coding Instructions: Indicate the date and time when the initial hemoglobin sample was collected (not the date results reported).

Target Value: The first value between first medical contact and discharge

Selections: (none)

Supporting Definition: (none)

Source: Adapted from AHA Get with the Guidelines ACTION registry

**Initial Hemoglobin Value**

Coding Instructions: Indicate hemoglobin value in mg/dL.

Target Value: The first value between first medical contact and discharge

Selections: (none)

Supporting Definition: (none)

Source: Adapted from AHA Get with the Guidelines ACTION registry

**Lowest Recorded Hemoglobin Date and Time**

Coding Instructions: Indicate the date and time when the hemoglobin sample with the lowest value was collected (not the date results reported).

Target Value: Any occurrence between first medical contact and discharge

Selections: (none)

Supporting Definition: (none)

Source: Adapted from AHA Get with the Guidelines ACTION registry

**Lowest Recorded Hemoglobin Value**

Coding Instructions: Indicate the lowest hemoglobin value available in mg/dL. Target Value: Any occurrence between first medical contact and discharge Selections: (none)

Supporting Definition: (none)

Source: Adapted from AHA Get with the Guidelines ACTION registry

**Last Hemoglobin Date and Time**

Coding Instructions: Indicate the date and time when the last hemoglobin sample during hospital stay was collected (not the date results reported).

Target Value: Last value prior to discharge

Selections: (none)

Supporting Definition: (none)

Source: Adapted from AHA Get with the Guidelines ACTION registry

**Last Hemoglobin Value**

Coding Instructions: Indicate the last hemoglobin value available in mg/dL. Target Value: Last value prior to discharge

Selections: (none)

Supporting Definition: (none)

Source: Adapted from AHA Get with the Guidelines ACTION registry

**Minimum Hematocrit Date and Time**

Coding Instructions: Indicate the date and time of the hematocrit sample that yielded the minimum value (not the date results reported).

Target Value: The minimum value between first medical contact and discharge

Selections: (none)

Supporting Definition: (none)

Source: Adapted from AHA Get with the Guidelines ACTION registry

**Minimum Hematocrit Value**

Coding Instructions: Indicate minimum hematocrit value in %.

Target Value: The minimum value between first medical contact and discharge

Selections: (none)

Supporting Definition: (none)

Source: Adapted from AHA Get with the Guidelines ACTION registry

**Last Recorded Hematocrit Date and Time**

Coding Instructions: Indicate the date and time when the last hematocrit sample was collected

(not the date results reported).

Target Value: Last occurrence between first medical contact and discharge

Selections: (none)

Supporting Definition: (none)

Source: Adapted from AHA Get with the Guidelines ACTION registry

**Last Recorded Hematocrit Value**

Coding Instructions: Indicate the last hematocrit value available in %. Target Value: Last occurrence between first medical contact and discharge Selections: (none)

Supporting Definition: (none)

Source: Adapted from AHA Get with the Guidelines ACTION registry

**Initial White Blood Cell (WBC) Date and Time**

Coding Instructions: Indicate the date and time when the initial WBC count was collected (not the date results reported).

Target Value: The first available value between first medical contact and discharge

Selections: (none)

Supporting Definition: (none)

Source: Adapted from VIRGO registry

**Initial WBC Value**

Coding Instructions: Record the first available WBC count (x103/µL)

Target Value: The first available value between first medical contact and discharge. Selections: (none)

Supporting Definition: (none)

Source: Adapted from VIRGO registry

**Initial Neutrophil Count Date and Time**

Coding Instructions: Indicate the date and time when the neutrophil count was collected (not the date results reported).

Target Value: The first value between first medical contact and discharge

Selections: (none)

Supporting Definition: (none) Source: Definition per CORE team

**Initial Neutrophil Ratio**

Coding Instructions: Record the initial neutrophil ratio

Target Value: The first value between first medical contact and discharge

Selections: (none)

Supporting Definition: This should be derived from the differentials of the complete blood count

(CBC)

Source: Definition per CORE team

**Initial Platelet Count Date and Time**

Coding Instructions: Indicate the date and time when the platelet count was collected (not the date results reported).

Target Value: First value between first medical contact and discharge

Selections: (none)

Supporting Definition: (none)

Source: Adapted from AHA Get with the Guidelines ACTION registry

**Initial Platelet Count Value**

Coding Instructions: Record the first available platelet count (x109/µL) Target Value: First value between first medical contact and discharge

Selections: (none)

Supporting Definition: (none)

Source: Adapted from AHA Get with the Guidelines ACTION registry

**Minimum Platelet Count Date and Time**

Coding Instructions: Indicate the date and time when the platelet count with the minimum value was collected (not the date results reported).

Target Value: Lowest value between first medical contact and discharge

Selections: (none)

Supporting Definition: (none)

Source: Adapted from AHA Get with the Guidelines ACTION registry

**Minimum Platelet Count Value**

Coding Instructions: Record the lowest available platelet count (x109/µL) Target Value: Lowest value between first medical contact and discharge

Selections: (none)

Supporting Definition: (none)

Source: Adapted from AHA Get with the Guidelines ACTION registry

**Initial LDH Date and Time**

Coding Instructions: Indicate the date and time when the initial LDH sample was collected (not the date results reported).

Target Value: First value between first medical contact and discharge

Selections: (none)

Supporting Definition: (none)

Source: Adapted from AHA Get with the Guidelines ACTION registry

**Initial LDH Value**

Coding Instructions: Record the first available LDH value (mg/dL) Target Value: First value between first medical contact and discharge Selections: (none)

Supporting Definition: (none)

Source: Adapted from AHA Get with the Guidelines ACTION registry

**Initial Blood Glucose Value**

Coding Instructions: Record the first available blood glucose value (mg/dL) Target Value: First value between first medical contact and discharge Selections: (none)

Supporting Definition: (none)

Source: Adapted from AHA Get with the Guidelines ACTION registry

**Initial AST Level Date and Time**

Coding Instructions: Indicate the date and time when the AST levels were collected (not the date results reported).

Target Value: First value between first medical contact and discharge

Selections: (none)

Supporting Definition: (none) Source: Definition per CORE team

**Initial AST level Value**

Coding Instructions: Record the first available AST level. (IU/L)

Target Value: First value between first medical contact and discharge

Selections: (none)

Supporting Definition: (none) Source: Definition per CORE team

**Maximum AST Level Date and Time**

Coding Instructions: Indicate the date and time when the maximum AST levels were collected

(not the date results reported).

Target Value: Maximum value between first medical contact and discharge

Selections: (none)

Supporting Definition: (none) Source: Definition per CORE team

**Maximum AST Level Value**

Coding Instructions: Record the maximum available AST level. (IU/L) Target Value: Maximum value between first medical contact and discharge Selections: (none)

Supporting Definition: (none) Source: Definition per CORE team

**Minimum AST Level Date and Time**

Coding Instructions: Indicate the date and time when the minimum AST levels were collected

(not the date results reported).

Target Value: Minimum value between first medical contact and discharge

Selections: (none)

Supporting Definition: (none) Source: Definition per CORE team

**Minimum AST Level Value**

Coding Instructions: Record the minimum available AST level. (IU/L) Target Value: Minimum value between first medical contact and discharge Selections: (none)

Supporting Definition: (none) Source: Definition per CORE team

**Initial ALT Level Date and Time**

Coding Instructions: Indicate the date and time when the initial ALT levels were collected (not the date results reported).

Target Value: First value between first medical contact and discharge

Selections: (none)

Supporting Definition: (none) Source: Definition per CORE team

**Initial ALT level Value**

Coding Instructions: Record the first available ALT level. (IU/L)

Target Value: First value between first medical contact and discharge

Selections: (none)

Supporting Definition: (none) Source: Definition per CORE team

**Maximum ALT Level Date and Time**

Coding Instructions: Indicate the date and time when the maximum ALT levels were collected

(not the date results reported).

Target Value: Maximum value between first medical contact and discharge

Selections: (none)

Supporting Definition: (none) Source: Definition per CORE team

**Maximum ALT Level Value**

Coding Instructions: Record the maximum available ALT level. (IU/L) Target Value: Maximum value between first medical contact and discharge Selections: (none)

Supporting Definition: (none)

Source: Definition per CORE team

**Minimum ALT Level Date and Time**

Coding Instructions: Indicate the date and time when the minimum ALT levels were collected

(not the date results reported).

Target Value: Minimum value between first medical contact and discharge

Selections: (none)

Supporting Definition: (none) Source: Definition per CORE team

**Minimum ALT Level Value**

Coding Instructions: Record the minimum available ALT level. (IU/L) Target Value: Minimum value between first medical contact and discharge Selections: (none)

Supporting Definition: (none) Source: Definition per CORE team

**Initial Total Bilirubin Date and Time**

Coding Instructions: Indicate the date and time when the initial total bilirubin levels were collected (not the date results reported).

Target Value: First value between first medical contact and discharge

Selections: (none)

Supporting Definition: (none) Source: Definition per CORE team

**Initial Total Bilirubin Value**

Coding Instructions: Record the first available total bilirubin level (IU/L). Target Value: First value between first medical contact and discharge Selections: (none)

Supporting Definition: (none)

Source: Definition per CORE team

**Initial Direct Bilirubin Date and Time**

Coding Instructions: Indicate the date and time when the initial direct bilirubin levels were collected (not the date results reported).

Target Value: First value between first medical contact and discharge

Selections: (none)

Supporting Definition: (none) Source: Definition per CORE team

**Initial Direct Bilirubin Value**

Coding Instructions: Record the first available direct bilirubin level (IU/L). Target Value: First value between first medical contact and discharge Selections: (none)

Supporting Definition: (none) Source: Definition per CORE team

**Initial Creatinine Date and Time**

Coding Instructions: Indicate the date and time when the initial creatinine levels were collected

(not the date results reported).

Target Value: First value between first medical contact and discharge

Selections: (none)

Supporting Definition: (none)

Source: Adapted from AHA Get with the Guidelines ACTION registry

**Initial Creatinine Value**

Coding Instructions: Record the first available creatinine level (mg/dL). Target Value: First value between first medical contact and discharge Selections: (none)

Supporting Definition: (none)

Source: Adapted from AHA Get with the Guidelines ACTION registry

**Maximum Creatinine Date and Time**

Coding Instructions: Indicate the date and time when the creatinine levels with maximum value were collected (not the date results reported).

Target Value: Maximum occurrence between first medical contact and discharge

Selections: (none)

Supporting Definition: (none)

Source: Adapted from AHA Get with the Guidelines ACTION registry

**Maximum Creatinine Value**

Coding Instructions: Record the maximum available creatinine level. (mg/dL) Target Value: Maximum occurrence between first medical contact and discharge Selections: (none)

Supporting Definition: (none)

Source: Adapted from AHA Get with the Guidelines ACTION registry

**Last Creatinine Date and Time**

Coding Instructions: Indicate the date and time when the last creatinine levels were collected

(not the date results reported).

Target Value: Last available value between the first medical contact and discharge

Selections: (none)

Supporting Definition: (none)

Source: Adapted from AHA Get with the Guidelines ACTION registry

**Last Creatinine Value**

Coding Instructions: Record the last available creatinine level. (mg/dL)

Target Value: Last available value between the first medical contact and discharge

Selections: (none)

Supporting Definition: (none)

Source: Adapted from AHA Get with the Guidelines ACTION registry

**Unit of BUN**

Coding Instructions: indicate the unit of BUN. Target Value: N/A

Selections: (1) mg/dl (2) mmol/L (3) Other

Supporting Definition: (none) Source: Definition per CORE team.

**Initial BUN Date and Time**

Coding Instructions: Indicate the date and time when the initial BUN levels were collected (not the date results reported).

Target Value: First value between first medical contact and discharge

Selections: (none)

Supporting Definition: (none)

Source: Adapted from AHA Get with the Guidelines ACTION registry

**Initial BUN Value**

Coding Instructions: Record the first available BUN level.

Target Value: First value between first medical contact and discharge

Selections: (none)

Supporting Definition: (none)

Source: Adapted from AHA Get with the Guidelines ACTION registry

**Maximum BUN Date and Time**

Coding Instructions: Indicate the date and time when the maximum BUN levels were collected

(not the date results reported).

Target Value: Maximum value between first medical contact and discharge

Selections: (none)

Supporting Definition: (none)

Source: Adapted from AHA Get with the Guidelines ACTION registry

**Maximum BUN Value**

Coding Instructions: Record the maximum BUN value

Target Value: Maximum value between first medical contact and discharge

Selections: (none)

Supporting Definition: (none)

Source: Adapted from AHA Get with the Guidelines ACTION registry

**Initial B-type Natriuretic Peptide (BNP) Date and Time**

Coding Instructions: Indicate the date and time when the initial BNP levels were collected (not the date results reported).

Target Value: First value between first medical contact and discharge

Selections: (none)

Supporting Definition: (none)

Source: Adapted from AHA Get with the Guidelines ACTION registry

**Initial BNP Value**

Coding Instructions: Record the first available BNP level (pg/ml). Target Value: First value between first medical contact and discharge Selections: (none)

Supporting Definition: (none)

Source: Adapted from AHA Get with the Guidelines ACTION registry

**Unit of BNP/NT-proBNP**

Coding Instructions: indicate the unit of BNP or NT-proBNP. Target Value: N/A

Selections: 1) Pg/mL, 2) Ug/L, 3) Ug/mL, 4) Fmol/L, 5) other

Supporting Definition: (none) Source: Definition per CORE team.

**Initial N-terminal proBNP (NT-BNP) Date and Time**

Coding Instructions: Indicate the date and time when the initial NT-BNP levels were collected

(not the date results reported).

Target Value: First value between first medical contact and discharge

Selections: (none)

Supporting Definition: (none)

Source: Adapted from AHA Get with the Guidelines ACTION registry

**Initial NT-BNP Value**

Coding Instructions: Record the first available NT-BNP level. (pg/ml) Target Value: First value between first medical contact and discharge Selections: (none)

Supporting Definition: (none)

Source: Adapted from AHA Get with the Guidelines ACTION registry

**Lipid Panel Date and Time**

Coding Instructions: Indicate the date and time the sample was collected (not the date results reported).

Note(s): If multiple lipid samples were collected, the first one should be preferentially abstracted.

Selections: (none)

Supporting Definition: (none)

Source: Adapted from AHA Get with the Guidelines ACTION registry

**Unit of Lipids**

Coding Instructions: indicate the unit of lipids. Target Value: N/A

Selections: (1) mg/dl (2) mmol/L (3) Other

Supporting Definition: (none) Source: Definition per CORE team.

**Total Cholesterol Value**

Coding Instructions: Indicate the total cholesterol value in mg/dL.

Notes: If multiple total cholesterol samples were collected, the first one should be preferentially abstracted.

Selections: (none)

Supporting Definition: (none)

Source: Adapted from AHA Get with the Guidelines ACTION registry

**HDL Cholesterol Value**

Coding Instructions: Indicate the HDL cholesterol value in mg/dL.

Notes: If multiple HDL samples were collected, the first one should be preferentially abstracted. Selections: (none)

Supporting Definition: (none)

Source: Adapted from AHA Get with the Guidelines ACTION registry

**LDL Cholesterol Value**

Coding Instructions: Indicate the LDL cholesterol value in mg/dL.

Notes: If multiple LDL samples were collected, the first one should be preferentially abstracted. Selections: (none)

Supporting Definition: (none)

Source: Adapted from AHA Get with the Guidelines ACTION registry

**Triglycerides Cholesterol Value**

Coding Instructions: Indicate the triglycerides cholesterol value in mg/dL.

Notes: If multiple triglyceride samples were collected, the first one should be preferentially abstracted.

Selections: (none)

Supporting Definition: (none)

Source: Adapted from AHA Get with the Guidelines ACTION registry

**Urine Protein Date and Time**

Coding Instructions: Indicate the date and time when the urine protein levels were collected (not the date results reported).

Target Value: Any occurrence between the first medical contact and discharge

Selections: (none)

Supporting Definition: (none)

Source: Adapted from VIRGO registry

**Urine Protein Value**

Coding Instructions: Indicate the value of urine protein levels.

Target Value: Any occurrence between the first medical contact and discharge Selections: (1) Positive/+ (2) Negative (3) Trace (+/-) (4) ++ (5) +++ (6) ++++ Supporting Definition: (none)

Source: Adapted from VIRGO registry

**Initial International Normalized Ratio (INR) Date and Time**

Coding Instructions: Indicate the date and time when the initial INR levels were collected (not the date results reported).

Target Value: First value between first medical contact and discharge

Selections: (none)

Supporting Definition: (none)

Source: Adapted from AHA Get with the Guidelines ACTION registry

**Initial INR Value**

Coding Instructions: Record the first available INR value.

Target Value: First value between first medical contact and discharge

Selections: (none)

Supporting Definition: (none)

Source: Adapted from AHA Get with the Guidelines ACTION registry

**Initial Activated Partial Prothrombin Time (APTT) Date and Time**

Coding Instructions: Indicate the date and time when the initial APTT levels were collected (not the date results reported).

Target Value: First value between first medical contact and discharge

Selections: (none)

Supporting Definition: (none)

Source: Adapted from AHA Get with the Guidelines ACTION registry

**Initial APTT Value**

Coding Instructions: Record the first available APTT value.

Target Value: First value between first medical contact and discharge

Selections: (none)

Supporting Definition: (none)

Source: Adapted from AHA Get with the Guidelines ACTION registry

**Initial Prothrombin Time (PT) Date and Time**

Coding Instructions: Indicate the date and time when the initial PT levels were collected (not the date results reported).

Target Value: First value between first medical contact and discharge

Selections: (none)

Supporting Definition: (none)

Source: Adapted from AHA Get with the Guidelines ACTION registry

**Initial PT Value**

Coding Instructions: Record the first available PT value.

Target Value: First value between first medical contact and discharge

Selections: (none)

Supporting Definition: (none)

Source: Adapted from AHA Get with the Guidelines ACTION registry

**Initial Potassium Date and Time**

Coding Instructions: Indicate the date and time when the initial potassium levels were collected

(not the date results reported).

Target Value: First value between first medical contact and discharge

Selections: (none)

Supporting Definition: (none)

Source: Adapted from AHA Get with the Guidelines ACTION registry

**Initial Potassium Value**

Coding Instructions: Record the first available potassium value in mEq/L or mmol/L. Target Value: First value between first medical contact and discharge

Selections: (none)

Supporting Definition: (none)

Source: Adapted from AHA Get with the Guidelines ACTION registry

**Maximum Potassium Date and Time**

Coding Instructions: Indicate the date and time when the maximum potassium levels were collected (not the date results reported).

Target Value: Maximum value between first medical contact and discharge

Selections: (none)

Supporting Definition: (none)

Source: Adapted from AHA Get with the Guidelines ACTION registry

**Maximum Potassium Value**

Coding Instructions: Record the maximum available potassium value in mEq/L or mmol/L. Target Value: Maximum value between first medical contact and discharge

Selections: (none)

Supporting Definition: (none)

Source: Adapted from AHA Get with the Guidelines ACTION registry

**HBsAg**

Coding Instructions: Indicate if the patient was tested for HBsAg antigen or Pre-S1 protein. Target Value: Any occurrence between arrival at admitting hospital and discharge Selections: (1) No (2) Yes

If Yes, specify the result:

Negative Positive Unrecorded

Supporting Definition: HBsAg is the surface antigen of the hepatitis B virus (HBV). It indicates current hepatitis B infection.

Source: Definition per CORE team

**HBsAb**

Coding Instructions: Indicate if the patient was tested for HBsAb.

Target Value: Any occurrence between arrival at admitting hospital and discharge

Selections: (1) No (2) Yes If Yes, specify the result: Negative

Positive

Unrecorded

Note: If the numerical value is given, please report it.

Supporting Definition: HBsAb is the antibody directed against the surface antigen of the hepatitis B virus (HBV).

Source: Definition per CORE team

**HBeAg /Anti HBeAg**

Coding Instructions: Indicate if the patient was tested for HBeAg antigen or Anti-HBeAg antibody.

Target Value: Any occurrence between arrival at admitting hospital and discharge. Selections: (1) No (2) Yes

If Yes, specify the result: Negative

Positive

Unrecorded

Supporting Definition: HBeAg is an antigen of the hepatitis B virus indicating active replication of virus in the bloodstream. It indicates current hepatitis B infection.

Source: Definition per CORE team

**HCV-Ab**

Coding Instructions: Indicate if the patient was tested for HCV antibody.

Target Value: Any occurrence between arrival at admitting hospital and discharge

Selections: (1) No (2) Yes If Yes, specify the result: Negative

Positive

Unrecorded

Supporting Definition: HCV antibody test helps to detect infection with Hepatitis C virus. If tested for HCV-IgM, HCV-IgG, or both, the answer is Yes.

Source: Definition per CORE team

**HIV Ab**

Coding Instructions: Indicate if the patient was tested for HIV antibody.

Target Value: Any occurrence between arrival at admitting hospital and discharge

Selections: (1) No (2) Yes If Yes, specify the result: Negative

Positive

Unrecorded

Supporting Definition: HIV antibody test helps to detect infection with Human Immunodeficiency

Virus.

Source: Definition per CORE team

**Initial C-reactive protein (CRP) Date and Time**

Coding Instructions: Indicate the date and time when the initial CRP levels were collected (not the date results reported).

Target Value: First occurrence between the first medical contact and discharge

Selections: (none)

Supporting Definition: (none) Source: Definition per CORE team

**Initial CRP Value**

Coding Instructions: Indicate the result of test for initial CRP.

Target Value: First occurrence between the first medical contact and discharge

Selections: (none)

Supporting Definition: (none) Source: Definition per CORE team

**Unit of CRP/hs-CRP**

Coding Instructions: indicate the unit of CRP or hs-CRP. Target Value: N/A

Selections: (1) mg/dl (2) Pg/mL (3) Other

Supporting Definition: (none) Source: Definition per CORE team.

**Initial High Sensitivity C-reactive Protein (hs-CRP) Date and Time**

Coding Instructions: Indicate the date and time when the initial hs-CRP levels were collected

(not the date results reported).

Target Value: First occurrence between the first medical contact and discharge

Selections: (none) Supporting Definition: (none) Source: Definition per CORE team

**Initial hs-CRP Value**

Coding Instructions: Indicate the result of test for initial hs-CRP.

Target Value: First occurrence between the first medical contact and discharge

Selections: (none)

Supporting Definition: (none) Source: Definition per CORE team

**MEDICATIONS ADMINISTERED**

**Note:** All the information about medications was entered into a central medication database. For each medication, we abstracted name, dose, and route of administration. The following definitions describe specific pre-defined questions pertinent to medication administration for acute myocardial infarction.

**Aspirin in the First 24 Hours**

Coding Instructions: Indicate if aspirin was administered in the first 24 hours before or after first medical contact, regardless of location of care (e.g. transferring facility or EMS).

Target Value: Any occurrence between 24 hours before first medical contact and 24 hours after first medical contact

Selections: (1) No (2) Yes

Supporting Definitions: (none)

Source: Adapted from VIRGO registry

**Documented Reasons for Non-prescription of Aspirin in the First 24 Hours**

Coding Instructions: If the medication was not prescribed, indicate whether the reason was documented.

Target Value: Any documentation in the chart regarding contraindications for the prescription of

the medication in the first 24 hours. Selections:

(1) No

(2) Yes – Allergy

(3) Yes – Other (specify) Source: Definition per CORE team

**Aspirin in the First 24 Hours- Start Date**

Coding Instructions: Indicate the date that aspirin was administered in the first 24 hours before or after first medical contact, regardless of location of care (e.g. transferring facility or EMS). If administered more than once, code the first date/time it was administered.

Target Value: The first value between 24 hours before first medical contact and 24 hours after first medical contact.

Selections: (none)

Supporting Definitions: (none)

Source: Adapted from VIRGO registry

**Aspirin in the First 24 Hours- Start Time**

Coding Instructions: Indicate the time that aspirin was administered in the first 24 hours before or after first medical contact, regardless of location of care (e.g. transferring facility or EMS). If administered more than once, code the first date/time it was administered.

Target Value: The first value between 24 hours before first medical contact and 24 hours after first medical contact.

Selections: (none)

Supporting Definitions: (none)

Source: Adapted from VIRGO registry

**Aspirin in the First 24 Hours- Dose**

Coding Instructions: Indicate the cumulative dose of aspirin.

Target Value: The first value between 24 hours before first medical contact and 24 hours after first medical contact.

Selections: (none)

Supporting Definitions: (none)

Source: Adapted from AHA Get with the Guidelines ACTION registry

**Aspirin at Discharge**

Coding Instructions: Indicate if aspirin was continued or prescribed.

Note(s): Discharge medications do not need to be recorded for patients who were discharged to

"Other acute care hospital", "Hospice", or "Left against medical advice (AMA)." Target Value: Any occurrence on discharge

Selections: (1) No (2) Yes

Supporting Definitions: (none)

Source: Adapted from AHA Get with the Guidelines ACTION registry

**Aspirin at Discharge–Dose**

Coding Instructions: Indicate the dose of aspirin prescribed at discharge.

Note(s): Discharge medications do not need to be recorded for patients who were discharged to

"Other acute care hospital", "Hospice", or "Left against medical advice (AMA)." Target Value: The highest value on discharge

Selections: (none)

Supporting Definitions: (none)

Source: Adapted from AHA Get with the Guidelines ACTION registry

**Documented Reasons for Non-Prescription of Aspirin at Discharge**

Coding Instructions: If the medication was not prescribed, indicate whether the reason was documented.

Target Value: Any documentation in the chart regarding contraindications for the prescription of the medication at Discharge.

Selections: (1) No

(2) Yes – Allergy

(3) Yes – Other (specify)

Source: Definition per CORE team

**Warfarin during the First 24 Hours**

Coding Instructions: Indicate if warfarin was administered in the first 24 hours before or after first medical contact, regardless of location of care (e.g. transferring facility or EMS).

Note(s): Discharge medications do not need to be recorded for patients who were discharged to

"Other acute care hospital", "Hospice", or "Left against medical advice (AMA)."

Target Value: The first value between 24 hours before first medical contact and 24 hours after first medical contact.

Selections: (1) No (2) Yes

Supporting Definitions: (none)

Source: Adapted from VIRGO registry

**Warfarin at Discharge**

Coding Instructions: Indicate if Warfarin was continued or prescribed.

Note(s): Discharge medications do not need to be recorded for patients who were discharged to

"Other acute care hospital", "Hospice", or "Left against medical advice (AMA)." Target Value: Any occurrence on discharge

Selections: (1) No (2) Yes

Supporting Definitions: (none)

Source: Adapted from AHA Get with the Guidelines ACTION registry

**Clopidogrel in the First 24 Hours**

Coding Instructions: Indicate if clopidogrel was administered, regardless of location of care (e.g. transferring facility or EMS).

Target Value: Any occurrence between 24 hours before first medical contact and 24 hours after

first medical contact

Selections: (1) No (2) Yes

Supporting Definitions: (none)

Source: Adapted from AHA Get with the Guidelines ACTION registry

**Clopidogrel in the First 24 Hours- Start Date**

Coding Instructions: Indicate the date the initial dose of clopidogrel was administered, regardless of location of care (e.g. transferring facility or EMS). If administered more than once, code the first date/time it was administered.

Target Value: The first value between 24 hours before first medical contact and 24 hours after first medical contact.

Selections: (none)

Supporting Definitions: (none)

Source: Adapted from AHA Get with the Guidelines ACTION registry

**Clopidogrel in the First 24 Hours- Start Time**

Coding Instructions: Indicate the time that clopidogrel was administered in the first 24 hours before or after first medical contact, regardless of location of care (e.g. transferring facility or EMS). If administered more than once, code the first date/time it was administered.

Target Value: The first value for clopidogrel in First 24 Hours Start Date

Selections: (none)

Supporting Definitions: (none)

Source: Adapted from AHA Get with the Guidelines ACTION registry

**Clopidogrel in the First 24 Hours- Dose**

Coding Instructions: Indicate the cumulative dose of clopidogrel.

Target Value: The cumulative dose between first medical contact and 24 hours after first medical contact

Selections: (none)

Supporting Definitions: (none)

Source: Adapted from AHA Get with the Guidelines ACTION registry

**Clopidogrel at Discharge**

Coding Instructions: Indicate if clopidogrel was continued or prescribed at discharge.

Note(s): Discharge medications do not need to be recorded for patients who were discharged to

"Other acute care hospital", "Hospice", or "Left against medical advice (AMA)." Target Value: Any occurrence on discharge

Selections: (1) No (2) Yes

Supporting Definitions: (none)

Source: Adapted from AHA Get with the Guidelines ACTION registry

**Clopidogrel at Discharge–Dose**

Coding Instructions: Indicate the dose of clopidogrel prescribed at discharge.

Note(s): Discharge medications do not need to be recorded for patients who were discharged to

"Other acute care hospital", "Hospice", or "Left against medical advice (AMA)." Target Value: The highest value on discharge

Selections: (none)

Supporting Definitions: (none)

Source: Adapted from AHA Get with the Guidelines ACTION registry

**Ticlopidine in the First 24 Hours**

Coding Instructions: Indicate if ticlopidine was administered, regardless of location of care (e.g.

transferring facility or EMS).

Target Value: Any occurrence between 24 hours before first medical contact and 24 hours after first medical contact

Selections: (1) No (2) Yes

Supporting Definitions: (none)

Source: Adapted from AHA Get with the Guidelines ACTION registry

**Ticlopidine in the First 24 Hours-Start Date**

Coding Instructions: Indicate the date the initial dose of ticlopidine was administered, regardless of location of care (e.g. transferring facility or EMS). If administered more than once, code the first date/time it was administered.

Target Value: The first value between 24 hours before first medical contact and 24 hours after first medical contact

Selections: (none)

Supporting Definitions: (none)

Source: Adapted from AHA Get with the Guidelines ACTION registry

**Ticlopidine in the First 24 Hours- Start Time**

Coding Instructions: Indicate the time that ticlopidine was administered in the first 24 hours before or after first medical contact, regardless of location of care (e.g. transferring facility or EMS). If administered more than once, code the first date/time it was administered.

Target Value: The first value between 24 hours before first medical contact and 24 hours after first medical contact

Selections: (none)

Supporting Definitions: (none)

Source: Adapted from AHA Get with the Guidelines ACTION registry

**Ticlopidine in the First 24 Hours- Dose**

Coding Instructions: Indicate the cumulative dose of ticlopidine.

Target Value: The cumulative dose between first medical contact and 24 hours after first medical contact

Selections: (none)

Supporting Definitions: (none)

Source: Adapted from AHA Get with the Guidelines ACTION registry

**Ticlopidine at Discharge**

Coding Instructions: Indicate if ticlopidine was continued or prescribed at discharge.

Note(s): Discharge medications do not need to be recorded for patients who were discharged to

"Other acute care hospital", "Hospice", or "Left against medical advice (AMA)." Target Value: Any occurrence on discharge

Selections: (1) No (2) Yes

Supporting Definitions: (none)

Source: Adapted from AHA Get with the Guidelines ACTION registry

**Ticlopidine at Discharge –Dose**

Coding Instructions: Indicate the dose of ticlopidine prescribed at discharge.

Note(s): Discharge medications do not need to be recorded for patients who were discharged to

"Other acute care hospital", "Hospice", or "Left against medical advice (AMA)." Target Value: The highest value on discharge

Selections: (none)

Supporting Definitions: (none)

Source: Adapted from AHA Get with the Guidelines ACTION registry

**Prasugrel in the First 24 Hours**

Coding Instructions: Indicate if prasugrel was administered, regardless of location of care (e.g. transferring facility or EMS).

Target Value: Any occurrence between 24 hours before first medical contact and 24 hours after first medical contact

Selections: (1) No (2) Yes

Supporting Definitions: (none)

Source: Adapted from AHA Get with the Guidelines ACTION registry

**Prasugrel in the First 24 Hours- Start Date**

Coding Instructions: Indicate the date the initial dose of prasugrel was administered, regardless of location of care (e.g. transferring facility or EMS). If administered more than once, code the first date/time it was administered.

Target Value: The first value between 24 hours before first medical contact and 24 hours after first medical contact

Selections: (none)

Supporting Definitions: (none)

Source: Adapted from AHA Get with the Guidelines ACTION registry

**Prasugrel in the First 24 Hours- Start Time**

Coding Instructions: Indicate the time that prasugrel was administered in the first 24 hours before or after first medical contact, regardless of location of care (e.g. transferring facility or EMS). If administered more than once, code the first date/time it was administered.

Target Value: The first value between 24 hours before first medical contact and 24 hours after first medical contact.

Selections: (none)

Supporting Definitions: (none)

Source: Adapted from AHA Get with the Guidelines ACTION registry

**Prasugrel in the First 24 Hours- Dose**

Coding Instructions: Indicate the cumulative dose of prasugrel.

Target Value: The cumulative dose between first medical contact and 24 hours after first medical contact

Selections: (none)

Supporting Definitions: (none)

Source: Adapted from AHA Get with the Guidelines ACTION registry

**Prasugrel at Discharge**

Coding Instructions: Indicate if prasugrel was continued or prescribed at discharge.

Note(s): Discharge medications do not need to be recorded for patients who were discharged to

"Other acute care hospital", "Hospice", or "Left against medical advice (AMA)." Target Value: Any occurrence on discharge

Selections: (1) No (2) Yes

Supporting Definitions: (none)

Source: Adapted from AHA Get with the Guidelines ACTION registry

**Prasugrel at Discharge–Dose**

Coding Instructions: Indicate the dose of prasugrel prescribed at discharge.

Note(s): Discharge medications do not need to be recorded for patients who were discharged to

"Other acute care hospital", "Hospice", or "Left against medical advice (AMA)." Target Value: The highest value on discharge

Selections: (none)

Supporting Definitions: (none)

Source: Adapted from AHA Get with the Guidelines ACTION registry

**Fibrinolysis in the First 24 Hours**

Coding Instructions: Indicate if fibrinolytics were administered, regardless of location of care

(e.g. transferring facility or EMS).

Target Value: Any occurrence between 24 hours before first medical contact and 24 hours after first medical contact

Selections: (1) No (2) Yes

Supporting Definitions: If fibrinolytics are used, please name the fibrinolytic agent, the Bolus dose (IU), the Second Bolus dose (IU/hour), or Maintenance dose (IU/hour), where applicable, or mark “Unknown” if doses are not documented.

Source: Adapted from VIRGO registry

**Refusal of Fibrinolysis**

Coding Instructions: Indicate if there is documentation of patient refusal for fibrinolysis Target Value: Any occurrence from the beginning of hospital stay to discharge Selections: (1) No (2) Yes

Supporting Definitions: (none) Source: Definition per CORE team

**Fibrinolysis in the First 24 Hours –Start Date**

Coding Instructions: Indicate the date the initial dose of fibrinolytic administered, regardless of location of care (e.g. transferring facility or EMS). If administered more than once, code the first date/time it was administered.

Target Value: The first value between 24 hours before first medical contact and 24 hours after first medical contact

Selections: (none)

Supporting Definitions: (none)

Source: Adapted from AHA Get with the Guidelines ACTION registry

**Fibrinolysis in the First 24 Hours –Start Time**

Coding Instructions: Indicate the time that a fibrinolytic was administered in the first 24 hours before or after first medical contact, regardless of location of care (e.g. transferring facility or EMS). If administered more than once, code the first date/time it was administered.

Target Value: The first value between 24 hours before first medical contact and 24 hours after first medical contact.

Selections: (none)

Supporting Definitions: (none)

Source: Adapted from AHA Get with the Guidelines ACTION registry

**Documented Reasons for Non-prescription of Fibrinolysis in the First 24 Hours** Coding Instructions: If the medication was not prescribed, indicate whether the reason was documented.

Target Value: Any documentation in the chart regarding contraindications for the prescription of the medication in the first 24 hours.

Selections: (1) No (2) Yes – Allergy (3) Yes – Other (specify)

Source: Definition per CORE team

**Fibrinolysis after the First 24 Hours**

Coding Instructions: Indicate if fibrinolytics were administered, regardless of location of care

(e.g. transferring facility or EMS).

Target Value: Any occurrence after the first 24 hours from the first medical contact

Selections: (1) No (2) Yes

Supporting Definitions: (none)

Source: Adapted from AHA Get with the Guidelines ACTION registry

**Fibrinolysis after the First 24 Hours–Start Date**

Coding Instructions: Indicate the date the initial dose of fibrinolytic was administered, regardless of location of care (e.g. transferring facility or EMS). If administered more than once, code the first date/time it was administered.

Target Value: The first value after the first 24 hours from the first medical contact

Selections: (none)

Supporting Definitions: (none)

Source: Adapted from AHA Get with the Guidelines ACTION registry

**Fibrinolysis after the First 24 Hours –Start Time**

Coding Instructions: Indicate the time that a fibrinolytic was administered in the first 24 hours before or after first medical contact, regardless of location of care (e.g. transferring facility or EMS). If administered more than once, code the first date/time it was administered.

Target Value: The first value after the first 24 hours from the first medical contact

Selections: (none)

Supporting Definitions: (none)

Source: Adapted from AHA Get with the Guidelines ACTION registry

**Anticoagulant Use**

Coding Instructions: First, please indicate if an anticoagulant is being used or not (YES/ NO). Next, please specify the type of anticoagulant(s) administered, including: coumarins, unfractionated heparin, low molecular weight heparins (enoxaparin, dalteparin, or fondaparinux), direct thrombin inhibitors (such as lepirudin, bivalirudin, and argatroban). Please indicate the start date and time of first administration of the anticoagulant medication(s).

For anticoagulants that are used in the first 24 hours from first medical contact, depending on

the type of the anticoagulant, please also specify:

(1) Was a bolus used? If yes, provide the date, time and dose.

(2) Was an infusion used? If yes, provide the date, time and dose.

(3) Was a subcutaneous injection used? If yes, provide the date, time and dose. (4) Provide the number of daily injections where applicable.

Source: Adapted from VIRGO registry

**Glycoprotein IIb/IIIa Inhibitor Use**

Coding Instructions: First, please indicate if glycoprotein IIb/IIIa inhibitors were used (YES/ NO). Next please specify the data and time of first administration and the name of drug being used: abciximab, tirofiban or eptifibatide. If used during the first 24 hours from the time of first medical contact, please indicate the date and time, as well as bolus and maintenance doses.

Source: Adapted from VIRGO registry

**Beta-blockers in the First 24 Hours**

Coding Instructions: Indicate if a beta-blocker was administered, regardless of location of care

(e.g., transferring facility or EMS).

Target Value: Any occurrence between 24 hours before first medical contact and 24 hours after first medical contact

Selections: (1) No (2) Yes

Supporting Definitions: (none)

Source: Adapted from AHA Get with the Guidelines ACTION registry

**Documented Reasons for Non-prescription of Beta-blocker in the First 24 Hours**

Coding Instructions: Indicate if the patient has documented reasons for not being prescribed a beta-blocker in the first 24 hours.

Target Value: N/A

Selections: (1) Yes allergy (2) Yes other (3) No

Supporting Definitions: (none)

Note(s): Code 'yes' if there is documented reason that the patient was started on an oral form of a beta-blocker within the first 24 hours.

Code 'no' if a there is no documented reason that the patient was given a sublingual, IV, or short acting formula of one of these medications within the first 24 hours.

Source: Adapted from AHA Get with the Guidelines ACTION registry

**Beta-blockers in the First 24 Hours- Start Date**

Coding Instructions: Indicate the date the initial dose of beta-blocker was administered, regardless of location of care (e.g. transferring facility or EMS). If administered more than once, code the first date/time it was administered.

Target Value: The first value between 24 hours before first medical contact and 24 hours after

first medical contact

Selections: (none)

Supporting Definitions: (none)

Source: Adapted from AHA Get with the Guidelines ACTION registry

**Beta Blockers in the First 24 Hours-Start Time**

Coding Instructions: Indicate the time that the beta blocker was administered in the first 24

hours before or after first medical contact, regardless of location of care (e.g., transferring facility or EMS). If administered more than once, code the first date/time it was administered.

Target Value: The first value between 24 hours before first medical contact and 24 hours after first medical contact.

Selections: (none)

Supporting Definitions: (none)

Source: Adapted from AHA Get with the Guidelines ACTION registry

**Beta Blockers in the First 24 Hours- Route of Administration**

Coding Instructions: Indicate if beta blockers were given intravenous, PO, or both. Target Value: N/A

Selections: (1) IV (2) PO (3) Both Supporting Definitions: (none) Source: Definition per CORE team

**Beta Blockers at Discharge**

Coding Instructions: Indicate if a beta blocker was continued or prescribed at discharge.

Note(s): Discharge medications do not need to be recorded for patients who were discharged to

"Other acute care hospital", "Hospice", or "Left against medical advice (AMA)." Target Value: Any occurrence on discharge

Selections: (1) No (2) Yes

Supporting Definitions: (none)

Source: Adapted from AHA Get with the Guidelines ACTION registry

**Beta Blockers at Discharge–Dose**

Coding Instructions: Indicate the dose of beta blockers prescribed at discharge.

Note(s): Discharge medications do not need to be recorded for patients who were discharged to

"Other acute care hospital", "Hospice", or "Left against medical advice (AMA)." Target Value: The highest value on discharge

Selections: (none)

Supporting Definitions: (none)

Source: Adapted from AHA Get with the Guidelines ACTION registry

**Documented Reasons for Non-prescription of Beta Blockers at Discharge**

Coding Instructions: If the medication was not prescribed, indicate whether the reason was documented.

Target Value: Any documentation in the chart regarding contraindications for the prescription of the medication at discharge.

Selections: (1) No (2) Yes – Allergy (3) Yes – Other (specify) Source: Definition per CORE team

**Angiotensin Converting Enzyme Inhibitor in the First 24 Hours**

Coding Instructions: Indicate if an angiotensin converting enzyme inhibitor was administered, regardless of location of care (e.g., transferring facility or EMS).

Target Value: Any occurrence between 24 hours before first medical contact and 24 hours after first medical contact

Selections: (1) No (2) Yes

Supporting Definitions: (none)

Source: Adapted from AHA Get with the Guidelines ACTION registry

**Angiotensin Converting Enzyme Inhibitors in the First 24 Hours- Start Date**

Coding Instructions: Indicate the date the initial dose of angiotensin converting enzyme inhibitor was administered, regardless of location of care (e.g. transferring facility or EMS). If administered more than once, code the first date/time it was administered.

Target Value: The first value between 24 hours before first medical contact and 24 hours after

first medical contact. Selections: (none)

Supporting Definitions: (none)

Source: Adapted from AHA Get with the Guidelines ACTION registry

**Angiotensin Converting Enzyme Inhibitors in the First 24 Hours- Start Time**

Coding Instructions: Indicate the time that the angiotensin converting enzyme inhibitor was administered in the first 24 hours before or after first medical contact, regardless of location of care (e.g. transferring facility or EMS). If administered more than once, code the first date/time it was administered.

Target Value: The first value between 24 hours before first medical contact and 24 hours after first medical contact.

Selections: (none)

Supporting Definitions: (none)

Source: Adapted from AHA Get with the Guidelines ACTION registry

**Angiotensin Converting Enzyme Inhibitors in the First 24 Hours- Route of administration**

Coding Instructions: Indicate if angiotensin converting enzyme inhibitors were given intravenous, PO, or both.

Target Value: N/A

Selections: (1) IV (2) PO (3) Both Supporting Definitions: (none) Source: Definition per CORE team

**Angiotensin Converting Enzyme Inhibitors at Discharge**

Coding Instructions: Indicate if an angiotensin converting enzyme inhibitor was continued or prescribed at discharge.

Note(s): Discharge medications do not need to be recorded for patients who were discharged to

"Other acute care hospital", "Hospice", or "Left against medical advice (AMA)." Target Value: Any occurrence on discharge

Selections: (1) No (2) Yes

Supporting Definitions: (none)

Source: Adapted from AHA Get with the Guidelines ACTION registry

**Angiotensin Converting Enzyme Inhibitors at Discharge –Dose**

Coding Instructions: Indicate the dose of angiotensin converting enzyme inhibitor prescribed at discharge. The name of the agent used should also be mentioned.

Note(s): Discharge medications do not need to be recorded for patients who were discharged to

"Other acute care hospital", "Hospice", or "Left against medical advice (AMA)." Target Value: The highest value on discharge

Selections: (none)

Supporting Definitions: (none) Source: Definition per CORE team

**Documented Reasons for Non-prescription of ACE Inhibitors at Discharge**

Coding Instructions: If the medication was not prescribed, indicate whether the reason was documented.

Target Value: Any documentation in the chart regarding contraindications for the prescription of the medication at discharge.

Selections: (1) No (2) Yes – Allergy (3) Yes – Other (specify) Source: Definition per CORE team

**Angiotensin Receptor Blocker in the First 24 Hours**

Coding Instructions: Indicate if an angiotensin receptor blocker was administered, regardless of location of care (e.g. transferring facility or EMS).

Target Value: Any occurrence between 24 hours before first medical contact and 24 hours after first medical contact

Selections: (1) No (2) Yes

Supporting Definitions: (none)

Source: Adapted from AHA Get with the Guidelines ACTION registry

**Angiotensin Receptor Blockers in the First 24 Hours- Start Date**

Coding Instructions: Indicate the date the initial dose of angiotensin receptor blocker was administered, regardless of location of care (e.g. transferring facility or EMS). If administered more than once, code the first date/time it was administered.

Target Value: The first value between 24 hours before first medical contact and 24 hours after first medical contact.

Selections: (none)

Supporting Definitions: (none)

Source: Adapted from AHA Get with the Guidelines ACTION registry

**Angiotensin Receptor Blockers in the First 24 Hours- Start Time**

Coding Instructions: Indicate the time that the angiotensin receptor blocker was administered in the first 24 hours before or after first medical contact, regardless of location of care (e.g., transferring facility or EMS). If administered more than once, code the first date/time it was administered.

Target Value: The first value between 24 hours before first medical contact and 24 hours after first medical contact.

Selections: (none)

Supporting Definitions: (none)

Source: Adapted from AHA Get with the Guidelines ACTION registry

**Angiotensin Receptor Blockers at Discharge –Dose**

Coding Instructions: Indicate the dose of angiotensin receptor blocker prescribed at discharge. The name of the agent used should also be mentioned.

Note(s): Discharge medications do not need to be recorded for patients who were discharged to

"Other acute care hospital", "Hospice", or "Left against medical advice (AMA)." Target Value: The highest value on discharge

Selections: (none)

Supporting Definitions: (none) Source: Definition per CORE team

**Angiotensin Receptor Blockers at Discharge**

Coding Instructions: Indicate if an angiotensin receptor blocker was continued or prescribed at discharge.

Note(s): Discharge medications do not need to be recorded for patients who were discharged to

"Other acute care hospital", "Hospice", or "Left against medical advice (AMA)." Target Value: Any occurrence on discharge

Selections: (1) No (2) Yes

Supporting Definitions: (none)

Source: Adapted from AHA Get with the Guidelines ACTION registry

**Documented Reasons for Non-prescription of ARBs at Discharge**

Coding Instructions: If the medication was not prescribed, indicate whether the reason was documented.

Target Value: Any documentation in the chart regarding contraindications for the prescription of the medication at discharge.

Selections: (1) No (2) Yes – Allergy (3) Yes – Other (specify)

Source: Definition per CORE team

**Statin in the First 24 Hours**

Coding Instructions: Indicate if a statin was administered, regardless of location of care (e.g. transferring facility or EMS).

Target Value: Any occurrence between 24 hours before first medical contact and 24 hours after first medical contact

Selections: (1) No (2) Yes

Supporting Definitions: (none)

Source: Adapted from AHA Get with the Guidelines ACTION registry

**Statins in the First 24 Hours- Start Date**

Coding Instructions: Indicate the date the initial dose of statin was administered, regardless of location of care (e.g. transferring facility or EMS). If administered more than once, code the first date/time it was administered.

Target Value: The first value between 24 hours before first medical contact and 24 hours after first medical contact.

Selections: (none)

Supporting Definitions: (none)

Source: Adapted from AHA Get with the Guidelines ACTION registry

**Statins in the First 24 Hours- Start Time**

Coding Instructions: Indicate the time that the statin was administered in the first 24 hours before or after first medical contact, regardless of location of care (e.g., transferring facility or EMS). If administered more than once, code the first date/time it was administered.

Target Value: The first value between 24 hours before first medical contact and 24 hours after first medical contact.

Selections: (none)

Supporting Definitions: (none)

Source: Adapted from AHA Get with the Guidelines ACTION registry

**Statins at Discharge**

Coding Instructions: Indicate if a statin was continued or prescribed at discharge.

Note(s): Discharge medications do not need to be recorded for patients who were discharged to

"Other acute care hospital", "Hospice", or "Left against medical advice (AMA)." Target Value: Any occurrence on discharge

Selections: (1) No (2) Yes

Supporting Definitions: (none)

Source: Adapted from AHA Get with the Guidelines ACTION registry

**Documented Reasons for Non-Prescription of Statin at Discharge**

Coding Instructions: If the medication was not prescribed, indicate whether the reason was documented.

Target Value: Any documentation in the chart regarding contraindications for the prescription of the medication at Discharge.

Selections: (1) No

(2) Yes – Allergy

(3) Yes – Other (specify) Source: Definition per CORE team

**Statin at Discharge –Dose**

Coding Instructions: Indicate the dose of statin prescribed at discharge. The agent used should be named, too.

Note(s): Discharge medications do not need to be recorded for patients who were discharged to

"Other acute care hospital", "Hospice", or "Left against medical advice (AMA)." Target Value: The highest value on discharge

Selections: (none)

Supporting Definitions: (none) Source: Definition per CORE team

**Non-statin Lipid Lowering Agents in the First 24 Hours**

Coding Instructions: Indicate if a non-statin lipid-lowering drug was administered, regardless of location of care (e.g. transferring facility or EMS).

Target Value: Any occurrence between 24 hours before first medical contact and 24 hours after

first medical contact Selections: (1) No (2) Yes Supporting Definitions: (none)

Source: Adapted from AHA Get with the Guidelines ACTION registry

**Non-statin Lipid Lowering Agents at Discharge**

Coding Instructions: Indicate if a non-statin lipid-lowering agent was continued or prescribed at discharge.

Note(s): Discharge medications do not need to be recorded for patients who were discharged to

"Other acute care hospital", "Hospice", or "Left against medical advice (AMA)." Target Value: Any occurrence on discharge

Selections: (1) No (2) Yes

Supporting Definitions: (none)

Source: Adapted from AHA Get with the Guidelines ACTION registry

**Nitrates in the First 24 Hours**

Coding Instructions: Indicate if a nitrate was administered, regardless of location of care (e.g. transferring facility or EMS).

Target Value: Any occurrence between 24 hours before first medical contact and 24 hours after first medical contact

Selections: (1) No (2) Yes - If yes, name the drug. Supporting Definitions: (none)

Source: Definition per CORE team

**Nitrate at Discharge**

Coding Instructions: Indicate if a nitrate was continued or prescribed at discharge.

Note(s): Discharge medications do not need to be recorded for patients who were discharged to

"Other acute care hospital", "Hospice", or "Left against medical advice (AMA)." Target Value: Any occurrence on discharge

Selections: (1) No (2) Yes - If yes, name the drug and the dose at discharge

Supporting Definitions: (none) Source: Definition per CORE team

**Ranolazine at Discharge**

Coding Instructions: Indicate if ranolazine was continued or prescribed at discharge.

Note(s): Discharge medications do not need to be recorded for patients who were discharged to

"Other acute care hospital", "Hospice", or "Left against medical advice (AMA)." Target Value: Any occurrence on discharge

Selections: (1) No (2) Yes Supporting Definitions: (none) Source: Definition per CORE team

**Calcium Channel Blockers at Discharge**

Coding Instructions: Indicate if a calcium channel blocker was continued or prescribed at discharge.

Note(s): Discharge medications do not need to be recorded for patients who were discharged to

"Other acute care hospital", "Hospice", or "Left against medical advice (AMA)." Target Value: Any occurrence on discharge

Selections: (1) No (2) Yes - If yes, please name the agent being prescribed. Supporting Definitions: (none)

Source: Definition per CORE team

**Traditional Chinese Medications (TCM) at Discharge**

Coding Instructions: Indicate if a TCM was continued or prescribed at discharge.

Note(s): Discharge medications do not need to be recorded for patients who were discharged to

"Other acute care hospital", "Hospice", or "Left against medical advice (AMA)." Target Value: Any occurrence on discharge

Selections: (1) No (2) Yes -If yes, please name the drug.

Supporting Definitions: (none)

We will note the following seven categories TCPM (traditional Chinese patent medication)

commonly used for AMI in China based on their main ingredient.

1. Danshen or Ginseng or Red Ginseng 2. Ginkgo 3. Sanqi (Panax notoginseng) 4. Hirudin 5. Erigeron breviscapus Extract（Dengzhan Hua 6. Lipid-lowing TCPM (Xuezhikang and Taizhian)

7. Others（Jiuxinwan and Gegengsu）

Source: Definition per China team

**Proton Pump Inhibitor Use**

Coding Instructions: Indicate if a proton pump inhibitor was administered, regardless of location of care (e.g. transferring facility or EMS).

Target Value: Any occurrence the first medical contact and discharge date.

Selections: (1) No (2) Yes - If yes, name the agent being used. Supporting Definitions: (none)

Source: Definition per CORE team

**Glucose Insulin Potassium Solution Use During Hospitalization**

Coding Instructions: Indicate if Glucose Insulin Potassium (GIK) solution was administered during admission to this hospital.

Target Value: Any occurrence after admission to this facility. Selections: (1) No (2) Yes

Supporting Definitions: (none)

Source: Definition per CORE team

**Other Drugs at Discharge**

Coding Instructions: Indicate if other drugs continued or prescribed at discharge.

Note(s): Discharge medications do not need to be recorded for patients who were discharged to

"Other acute care hospital", "Hospice", or "Left against medical advice (AMA)." Target Value: Any occurrence on discharge.

Selections: (1) No (2) Yes - If yes, please name the agent.

Supporting Definitions: If multiple “other drugs” are used, please report each, separately

Source: Definition per CORE team

**Drug allergy**

Coding Instructions: Indicate if the patient has any drug allergy

Target Value: N/A Selections: (1) No (2) Yes Supporting Definitions: (none) Source: Definition per CORE team

**CARDIAC CATHETERIZATION AND RELATED COMPLICATIONS**

**Diagnostic Catheterization or Diagnostic Coronary Angiography**

Coding Instructions: Indicate if the patient had a diagnostic coronary angiography procedure. Target Value: Any occurrence between arrival at first facility and discharge

Selections: (1) No (2) Yes

Supporting Definitions: Diagnostic coronary angiography is defined as the passage of a catheter into the aortic root or other great vessels for the purpose of angiography of the native coronary arteries or bypass grafts supplying native coronary arteries.

Source: NCDR; adapted from AHA Get with the Guidelines ACTION registry

**Catheterization Laboratory Arrival Date and Time**

Coding Instructions: Indicate the date the patient arrived to the cath lab, as documented in the medical record, as well as the time of arrival

Target Value: The first value between arrival at this facility and discharge

Selections: (none)

Supporting Definitions: Indicate the time (hours: minutes) using the military 24-hour clock, beginning at midnight (0000 hours). If an arterial sheath is already in place, use the time of the introduction of a catheter or the time the sheath was exchanged.

Source: Adapted from AHA Get with the Guidelines ACTION registry and from VIRGO registry

**Time of Arterial Access**

Coding Instructions: Indicate the time arterial access was obtained.

Note(s): Time of Procedure: The time arterial access was obtained is defined as the time at which local anesthetic was first administered for vascular access, or the time of the first attempt at vascular access for the cardiac catheterization (use whichever is earlier).

Target Value: N/A Selections: (none)

Supporting Definitions: Indicate the time (hours: minutes) using the military 24-hour clock,

beginning at midnight (0000 hours).

Source: Adapted from AHA Get with the Guidelines CATH-PCI registry

**Arterial Access Site**

Coding Instructions: Indicate the primary location of percutaneous entry. If more than one entry site was used, choose the site that was used to perform the majority of the procedure.

Target Value: N/A

Selections: Choose one of the following:

a. Femoral; mark “Femoral” if percutaneous puncture of either femoral artery. b. Radial; mark “Radial” if percutaneous radial approach.

c. Brachial; mark “Brachial” if either a cutdown or percutaneous puncture of either brachial artery.

d. Other; mark “Other” if percutaneous entry other than femoral, brachial, or radial approaches to the cardiovascular system.

Supporting Definitions: (none)

Source: Adapted from AHA Get with the Guidelines ACTION registry

**Arterial Dominance**

Coding Instructions: Indicate the dominance of the coronary anatomy (whether the posterior descending artery comes from the right or left vessel system).

Selections: Selection Text Definition

Left dominance is present when the posterior descending artery (PDA) and posterolateral artery (PLA) arise from the left circumflex artery.

Right dominance is present when the posterior descending artery (PDA) and

posterolateral artery (PLA) arises from the right coronary artery.

Co-dominance is present when the right coronary artery supplies the posterior descending artery (PDA) and the circumflex supplies the posterolateral artery (PLA). Thus, there is approximately equal contribution to the inferior surface of the left ventricle from both the left circumflex and right coronary arteries.

If not reported, select “unrecorded”. Supporting Definitions: (none)

Source: NCDR; Adapted from the CathPCI registry

**Stenosis Percent**

Coding Instructions: Indicate the best estimate of most severe percent stenosis in any coronary artery.

Target Value: The highest value between arrival at first facility and discharge

Selections: Include:

a. Left Main Artery (LM)

b. Proximal LAD

c. Mid/Distal LAD, Diag Branches

d. CIRC, OMs, LPDA, LPL Branches e. RCA, RPDA, RPL, AM Branches

f. Ramus

Supporting Definitions: Does not include collateral circulation.

Provide the most severe stenosis for the vessel that is primarily providing perfusion to the myocardium in that territory. (Ex. If a patient's mid LAD is 100% and a graft provides revascularization to that territory of the heart, code the % stenosis of the graft. If the same patient has an open graft, and a 70% stenosis of the 2nd diagonal, code 70% since that is the

most severe stenosis % for that territory of the myocardium.) In instances where multiple lesions are present, enter the single highest percent stenosis noted. If no stenosis, then enter 0%. Stenosis: Stenosis represents the percentage diameter reduction, from 0 to 100, associated

with the identified vessels. Percent stenosis at its maximal point is estimated to be the amount of reduction in the diameter of the "normal" reference vessel proximal to the lesion.

Source: NCDR; Adapted from AHA Get with the Guidelines ACTION registry

**Type of Contrast Dye Used**

Coding Instructions: Indicate the name of radiographic contrast agent used for angiography. Target Value: N/A

Selections: (1) Urografin (2) Iopamidol (3) Iopromide (4) Iohexol (5) Iodixanol (6) Iomeprol (7) Ioversol (8) Other (9) Unrecorded

Supporting Definitions: (none)

Source: Adapted from VIRGO registry

**Total Volume of Contrast Dye Used**

Coding Instructions: Indicate the total volume (ml) of contrast dye used during angiography/PCI. Target Value: Any occurrence between arrival at admitting hospital and discharge

Selections: (none)

Supporting Definitions: (none)

Source: Adapted from VIRGO registry

**Complication- Contrast Reaction**

Coding Instructions: Indicate whether patient experienced a contrast reaction during the cath lab visit or after the lab visit but before discharge.

Target Value: Any occurrence between PCI and discharge

Selections: (1) No (2) Yes

Supporting Definition: Contrast reaction is defined as at least one of the following:

a. Anaphylaxis-including bronchospasm and/or vascular collapse b. Urticaria

c. Hypotension-prolonged depression of blood pressure below 70mm Hg. Source: Adapted from VIRGO registry

**Percutaneous Coronary Intervention (PCI)**

Coding Instructions: Indicate if the patient had percutaneous coronary intervention Target Value: Any occurrence between arrival at this facility and discharge Selections: (1) No (2) Yes

Supporting Definitions: Percutaneous coronary intervention (PCI) is the placement of an angioplasty guide wire, balloon, or other device (e.g. stent, atherectomy, brachytherapy, or thrombectomy catheter) into a native coronary artery or coronary artery bypass graft for the purpose of mechanical coronary revascularization.

Source: Adapted from AHA Get with the Guidelines ACTION registry

**Refusal of Percutaneous Coronary Intervention**

Coding Instructions: Indicate if there is documentation of patient refusal for percutaneous coronary intervention

Target Value: Any occurrence from the beginning of hospital stay to discharge

Selections: (1) No (2) Yes Supporting Definitions: (none) Source: Definition per CORE team

**Did Guidewire Cross Lesion?**

Coding Instructions: Indicate if the guidewire successfully crossed the lesion. Target Value: N/A

Selections: (1) No (2) Yes

Supporting Definitions: (none)

Source: Adapted from NCDR CathPCI

**Time Guidewire Crossed Lesion**

Coding Instructions: Indicate the time of guidewire introduction or time it crossed target lesion. Target Value: N/A

Selections: (none)

Supporting Definitions: (none)

Source: Adapted from NCDR CathPCI

**First Device Activation Date and Time**

Coding Instructions: Indicate the date and time the first device was activated regardless of type of device used.

Note(s): Use the earliest time from the following:

1. Time of the first balloon inflation.

2. Time of the first stent deployment.

3. Time of the first treatment of lesion (AngioJet or other thrombectomy/aspiration device, laser, rotational atherectomy).

4. If the lesion cannot be crossed with a guidewire or device (and thus none of the above apply), use the time of guidewire introduction.

This is a process measure about the timeliness of treatment. It is NOT a clinical

outcomes measure based on TIMI flow or clinical reperfusion. It does not matter whether the baseline angiogram showed TIMI 3 flow or if the final post-PCI angiogram showed TIMI 0 flow. What is being measured is the time of the first mechanical treatment of the culprit lesion, not the time when TIMI 3 flow was (or was not) restored.

Indicate the time (hours:minutes) using the military 24-hour clock, beginning at midnight

(0000 hours).

This element is referenced in The Joint Commission AMI Core Measures AMI-8, AMI-8a. Target Value: N/A

Selections: (none)

Supporting Definitions: Indicate the time (hours: minutes) using the military 24-hour clock, beginning at midnight (0000 hours).

Source: Adapted from AHA Get with the Guidelines CATH-PCI registry

**Time of the First Balloon Inflation**

Coding Instructions: Indicate the date and time of the first balloon inflation during percutaneous coronary intervention

Target Value: N/A

Selections: (none)

Supporting Definitions: Indicate the time (hours: minutes) using the military 24-hour clock, beginning at midnight (0000 hours).

Source: Adapted from VIRGO registry

**Time of the First Stent Deployment**

Coding Instructions: Indicate the date and time of the first stent deployment during percutaneous coronary intervention

Target Value: N/A Selections: (none)

Supporting Definitions: Indicate the time (hours: minutes) using the military 24-hour clock, beginning at midnight (0000 hours).

Source: Adapted from VIRGO registry

**Stent(s) Placed**

Coding Instructions: Indicate if a stent or stents were placed in the affected coronary artery. Target Value: Any occurrence between arrival at this facility and discharge

Selections: (1) No (2) Yes

Supporting Definitions: (none)

Source: Adapted from AHA Get with the Guidelines ACTION registry

**Total Number of Stent(s) Placed**

Coding Instructions: Indicate the number of stents placed in the affected coronary artery. Target Value: Any occurrence between arrival at this facility and discharge

Selections: (none)

Supporting Definitions: (none)

Source: Adapted from AHA Get with the Guidelines ACTION registry

**Bare Metal Stent Implanted**

Coding Instructions: Indicate if one or more bare metal stents were implanted during PCI. Target Value: Any occurrence between arrival at this facility and discharge

Selections: (1) No (2) Yes

Supporting Definitions: (none)

Source: Adapted from AHA Get with the Guidelines ACTION registry

**Drug Eluting Stent Implanted**

Coding Instructions: Indicate if one or more drug eluting stents were implanted during PCI. Target Value: Any occurrence between arrival at this facility and discharge

Selections: (1) No (2) Yes

Supporting Definitions: (none)

Source: Adapted from AHA Get with the Guidelines ACTION registry

**Intra-aortic Balloon Pump (IABP) Insertion**

Coding Instructions: Indicate whether the patient received an intra-aortic balloon pump Target Value: Any occurrence between arrival at admitting hospital and discharge Selections: (1) No (2) Yes

Supporting Definition: (none)

Source: Adapted from VIRGO registry

**Closure Method**

Coding Instructions: Indicate the closure method after percutaneous coronary intervention (PCI). Target Value: Any occurrence between arrival at admitting hospital and discharge

Selections:

a. Seal (Angioseal, Vasoseal)

b. Suture

c. Manual Compression d. Other

e. Unrecorded

Supporting Definition: The closure device is a device used at the arterial access site at the end of the procedure to facilitate hemostasis without need for manual compression.

Source: Adapted from AHA Get with the Guidelines ACTION registry

**PCI Complication-Tamponade**

Coding Instructions: Indicate if the patient experienced a cardiac tamponade associated with the cardiac catheterization/PCI.

Target Value: Any occurrence between PCI and discharge

Selections: (1) No (2) Yes

Supporting Definition: Mark “Yes” if there was fluid in the pericardial space compromising cardiac filling and requiring intervention during the cath lab visit or after lab visit until discharge (or before any subsequent lab visits). This should be documented by either:

a. Echo showing pericardial fluid and signs of tamponade such as right heart

compromise; or

b. Systemic hypotension due to pericardial fluid compromising cardiac function. Source: Adapted from VIRGO registry

**PCI Complication-Peripheral Embolization**

Coding Instructions: Indicate whether patient experienced peripheral embolization after PCI. Target Value: Any occurrence between PCI and discharge

Selections: (1) No (2) Yes

Supporting Definition: Mark “Yes” if a peripheral embolization occurred distal to the

arterial access site during the procedure or after lab visit but before any subsequent lab visits, requiring therapy. Peripheral embolization is defined as a loss of distal pulse, pain and/or discoloration (especially the toes). This can include cholesterol emboli.

Source: Adapted from VIRGO registry

**PCI Complication-Access Site Arteriovenous Fistula**

Coding Instructions: Indicate whether patient experienced access site arteriovenous fistula during the cath lab visit or after lab visit until discharge.

Target Value: Any occurrence between PCI and discharge

Selections: (1) No (2) Yes

Supporting Definition: (none)

Source: Adapted from VIRGO registry

**PCI Complication-Access Complication Requiring Surgery/Intervention**

Coding Instructions: Indicate whether patient experienced access site occlusion at the site of percutaneous entry during the procedure or after lab visit but before any subsequent lab visits. Target Value: Any occurrence between PCI and discharge

Selections: (1) No (2) Yes

Supporting Definition: Access site occlusion if defined as: Total obstruction of the artery usually by thrombus (but may have other causes) usually at the site of access requiring surgical repair. Occlusions may be accompanied by absence of palpable pulse or Doppler.

Source: Adapted from VIRGO registry

**PCI Complication-Retroperitoneal Bleeding**

Coding Instructions: Indicate whether patient experienced retroperitoneal bleeding after lab visit. Target Value: Any occurrence between PCI and discharge

Selections: (1) No (2) Yes

Supporting Definition: Mark “Yes” if retroperitoneal bleeding occurred during or after the cath lab visit until discharge. The bleeding should require a transfusion and/or prolong the hospital stay, and/or cause a drop in hemoglobin > 3.0 gm/dl.

Source: Adapted from VIRGO registry

**Repeat PCI**

Coding Instructions: Indicate whether patient had a second PCI during hospital stay

Target Value: Any occurrence after first PCI and before discharge

Selections: (1) No (2) Yes

Supporting Definition: (none)

Source: Adapted from VIRGO registry

**Stent Thrombosis**

Coding Instructions: Indicate whether patient experienced in-stent thrombosis after PCI Target Value: Any occurrence after the first PCI and before discharge

Selections: (1) No (2) Yes

Supporting Definition: (none)

Source: Adapted from VIRGO registry

**Reasons for Repeat PCI**

Coding Instructions: Indicate the reasons for the repeat coronary angiography/ PCI during the hospital stay.

Target Value: Any occurrence after first PCI and discharge

Selections:

1. Staged procedure

2. Ongoing or recurrent ischemia

3. Other

Supporting Definition: Mark “Staged procedure” if second PCI was planned during hospitalization for residual stenoses. Mark “Ongoing or recurrent ischemia/angina” if the patient had recurrent symptoms (e.g. angina), signs (e.g. dynamic ECG changes) or biomarker elevation consistent with ischemia.

Source: Definition per CORE team and Adapted from VIRGO registry

**In-Hospital Implantation of a Permanent Pacemaker Device**

Coding Instructions: Indicate if the patient had a permanent pacemaker implanted during the hospital stay.

Target Value: Any occurrence between arrival at admitting hospital and discharge

Selections: (1) No (2) Yes

Supporting Definitions: Permanent pacemaker includes single-chamber, dual chamber, and biventricular pacemakers. It does not include temporary transcutaneous pacemaker. Source: Adapted from VIRGO registry

**Date of In-Hospital Permanent Pacemaker Device Implantation**

Coding Instructions: Indicate date that permanent pacemaker device was placed. Target Value: The first value between arrival at first facility and discharge Selections: N/A

Supporting Definition: (none)

Source: Definition per CORE team

**In-Hospital Implantation of an Automatic Implantable Cardioverter Defibrillator (AICD)** Coding Instructions: Indicate whether patient received an implantable cardioverter defibrillator at any time during hospital stay.

Target Value: N/A Selections: (1) No (2) Yes Supporting Definitions: (none)

Source: Adapted from VIRGO registry

**Date of In-Hospital Automatic Implantable Cardioverter Defibrillator (AICD) Implantation** Coding Instructions: Indicate date that Automatic Implantable Cardioverter Defibrillator was placed

Target Value: The first value between arrival at first facility and discharge

Selections: N/A

Supporting Definition: (none) Source: Definition per CORE team

**Coronary Artery Bypass Grafting (CABG)**

Coding Instructions: Indicate if the patient had a CABG

Target Value: Any occurrence between arrival at this facility and discharge

Selections: (1) No (2) Yes

Selections: (none)

Supporting Definitions: (none)

Source: Adapted from VIRGO registry

**Refusal of Coronary Artery Bypass Grafting**

Coding Instructions: Indicate if there is documentation of patient refusal for coronary artery

bypass grafting

Target Value: Any occurrence from the beginning of hospital stay to discharge

Selections: (1) No (2) Yes (3) Unrecorded

Supporting Definitions: (none) Source: Definition per CORE team

**CABG Date**

Coding Instructions: Indicate the date of the coronary artery bypass graft (CABG) surgery. Target Value: The first value between arrival at this facility and discharge

Selections: (none)

Supporting Definitions: (none)

Source: Definition as per CORE team

**CABG Time**

Coding Instructions: Indicate the time of the coronary artery bypass graft (CABG) surgery. Target Value: The first value between arrival at this facility and discharge

Selections: (none)

Supporting Definitions: (none)

Note(s): The time of the procedure is the time to the nearest minute (using 24-hour clock), that the skin incision or its equivalent was made to start the surgical procedure

Source: Adapted from VIRGO registry

**SUMMARY OF IN-HOSPITAL EVENTS In-Hospital Bleeding**

Coding Instructions: Indicate if the patient had a bleeding event during hospitalization. Target Value: Any occurrence mentioned in the chart from arrival to the facility to discharge Selections: (1) No (2) Yes

Supporting Definitions: If yes, please indicate the time and date.

Source: Definition per CORE team

**Location of Bleeding**

Coding Instructions: Indicate the location of bleeding. Target Value: N/A

Selections: (1) Access site (2) Intracranial (3) Intraocular (4) Intraspinal (5) Retroperitoneal (6) Pericardial (7) Gastrointestinal (8) Genitourinary (9) Other (specify)

Supporting Definitions: Access site bleeding is marked when bleeding happens at the site of vascular access. Intracranial bleeding includes intracerebral and subdural bleeding. Please record each site separately if more than one applies.

Source: Definition per CORE team

**Hypovolemic/Hemorrhagic Shock after Bleeding**

Coding Instructions: Indicate if bleeding led to hypovolemic or hemorrhagic shock. Target Value: Any occurrence during the hospital stay

Selections: (1) No (2) Yes (3) Unknown

Supporting Definitions: In patients who bled during the hospital stay, evidence in the chart may suggest hypovolemic shock:

A) Physician report of hypovolemic shock in the chart

B) Post-bleeding systolic hypotension (peak systolic pressure<90mmHg) or a reduction of >40mmHg in systolic blood pressure plus evidence of organ hypoperfusion,

which is not responsive to administration of plasma expanders or packed RBCs. Source: Definition per CORE team

**Interventions for Management of Bleeding**

Coding Instructions: Indicate the intervention(s) used to manage bleeding. Target Value: Any occurrence after the bleeding event.

Selections: (1) whole blood transfusion (2) packed red cell transfusion (3) local compression (4)

surgical intervention, including open surgery, closure or endoscopic interventions (5) others (6)

none (7) unrecorded

Supporting Definitions: Access site bleeding is marked when bleeding happens at the site of vascular access. Intracranial bleeding includes intracerebral and subdural bleeding.

Note(s): Please record each intervention separately if more than one was applied. Source: Definition per CORE team

**Blood Transfusion**

Coding Instructions: Indicate if the patient was transfused with whole blood or any of its components.

Target Value: Any occurrence between first medical contact and discharge

Selections: (1) No (2) Yes (3) Unrecorded

If Yes, specify:

Red blood cell

Platelet

Blood plasma

Whole Blood

Other (specify)______ Supporting Definition: (none) Source: Definition per CORE team

**Bleeding Date Hb, HCT, Platelet Count, PT, aPTT, and INR**

Coding Instructions: Indicate the bleeding date Hb, HCT, Platelet Count, PT, aPTT, and INR. Target Value: In two separate time points

A) Last available laboratory tests before the bleeding event happened

B) Nadir of the aforementioned laboratory tests after the bleeding event Selections: Varies for each, please refer to the definitions in the laboratory tests section. Supporting Definitions: Such information should only be collected for patients with bleeding Source: Definition per CORE team

**In-Hospital Dialysis**

Coding Instructions: Indicate if the patient received dialysis during the hospital stay

Target Value: Any occurrence from the hospital stay to discharge

Selections: (1) No (2) Yes

Supporting Definitions: Yes includes hemodialysis, peritoneal dialysis, or both. Source: Adapted from VIRGO registry

**In-Hospital Cardiac Tamponade**

Coding Instructions: Indicate if cardiac tamponade occurred during the hospital stay

Target Value: Any occurrence from the hospital stay to discharge

Selections: (1) No (2) Yes

Supporting Definitions: A clinical syndrome caused by the accumulation of fluid in the pericardial space, resulting in reduced ventricular filling and subsequent hemodynamic compromise. Source: Definition per CORE team

**In-Hospital Venous Thromboembolism**

Coding Instructions: Indicate if in-hospital venous thromboembolism (VTE) was diagnosed during the hospital stay

Target Value: Any occurrence from the beginning of the hospital stay to discharge

Selections: (1) No (2) Yes - If yes, please indicate the date

Supporting Definitions: Venous thromboembolism (VTE) is comprised of deep vein thrombosis ([DVT]; i.e. development of blood clots in the deep veins of lower extremity or upper extremity) and pulmonary embolism ([PE]; i.e. migration of the clots to the pulmonary arteries. The clots can clog the pulmonary arteries, impairing the gas exchange in the lungs. PE is associated with symptoms such as dyspnea and chest pain and can be fatal). For any patient that was diagnosed with DVT, or with PE, or with both during the index admission, please mark “Yes”. Source: Definition per CORE team

**In-Hospital Deep Vein Thrombosis (DVT)**

Coding Instructions: Indicate if in-hospital DVT was diagnosed during the hospital stay Target Value: Any occurrence from the beginning of the hospital stay to discharge Selections: (1) No (2) Yes - If yes, please indicate the date

Supporting Definitions: DVT refers to development of blood clots in the deep veins of lower extremity or upper extremity migration of the clots to the pulmonary arteries. The symptoms and signs include extremity pain, warmness, swelling, a palpable venous cord, and tenderness. The diagnosis is made by ultrasonography or venography. Mark “Yes” if there is physician documentation for the diagnosis.

Source: Definition per CORE team

**In-Hospital Pulmonary Embolism (PE)**

Coding Instructions: Indicate if in-hospital PE was diagnosed during the hospital stay Target Value: Any occurrence from the beginning of the hospital stay to discharge Selections: (1) No (2) Yes - If yes, please indicate the date

Supporting Definitions: PE refers to a clinical condition resulting from migration of the clots

(rarely other material) to the pulmonary arteries. The clots can clog the pulmonary arteries, impairing the gas exchange in the lungs. PE is associated with symptoms such as dyspnea, hemoptysis and chest pain and can be fatal. The diagnosis could be made by ventilation- perfusion (V/Q) scanning, computed tomography pulmonary angiography, or conventional pulmonary angiography. Mark “Yes” if there is physician documentation for the diagnosis. Source: Definition per CORE team

**In-Hospital Infection**

Coding Instructions: Indicate if in-hospital infection occurred during the hospital stay Target Value: Any occurrence from the beginning of hospital stay to discharge Selections: (1) No (2) Yes If yes, please indicate the date

Supporting Definitions: (none) Source: Definition per CORE team

**In-Hospital Infection –Site**

Coding Instructions: Indicate the site of in-hospital infection

Target Value: N/A

Selections: (1) Pulmonary (2) Genitourinary (3) Gastrointestinal (4) Skin (5) Surgical site/procedure site (6) Other (7) Unrecorded

Supporting Definitions: (none)

Source: Definition per CORE team

**In-Hospital Cardiogenic Shock**

Coding Instructions: Indicate if cardiogenic shock occurred during the hospital stay

Target Value: Any occurrence from the hospital stay to discharge

Selections: (1) No (2) Yes- If yes, please indicate the date and time

Supporting Definitions: Cardiogenic shock is defined as a sustained (>30 minutes) episode of systolic blood pressure <90 mm Hg, and/or cardiac index <2.2 L/min/m2 determined to be secondary to cardiac dysfunction, and/or the requirement for parenteral inotropic or vasopressor agents or mechanical support (e.g., IABP, extracorporeal circulation, ventricular assist devices) to maintain blood pressure and cardiac index above those specified levels.

Source: Acute Coronary Syndromes Data Standards (JACC 2001 38: 2114 - 30), Adapted from

AHA Get with the Guidelines ACTION registry

**In-Hospital Cardiac Rupture**

Coding Instructions: Indicate if there is physician documentation of in-hospital cardiac rupture

Target Value: Any occurrence from the beginning of hospital stay to discharge

Selections: (1) No (2) Yes Supporting Definitions: (none) Source: Definition per CORE team

**In-Hospital Recurrent Angina/Recurrent Myocardial Infarction**

Coding Instructions: Indicate if there is physician documentation of in-hospital recurrent angina or recurrent myocardial infarction

Target Value: Any occurrence from the beginning of hospital stay to discharge

Selections: (1) No (2) Yes

Supporting Definitions: Physician documentation of recurrent angina, or recurrent myocardial infarction, or both, warrants a “Yes” answer.

Source: Definition per CORE team

**In-Hospital Ventricular Tachycardia/Ventricular Fibrillation**

Coding Instructions: Indicate if there is physician documentation of in-hospital ventricular tachycardia or ventricular fibrillation

Target Value: Any occurrence from the beginning of hospital stay to discharge

Selections: (1) No (2) Yes

Supporting Definitions: Physician documentation of ventricular tachycardia or ventricular fibrillation, or both, warrants a “Yes” answer.

Source: Definition per CORE team

**In-Hospital Papillary Muscle Rupture**

Coding Instructions: Indicate if there is physician documentation of in-hospital papillary muscle rupture.

Target Value: Any occurrence from the beginning of hospital stay to discharge

Selections: (1) No (2) Yes Supporting Definitions: (none) Source: Definition per CORE team

**In-Hospital Ventricular Septal Perforation**

Coding Instructions: Indicate if there is physician documentation of ventricular septal perforation. Target Value: Any occurrence from the beginning of hospital stay to discharge

Selections: (1) No (2) Yes

Supporting Definitions: (none)

Source: Definition per CORE team

**In-Hospital Acute Renal Failure**

Coding Instructions: Indicate if there is physician documentation or report of in-hospital acute renal failure

Target Value: Any occurrence from the beginning of hospital stay to discharge

Selections: (1) No (2) Yes

Supporting Definitions: Acute renal failure is defined as physician documentation of acute renal failure

Source: Definition per CORE team

**In-Hospital Peripheral Embolization**

Coding Instructions: Indicate if there is physician documentation or report of in-hospital peripheral embolization

Target Value: Any occurrence from the beginning of hospital stay to discharge

Selections: (1) No (2) Yes Supporting Definitions: (none) Source: Definition per CORE team

**In-Hospital Death**

Coding Instructions: Indicate if the patient died during hospital stay.

Target Value: Any occurrence between arrival at this facility and discharge

Selections: (1) No (2) Yes

Supporting Definitions: (none)

Source: Adapted from AHA Get with the Guidelines ACTION registry

**In-Hospital Death Date**

Coding Instructions: Indicate the date of patient’s death. Target Value: The value on death date.

Selections: (none)

Supporting Definitions: (none) Source: Definition per CORE team

**Autopsy**

Coding Instructions: Indicate if an autopsy of patient was performed after death. Target Value: N/A

Selections: (1) No (2) Yes (3) Unrecorded

Supporting Definition: Autopsy is a postmortem examination to discover the cause of death or the extent of disease

Source: Definition per CORE team; Oxford English Dictionary

**In-Hospital Cardiac Arrest**

Coding Instructions: Indicate if the patient experienced an episode of cardiac arrest in your facility.

Target Value: Any occurrence between arrival at this facility and discharge

Selections: (1) No (2) Yes

Supporting Definitions: 'Sudden' cardiac arrest is the sudden cessation of cardiac activity so that the victim becomes unresponsive, with no normal breathing and no signs of circulation. If corrective measures are not taken rapidly, this condition progresses to sudden death. Cardiac arrest should be used to signify an event as described above that is reversed, usually by CPR,

and/or defibrillation or cardioversion, or cardiac pacing. Sudden cardiac arrest is not the same as sudden cardiac death. Sudden cardiac death describes a fatal event.

Source: ACC/AHA/HRS 2006 Key Data Elements and Definitions for Electrophysiological

Studies and Procedures

**In-Hospital Cardiac Arrest Date**

Coding Instructions: Indicate the date of the cardiac arrest.

Target Value: The first value between arrival at this facility and discharge

Selections: (none)

Supporting Definitions: (none)

**In-Hospital Cerebrovascular accident (CVA)/Stroke**

Coding Instructions: Indicate if the patient experienced a stroke or CVA in your facility. Target Value: Any occurrence between arrival at this facility and discharge

Selections: (1) No (2) Yes

Supporting Definitions: Stroke: A stroke or cerebrovascular accident is defined as loss of neurological function caused by an ischemic or hemorrhagic event with residual symptoms at least 24 hours after onset or leading to death.

Source: Acute Coronary Syndromes Data Standards (JACC 2001 38: 2114 - 30)

**In-Hospital Stroke Date**

Coding Instructions: Indicate the date of onset of stroke. If a stroke occurs during sleep, last awake time may be used.

Target Value: The first value between arrival at this facility and discharge

Selections: (none)

Supporting Definitions: (none)

Source: Adapted from AHA Get with the Guidelines ACTION registry

**In-Hospital Hemorrhagic Stroke**

Coding Instructions: Indicate if the patient experienced a hemorrhagic stroke with documentation on imaging.

Target Value: Any occurrence between arrival at this facility and discharge

Selections: (1) No (2) Yes

Supporting Definitions: Hemorrhagic Stroke: A hemorrhagic stroke requires documentation on imaging (e.g. CT scan or MRI of hemorrhage in the cerebral parenchyma, or a subdural or subarachnoid hemorrhage). Evidence of hemorrhagic stroke obtained from lumbar puncture, neurosurgery, or autopsy can also confirm the diagnosis.

Source: Acute Coronary Syndromes Data Standards (JACC 2001 38: 2114 - 30)

**In-Hospital Unspecified Stroke**

Coding Instructions: Indicate if the patient experienced an unspecified stroke with documentation on imaging.

Target Value: Any occurrence between arrival at this facility and discharge

Selections: (1) No (2) Yes

Supporting Definitions: An unspecified stroke can be defined as stroke without documentation on imaging. Evidence of stroke obtained from clinical symptoms.

Source: Definition per CORE team

**In-Hospital (New Onset) Heart Failure**

Coding Instructions: Indicate if there is physician documentation or report on development of heart failure during hospital stay.

Target Value: Any occurrence between first medical contact and arrival at this facility

Selections: (1) No (2) Yes

Supporting Definitions: Heart failure is defined as physician documentation or report of any of the following clinical symptoms of heart failure described as unusual dyspnea on light exertion, recurrent dyspnea occurring in the supine position, fluid retention; or the description of rales, jugular venous distension, pulmonary edema on physical exam, or pulmonary edema on chest x-ray presumed to be cardiac dysfunction. A low ejection fraction without clinical evidence of heart failure does not qualify as heart failure.

Source: Acute Coronary Syndromes Data Standards (JACC 2001 38: 2114 - 30), The Society of

Thoracic Surgeons

**In-Hospital Atrial Fibrillation or Flutter**

Coding Instructions: Indicate if patient was diagnosed with atrial fibrillation or flutter during this admission.

Target Value: Any occurrence between arrival at this facility and discharge

Selections: (1) No (2) Yes

Supporting Definitions: Fibrillation of > 30 seconds, which presents as supraventricular complexes at an irregular rhythm and no obvious P waves on ECG; or Flutter presents as identically recurring regular sawtooth flutter waves on ECG and evidence of continual electrical activity.

Note(s): Code "No" If there is no documentation of atrial arrhythmias during hospitalization, it is acceptable to code "No"

Source: Adapted from AHA Get with the Guidelines ACTION registry

**Smoking Cessation Counseling**

Coding Instructions: Indicate if there was documentation in the medical record that smoking cessation advice or counseling was given during this admission.

Note(s): This element is referenced in The Joint Commission AMI Core Measures AMI-4. Target Value: Any occurrence between arrival at this facility and discharge

Selections: (1) No (2) Yes

Supporting Definitions: (none)

Source: Adapted from AHA Get with the Guidelines ACTION registry

**Discharge Status (or Disposition)**

Coding Instructions: Indicate the documented nature of the patient’s discharge from the hospital. Target Value: Any value at discharge

Selections:

(1) Patient or Relatives Demand Transfer to another Hospital

(2) Physician Suggests Transfer to another Hospital

(3) Patient Left against Medical Advice

(4) Other

(5) None of the Above is Recorded

Supporting Definition: The selection “Other” includes discharge without transfer to another hospital.

Source: Definitions per China team

**Discharge Suggestions**

Coding Instructions: Indicate if there was documentation of the following recommendations at discharge

Target Value: as below

Selections:

a. Dual antiplatelet therapy (aspirin and a thienopyridine) (1) No (2) Yes If yes, specify the

duration

b. Regular blood lipid assessment (1) No (2) Yes

c. Dietary improvement (1) No (2) Yes

d. Weight reduction (1) No (2) Yes

e. Smoking cessation (1) No (2) Yes

f. Regular exercise (1) No (2) Yes

g. PCI (1) No (2) Yes

h. CABG (1) No (2) Yes

i. None of the Above Is Recorded (1) No (2) Yes

Supporting Definitions: (none) Source: Definition per CORE team

**Discharge Diagnosis (Related to Coronary Heart Disease)**

Coding Instructions: Indicate the discharge diagnosis mentioned in the chart

Note(s): Marks as many of the choices that apply.

Selections: Coronary Heart Disease, Acute Coronary Syndrome, Acute Myocardial Infarction, Acute Extensive Anterior Myocardial Infarction, Acute Anterior Myocardial Infarction, Acute Septal Myocardial Infarction, Acute Inferior Myocardial Infarction, Acute Lateral Myocardial Infarction, Acute Posterior Myocardial Infarction, Acute Right Ventricular Myocardial Infarction, Acute Non-ST-Elevation Myocardial Infarction, Acute ST-Elevation Myocardial Infarction, Subendocardial Myocardial Infarction, Acute Myocardial Infarction Suspected, Previous Q-wave Myocardial Infarction, Unstable Angina Pectoris (Variant Angina Pectoris, Resting Angina, Supine Angina, Worsening Angina, Post-Infarction Angina), Stable Angina Pectoris, Angina (Unrecorded Subtype)

Supporting Definition: If none of the above options is noted in the chart, mark “None of the above Is Recorded”.

Source: Definition per CORE team

**Discharge Diagnosis (Unrelated to Coronary Heart Disease)**

Coding Instructions: Indicate the discharge diagnosis mentioned in the chart

Note(s): Marks as many of the choices that apply.

Selections: Cardiac rupture, papillary muscle rupture, ventricular septal perforation, cardiac tamponade, cardiogenic shock, cardiac arrest, atrial fibrillation/flutter, ventricular tachycardia/fibrillation, heart failure, gastrointestinal bleeding, genitourinary bleeding, intracranial/subdural bleeding, retroperitoneal bleeding, access site bleeding, pericardial bleeding, bleeding (unspecified), hemorrhagic shock, venous thromboembolism, pulmonary embolism, deep vein thrombosis, peripheral embolization, access site arteriovenous fistula, ischemic stroke, hemorrhagic stroke, stroke (unspecified), pneumonia, COPD exacerbation, acute renal failure, chronic renal failure, dialysis, infection, septicemia, contrast reaction, dyslipidemia, hypertension, diabetes mellitus, diabetic nephropathy, trauma, hepatitis, cirrhosis, anemia.

Supporting Definition: If none of the above options is noted in the chart, mark “None of the above Is Recorded”.

Source: Definition per CORE team

**International Classification of Diseases (ICD) Codes for Discharge Diagnoses**

Coding Instructions: For every discharge diagnosis, record the associated ICD code

Target Value: This should be performed for every diagnosis

Selections: (1) ICD 9; specify value (2) ICD 10; specify value (3) Unrecorded

Supporting Definition: (none) Source: Definition per CORE team

**Date of Hospital Discharge**

Coding Instructions: Indicate the date on which the patient was discharged from the hospital

Selections: (none)

Supporting Definitions: (none) Source: Definition per CORE team

**Transferred to Outside Facility**

Coding Instructions: Indicate if the patient was transferred to an outside facility after initial presentation to your hospital.

Target Value: N/A Selections: (1) No (2) Yes Supporting Definitions: (none) Source: Definition per CORE team
